# Hydroamination of alkynes catalyzed by NHC-Gold(I) complexes: the non-monotonic effect of substituted arylamines on the catalyst activity

**DOI:** 10.3389/fchem.2023.1260726

**Published:** 2023-12-06

**Authors:** Marco Sirignano, Assunta D’Amato, Chiara Costabile, Annaluisa Mariconda, Alessandra Crispini, Francesca Scarpelli, Pasquale Longo

**Affiliations:** ^1^ Department of Chemistry and Biology “A. Zambelli”, University of Salerno, Fisciano, Italy; ^2^ Department of Science, University of Basilicata, Potenza, Italy; ^3^ Department of Chemistry and Chemical Technologies, University of Calabria, Arcavacata Di Rende, Italy

**Keywords:** N-heterocyclic carbene, gold, hydroamination, alkynes, DFT modelling

## Abstract

Imines are valuable key compounds for synthesizing several nitrogen-containing molecules used in biological and industrial fields. They have been obtained, as highly regioselective Markovnikov products, by reacting several alkynes with arylamines in the presence of three new N-Heterocyclic carbene gold(I) complexes (**3b**, **4b,** and **6b**) together with the known **1-2b** and **7b** gold complexes as well as silver complexes **1-2a**. Gold(I) complexes were investigated by means of NMR, mass spectroscopy, elemental analysis, and X-ray crystallographic studies. Accurate screening of co-catalysts and solvents led to identifying the best reaction conditions and the most active catalyst (**2b**) in the model hydroamination of phenylacetylene with aniline. Complex **2b** was then tested in the hydroamination of alkynes with a wide variety of arylamines yielding a lower percentage of product when arylamines with both electron-withdrawing and electron-donating substituents were involved. Computational studies on the rate-determining step of hydroamination were conducted to shed light on the significantly different yields observed when reacting arylamines with different substituents.

## 1 Introduction

Nowadays, considerable chemical research is aimed at developing greener strategies for synthesizing value-added compounds from low-cost reagents and using environmentally sustainable processes. The hydroamination reaction is an excellent example of this focus (Müller et al., 2008). This reaction, able to build C-N bonds by adding amines to multiple carbon bonds, can be eulogized as a model of a modern sustainable catalytically promoted chemical process with a 100% atom economy. The production of N-containing compounds (amines, imines, enamines, etc.) represents an important branch of the pharmaceutical and chemical industries, due to the importance of these molecules as scaffolds in the synthesis of biologically active compounds, drugs, N-heterocycles, polymers, bulk, and fine chemicals (Pohlki and Doye, 2003; Severin and Doye, 2007; Hartwig, 2008; Müller et al., 2008; Huang et al., 2015; Patel et al., 2017; Huo et al., 2019).

**SCHEME 1:**
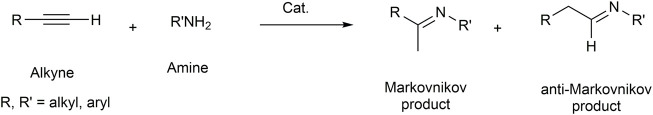
General intermolecular hydroamination of terminal alkyne.

The amine addition to double and triple carbon bonds requires very high activation barriers for the repulsion between electron-rich species (Müller et al., 2008). Nevertheless, metal complexes can decrease these barriers by coordinating one or more nucleophilic species (Scheme 1).

Many systems based on metal compounds are able to catalyze the reactions of alkaline and alkaline-earth metals (Jaspers and Doye, 2011; Mukherjee et al., 2011; Wixey and Ward, 2011), rare earth metals and actinides (Reznichenko et al., 2010b; 2010a), and early (groups 4 and 5) (Shi et al., 2001; Odom, 2005; Manna et al., 2011) and late transition (groups 8–12) (Hesp et al., 2010; Hesp and Stradiotto, 2010; Liu et al., 2018) metals. The latter, being less oxophilic and therefore more tolerant to air and moisture, are generally chosen for the hydroamination of alkynes to give enamines or imines, although high temperatures, long reaction times, and significant loads of catalysts are often required to achieve good conversions (Sevov et al., 2014; Gurak et al., 2016; Yang et al., 2017; Baron et al., 2018; Yahata et al., 2020).

The metal plays the important role of activating the double (or triple) C-C bond or the amine. As for alkaline and alkaline-earth metals, rare earth metals, and early transition metals, activation of the amine has been hypothesized. The catalytic cycle would involve the insertion of multiple C-C bonds into an M-N bond followed by fast protonolysis by other amino substrates (Michael et al., 2003; Reznichenko et al., 2010b; 2010a; Wixey and Ward, 2011).

On the other hand, two possible mechanisms have been proposed in the presence of late transition metals (E. Müller et al., 1999; Shanbhag and Halligudi, 2004; Katari et al., 2012): 1) the activation of the olefin (alkene or alkyne) would occur through its coordination to the metal, followed by the nucleophilic attack by the amine (Zhang et al., 2006); and 2) activation of the amine through oxidative addition to the metal center would be followed by the insertion of an unsaturated C–C bond into the M–N bond, where reductive elimination of the obtained intermediate would generate the hydroamination product and restore the catalyst (Zhao et al., 2005; Tsipis and Kefalidis, 2006).

Due to their carbophilicity and Lewis acidity, gold(I) and gold (III) complexes have recently received increased attention and have been used in homogeneous phase catalytic reactions to activate multiple C-C bonds that could undergo nucleophilic attack (Campeau et al., 2021). The most studied reactions are those that lead to the synthesis of nitrogen-containing heterocycles, the hydration (Biasiolo et al., 2015; Gatto et al., 2016; 2018b), alkoxylation (Gatto et al., 2018a), or hydroarylation (Tubaro et al., 2013; Baron and Biffis, 2019; Leung et al., 2020; Biffis et al., 2021) reactions of alkynes and the A^3^ (alkyne, aldehyde, and amine) coupling reactions (Visbal et al., 2018). Gold complexes can also catalyze the addition of an amine to an alkyne (Duan et al., 2009; Liu and Che, 2009; Alvarado et al., 2012; Sarcher et al., 2012; Gonell et al., 2013). Pioneering studies by Tanaka and coworkers reported the catalytic activity of (PPh_3_)AuCH_3_ in adding aniline to internal and terminal alkynes (Mizushima et al., 2003).

**FIGURE 1:**
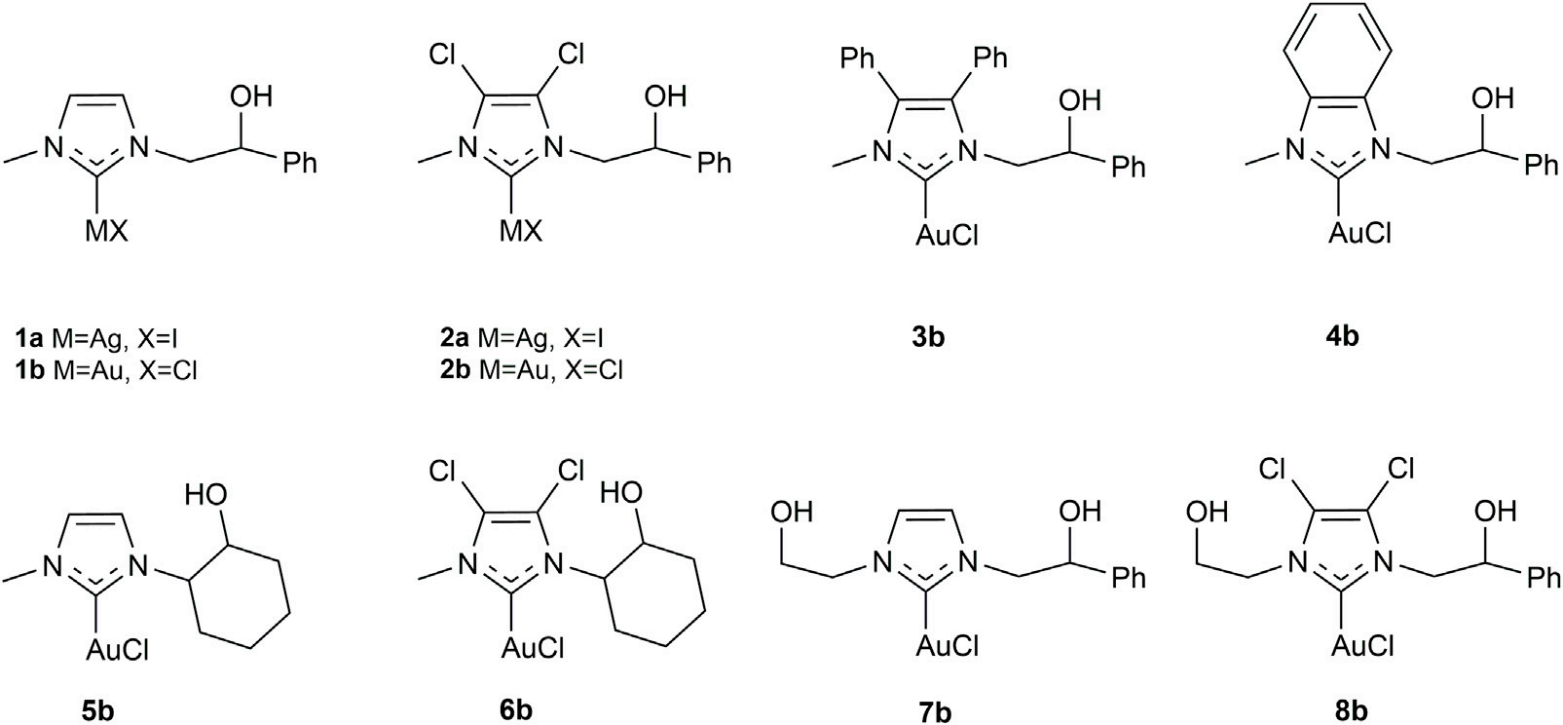
N-Heterocyclic carbene silver(I) and gold(I) complexes tested in the hydroamination reaction of phenylacetylene.

In the last 30 years, since the first isolation of free carbene by Arduengo (Arduengo et al., 1991), N-heterocyclic carbenes (NHCs) have frequently replaced phosphines for their ability to coordinate and stabilize transition metals as strong σ and π-donors and π-acceptors and their easily tunable electronic and steric properties (Jacobsen et al., 2009; Hopkinson et al., 2014; Nolan, 2014). NHC gold complexes have been extensively used to promote the hydroamination of alkynes (Mariconda et al., 2022). Recently, Bertrand et al. compared gold complexes bearing a variety of phosphine and carbene ligands, highlighting the superior performances of the latter (Yazdani et al., 2020). Herein we report a comparison among the activities of complexes **1a-b**, **2a-b**, and **3-8b** (Figure 1) with the addition of aniline to alkynes. Complexes **3b**, **4b**, and **6b** are described here for the first time, while all other complexes have already been reported in the literature (Napoli et al., 2013; Saturnino et al., 2016; Mariconda et al., 2020; Sirignano et al., 2021). Complexes **1**, **2**, **3b**, and **4b** differ in the substituents on the backbone of the NHC, while for complexes **5b**–**6b** and **7b**–**8b**, the authors have decided to study two effects: substituents on the backbone and modification of a substituent on a nitrogen atom. The choice of different substituents influences the electronic properties of the NHC, as reflected in the catalytic activity. In addition, the presence of the pendant hydroxyl group could be used as an advantageous linker functionality for immobilizing catalysts onto solid supports. Complex **2b**, which was found to be the best performing, was tested in the hydroamination of phenylacetylene, diphenylacetylene, and 4-octyne with a large variety of arylamines. Density Functional Theory (DFT) studies were conducted to understand the dramatical yield differences observed when reacting variously substituted arylamines.

## 2 Results and discussion

### 2.1 Synthesis and characterizations

**SCHEME 2:**
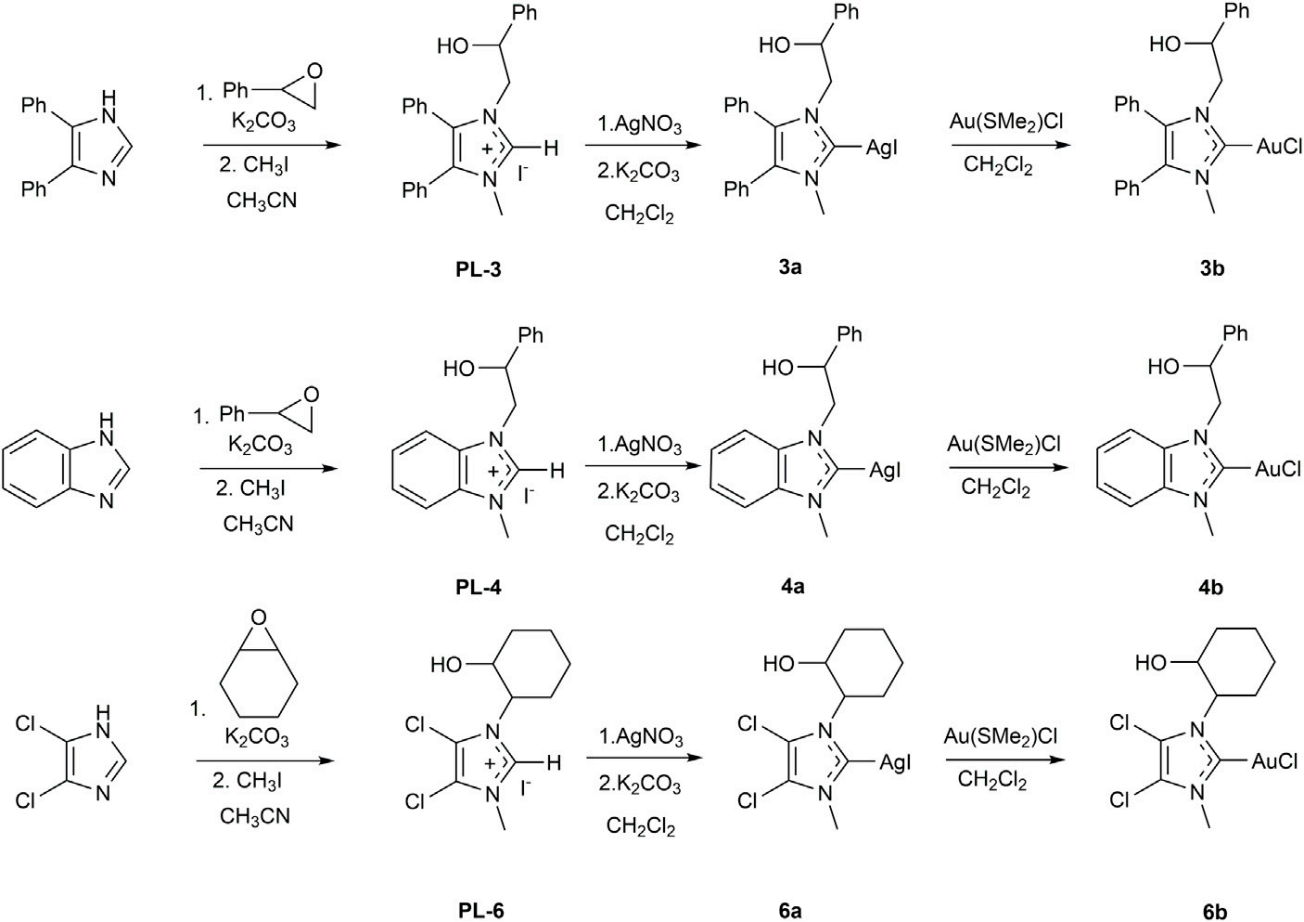
Synthetic routes for the synthesis of NHC-silver(I) (**3a**, **4a**, **6a**) and -gold(I) (**3b**, **4b**, **6b**) complexes.

The synthesis of NHC metal complexes were conducted following the procedure reported in the literature: **1a** (Napoli et al., 2013), **1b** and **5b** (Saturnino et al., 2016), **2a** and **2b** (Mariconda et al., 2020), and **7b** and **8b** (Sirignano et al., 2021). The synthetic routes used for the preparation of imidazolium salts and their relative silver(I) and gold(I) complexes are illustrated in Scheme 2. The NHC proligands were obtained following the procedure reported by Tacke (Patil et al., 2010) and modified for our purposes (Napoli et al., 2013; Saturnino et al., 2016; Mariconda et al., 2020; Sirignano et al., 2021). The sp^3^ hybridized nitrogen atom of imidazole-derivative was deprotonated by potassium carbonate to produce the nucleophilic species. Next, it was reacted with styrene oxide or cyclohexene oxide to give the monoalkylated product, to which an excess of iodomethane was added, to lead to the alkylation of the sp^2^-hybridized nitrogen atom and the formation of imidazolium salts (PL-3, PL-4, and PL-6).

All the new-synthesized proligands and their relative silver(I) and gold(I) complexes were characterized by nuclear magnetic resonance (^1^H- and^13^C-NMR), mass spectrometry (ESI or MALDI), and elemental analysis (see Materials and Methods). Moreover, crystals of **4b** suitable for X-ray diffractometry were also grown. In ^1^H-NMR spectra, the acid protons of imidazolium salts PL-3, PL-4, and PL-6 show signals at 9.50, 9.77, and 9.72 ppm, respectively, while in ^13^C-NMR spectra, signals at 138.88, 144.00, and 135.67 ppm are due to the carbocationic carbons (N*C*HN). The attribution of signals in the ^13^C-NMR spectra was supported by DEPT 135 experiments (see Supplementary Material).

MALDI-MS analyses present the signal attributable to the cationic portion of imidazolium salts.

The NHC silver(I) complexes were obtained by reacting the corresponding proligand with silver nitrate, in the presence of K_2_CO_3_, following the procedure published by Nolan and Gimeno (Collado et al., 2013; Visbal et al., 2013). The absence of the singlet signal of the acid proton in ^1^H-NMR spectra demonstrates the deprotonation and the consequent formation of carbene species, while the formation of the silver complexes is confirmed by ^13^C-NMR spectroscopy. In fact, the signals at 181.3_8_, 190.9_1_, and 182.9_0_ ppm of carbene carbons of **3a**, **4a**, and **6a**, respectively, were observed. MS analyses of three silver complexes indicate several signals of a bis-carbene structure [Ag(NHC)_2_]^+^. The presence of double signals for **3a** and **4a** is due to the silver isotopes ^107^Ag and ^109^Ag, almost equally abundant. Due to isotopes of chlorine (^35^Cl 75%, ^37^Cl 25%), the spectrum of **6a** is more intricate.

**SCHEME 3:**
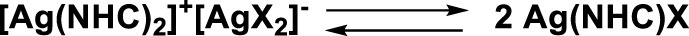
Possible dynamic equilibrium between bis and mono NHC-Ag(I) complex.

The presence bis-carbenic structures of Ag-complexes is well established in the literature, as also observed by solid state analysis (Mariconda et al., 2014). On the other hand, it is also well accepted that bis-carbenic species are in fast equilibrium with NHC-Ag(I)X complexes (Scheme 3).

This equilibrium is mainly influenced by the nature of the counterion (Lin and Vasam, 2007; Hintermair et al., 2011) as well as by the steric and donating properties of the N-heterocyclic carbene (Garrison and Youngs, 2005).

NHC-Au(I) complexes were synthesized by reaction among NHC-Ag(I) (**3a**, **4a**, and **6a**) complexes and chloro-gold(I)dimethyl-sulfide [Au(SMe_2_)Cl]. The gold complexes were obtained as yellow powder in good yield: 70% for **3b**, 75% for **4b**, and 78% for **6b**, respectively. ^1^H-NMR spectra of gold(I) complexes exhibited all the expected signals, corresponding to NHC-silver complexes. However, some differences were observed in the ^13^C-NMR spectra (see Supporting Information). As for ^13^C-NMR, carbene carbons of **3b**, **4b**, and **6b** were related to signals at 169.4_6_, 177.4_1_, and 171.2_0_ ppm, respectively. The up-field shift of the carbene carbon signals is due to the different nature of the metal center as well as the different electronic properties of counterion bound to the metal center (iodide for silver vs*.* chloride for gold) (Frenking et al., 1997; 2005; Baker et al., 2006). The mass spectra of gold complexes show peaks at 905.31858 for **3b**, 701.22194 for **4b**, and 693.06319 for **6b** m/z, confirming the presence of bis-carbene structures. All the complexes are stable in moisture and light. In fact, the spectra of all complexes in DMSO/D_2_O (90/10) stay unchanged after 24 h also when exposed to light.

## 3 X-ray crystallography

**FIGURE 2:**
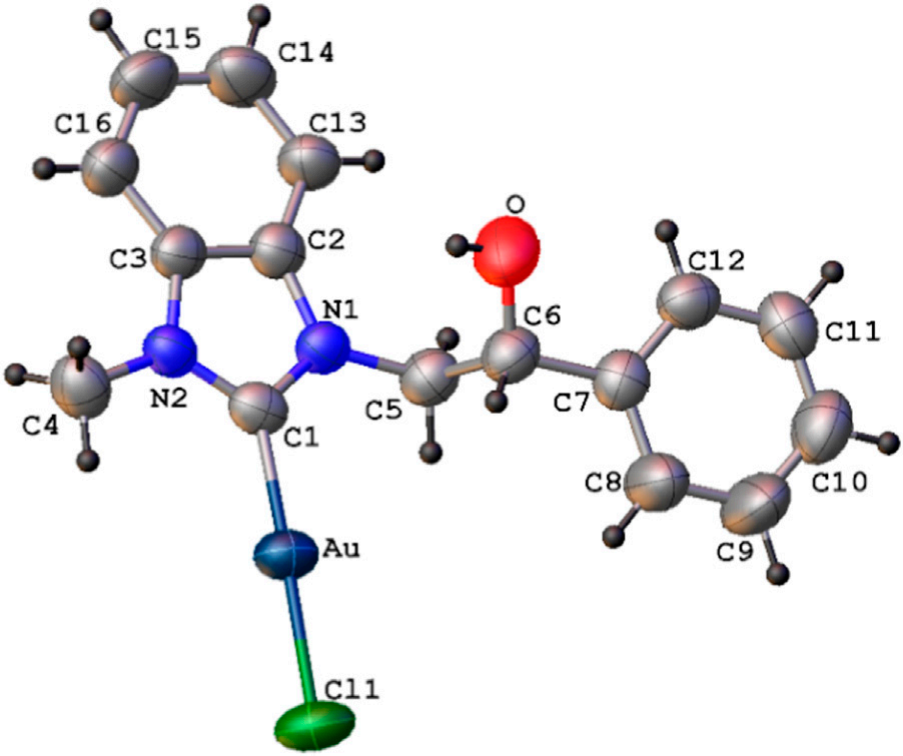
ORTEP diagram (thermal ellipsoids drawn at 50% probability) of complex **4b** with atomic numbering scheme (labels of hydrogen atoms omitted for clarity).

The X-ray single crystal molecular structure of complex **4b**, with the atomic numbering scheme, is shown in Figure 2. Selected bond distances and angles are shown in Table 1.

**TABLE 1:** Details of data collection and structure refinements for complex **4b**.

	Complex 4b
Empirical formula	C_16_H_16_AuClN_2_O
Formula weight	484.72
Crystal system	Monoclinic
Space group	P2_1_/c
T (K)	296 (2)
Radiation	Mo Kα (0.7107)
*a* (Å)	8.1963 (4)
*b* (Å)	12.5691 (6)
*c* (Å)	15.6845 (8)
α (deg)	90.00
β (deg)	94.868 (2)
γ (deg)	90.00
V (Å^3^)	1609.99 (14)
Z	4
ρ (g cm^-1^)	2.000
μ (mm^-1^)	9.303
θ range (°)	2.49–25.90
Reflections collected	31921
Unique data	3418
Observed reflections	2991
R_1_, I > 2σ(*I*)	0.0265
*w*R_2_, I > 2σ(*I*)	0.0725
Goodness of fit, *S*	1.012

Complex **4b**, found in the solid crystalline state in the neutral mono-carbenic form, presents a two-coordinate Au(I) atom in a nearly linear geometry, with Cl (1)-Au-C (1) bond angle of 178.3 (1)°. Both Au-C (1) and Au-Cl (1) bond lengths of 1.983 (5) and 2.289 (2) Å, respectively, are comparable with those already reported for similar Au(I)-carbene complexes (de Frémont et al., 2005; Hashmi et al., 2012; Zargaran et al., 2018; Costabile et al., 2021; Savchuk et al., 2022). Complex **4b** adopts a *trans* conformation around the C (5)-C (6) bond, as shown by the N (1)-C (5)-C (6)-C (7) torsion angle of 176.4 (4)°.

**FIGURE 3:**
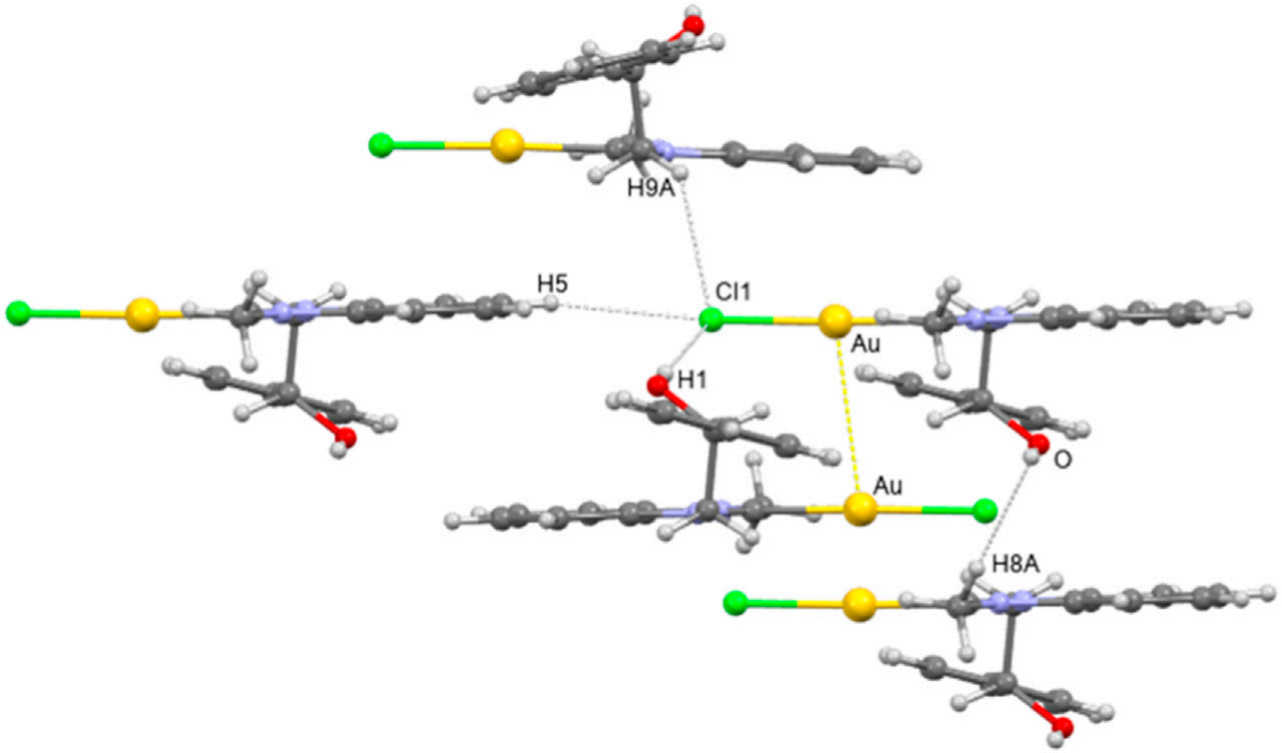
Crystal packing view of 4b showing the main intermolecular interactions.

The 3D packing of **4b** is characterized by the presence of dimers of discrete molecules, built up by aurophilic interactions with Au ^…^ Au contacts of 3.449 (5) Å (Figure 3).

Intermolecular hydrogen bonds between the–OH group and the chlorine atom of each molecule cooperate with the aurophilic interaction to the assembling of monomers (O ^…^ Cl (1)^*i*^ distance of 3.276 (1) Å and O-H ^…^ Cl (1) angle of 166.33°, *i* = -x+1, -y+1, -z). Each dimer is connected to the others mainly through C-H ^…^ Cl weak hydrogen bond interactions, involving both aromatic and a methylene hydrogen atom. Finally, C-H ^…^ O weak hydrogen bonds involving a methyl hydrogen atom are a further structural feature in the 3D crystal packing of **4b**.

## 4 Catalytic activity in hydroamination reactions

**SCHEME 4:**
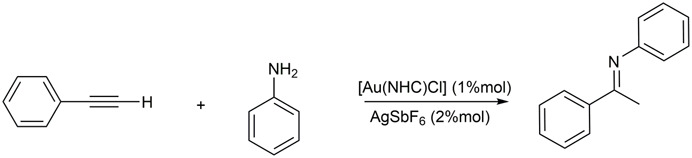
Hydroamination reaction of phenylacetylene with aniline.

NHC-Au(I) complexes, as shown in Figure 1, have been tested for the intermolecular hydroamination of phenylacetylene with aniline (Scheme 4), giving regioselective Markovinkov imine products (see Scheme 1).

**SCHEME 5:**
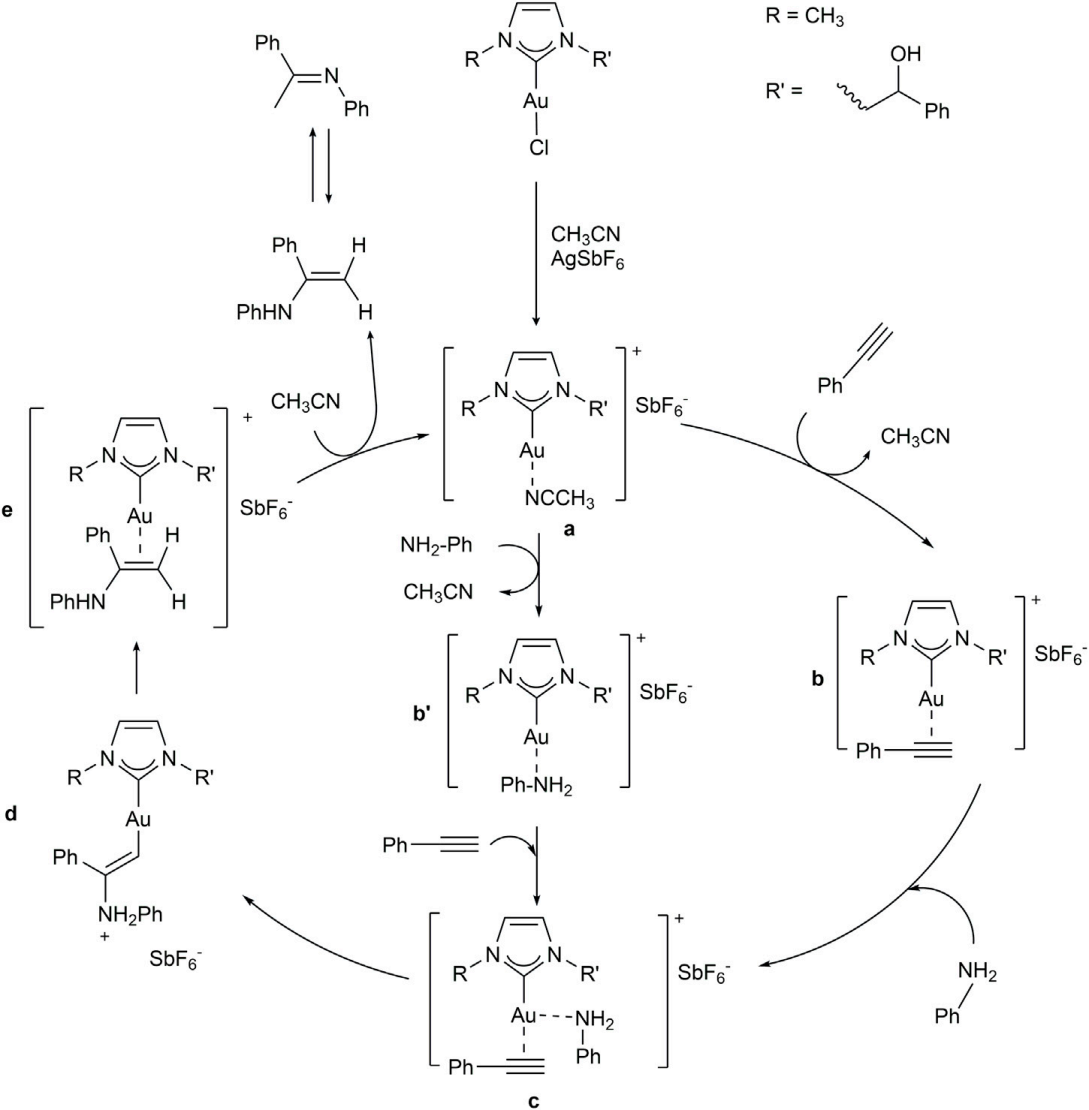
Proposed mechanism for the hydroamination reaction of phenylacetylene with aniline.

The reaction is co-catalyzed by two equivalents of silver salt in order to precipitate the chloride anion coordinated to the metal center and generate *in situ* the catalytic species **a** (Scheme 5). To select the best cocatalyst, different silver salts (hexafluoroantimonate, hexafluorophosphate, nitrate, and acetate) were tested using gold complex **7b** as catalytic precursor in the reaction between phenylacetylene and aniline. Screening of silver salts is reported in Table 2. The performances of the gold(I) complex were strongly influenced by the silver co-catalyst, and the best catalytic activities were found with non-coordinating anions such as hexafluoroantimonate (best performing) and hexafluorophosphate (Entries 1 and 2, Table 2). Low yields were obtained for reactions carried out in the presence of oxyanions (acetate and nitrate). Baron *et al.* (Baron et al., 2018) have reported similar results with different oxyanions (TsO^−^, TfO^−^). They asserted that the differences in catalytic activities could be the output of different reaction mechanisms or diverse rate-determining steps.

**TABLE 2:** Screening, promoted by 7b, of the silver salt co-catalyst for the hydroamination reaction of phenylacetylene with aniline.

Entry^a^	Co-catalyst	Yield (%)^b^
1	AgSbF_6_	36
2	AgPF_6_	29
3	AgNO_3_	15
4	AgOAc	n.d

^a^ Reaction conditions: 1.0 mmol aniline, 1.5 mmol phenylacetylene, 1% mol **7b**, 2% mol Ag salt, 90°C oil-bath, 16h, dry CH_3_CN (1 mL).

^b^ Yields are averaged from two runs and determined by 1H-NMR, analysis through internal standard.

An additional study conducted on the solvent identified acetonitrile as the most effective solvent in the hydroamination reaction using complex **2b**. Results are listed in Table 3 and agree with those reported in the literature (Dash et al., 2010; Nuevo et al., 2018; Kumar et al., 2020). The best catalytic performance in CH_3_CN (Entry 1, Table 3) is possibly caused by the coordination and stabilization of the catalytic species **a** (Scheme 5), after the abstraction of chloride anion. Indeed, Nolan and co-workers suggested that the use of a coordinating solvent can avoid the decomposition of gold(I) complexes to give colloidal gold (0) (de Frémont et al., 2009).

**TABLE 3:** Screening of the solvent in the reaction of hydroamination promoted by **2b**.

Entry^a^	Solvent	Yield (%)^b^
1	CH_3_CN	70
2	1,4-Dioxane	20
3	Dimethylsulfoxide	13
4	C_2_H_2_Cl_4_	45
5	Toluene	50
6	Ethylene Carbonate	35

^a^ Reaction conditions: 1.0 mmol aniline, 1.5 mmol phenylacetylene, 1% mol **2b**, 2% mol Ag salt, 90°C oil-bath, 16h, solvent (1 mL).

^b^ Yields are averaged from two runs and determined by ^1^H-NMR, analysis through internal standard.

Consequently, AgSbF_6_ co-catalyst and acetonitrile were chosen to compare the activity of gold complexes (**1-8b**), chloro-gold(I)dimethyl-sulfide, and silver complexes **1a** and **2a** in the hydroamination reaction of phenylacetylene with aniline.

As shown in Table 4, all complexes were able to promote hydroamination. Gold complexes (**1b** and **2b**) showed a higher catalytic activity than the silver analogues (compare entries 1 and 2 with entries 10 and 11, Table 4), highlighting the role of gold(I) complexes in this kind of reaction. The low catalytic activity of the chloro-gold(I)dimethyl-sulfide (Entry 11, Table 4) suggests the relevance of the NHC ligand on the stabilization and activation of the catalytic species. NHC-gold(I) complexes with chlorine atoms on the backbone of the ligand (**2b**, **6b** and **8b**) were more active than other gold(I) complexes. The better catalytic activity of these complexes could be caused by the ability of the chlorine atoms to reduce the σ-donor ability of the carbene, making the metal center more electrophilic. This would possibly allow faster coordination of the olefin to the metal at the early stages of the catalytic cycle (Mariconda et al., 2020; Sirignano et al., 2021) (see Scheme 5). An additional comment on the role of chlorine atoms can be found in the “Molecular modeling studies” section.

**TABLE 4:** Catalytic activity of NHC complexes in the hydroamination reaction of phenylacetylene with aniline.

Entry^a^	Catalyst	Yield (%)^b^
1	1b	55
2	2b	70
3	3b	53
4	4b	35
5	5b	30
6	6b	60
7	7b	34
8	8b	55
9	1a	10
10	2a	15
11	Au(SMe_2_)Cl	10
12	AgSbF_6_	16
13^c^	2b	99

^a^ Reaction conditions: 1.0 mmol aniline, 1.5 mmol phenylacetylene, 1% mol catalyst, 2% mol Ag salt, 90°C oil-bath, 16h, CH_3_CN (1 mL).

^b^ Yields are averaged of two runs and determined ^1^H-NMR, analysis through internal standard.

^c^ Run performed with 1% mol **2b**, 1% mol AgSbF_6_.

Once **2b** was identified as the most efficient complex, the catalyst/co-catalyst ratio was screened, identifying a 1:1 ratio as the optimal proportion. Lowering the co-catalyst to 1% mol caused an increase of the yield from 70% to 99% (compare Entry 13 with Entry 2, Table 4). Once the optimal conditions were identified, the hydroamination reaction scope was extended to a large variety of primary arylamines, keeping AgSbF_6_ as co-catalyst and acetonitrile as solvent.

As depicted in Table 5, all substrates generated the expected imines. Quantitative yields are obtained with aniline and methyl substituted anilines (entries 1–4, Table 5). Yields decrease when *iso*propyl substituted anilines and 2-naphthylamine are used (Entries 5–8, Table 5), as well as with 4-methoxyaniline (51%). Deactivating amines with electron-withdrawing groups on the aryl ring (entries 10, 11, 12 in Table 5) showed yields of 51 and 64% when substituted with halogens and only 20% yield when bearing the nitro group. Finally, the reaction between an internal alkyne (diphenylacetylene or 4-octyne) and aniline leads to a drastic reduction of the yield of the corresponding imine (15% and 5%, respectively).

**TABLE 5:** Substrate Screening in the hydroamination reaction with **2b**.

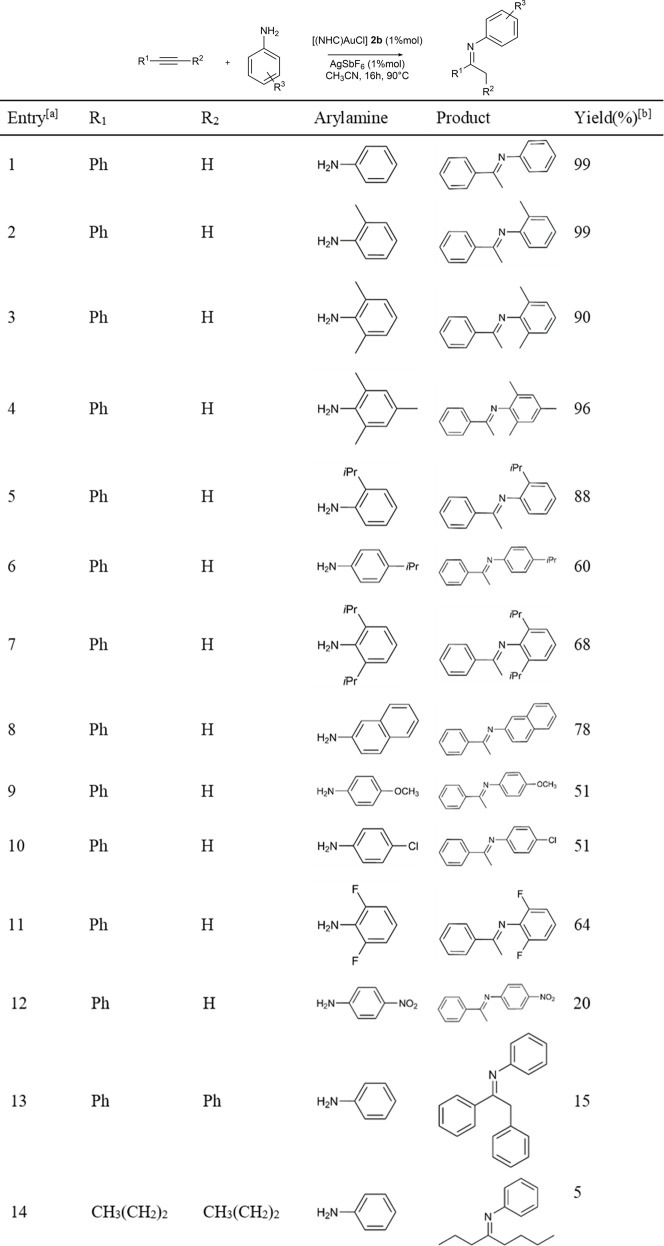

^a^Reaction conditions: 1.0 mmol aniline, 1.5 mmol phenylacetylene, 1% mol Au, 1% mol AgSbF_6_, 90°C oil-bath, 16h, dry CH_3_CN (1 mL).

^b^Yields are averaged of two runs and determined by ^1^H-NMR, analysis through internal standard.

## 5 Molecular modeling studies

According to experimental studies, when phenylacetylene reacts in the presence of catalyst **2b**, quantitative yield percentage was observed only with aniline and *orto*-methyl aniline (Table 5). All other substituted anilines gave lower percentage yield independently from the nature and position of the substitution. This means that electron-donating activating aryl substituents on aniline influence the kinetics of the reaction in the same direction as the electron-withdrawing activating substituents and the electron-withdrawing deactivating substituents. To investigate this intriguing outcome, DFT (Density Functional Theory) studies at the PBE0/6–311-G (d,p) level were conducted.

The hydroamination reaction mechanism in the presence of NHC-Au complexes was extensively studied by Ghosh et al. (Katari et al., 2012). In detail, Ghosh and co-workers investigated the hydroamination reaction between MeC≡CH and PhNH_2_, as representative substrates, in the presence of [1,3-dimethylimidazol-2-ylidene] gold chloride. In this study, the hydrogen transfer (reaction **d→e** of Scheme 5) from nitrogen to carbon enabling the formation of the enamine [(NHC)Au(PhNHMeC = CH_2_)]^+^ (**e**) from the intermediate [(NHC)AuCH = CMeNH_2_Ph]^+^ (**d**) was revealed to be the rate-determining step.

In detail, this proton transfer can be assisted by a water molecule or a PhNH_2_ substrate (Katari et al., 2012). As shown by the authors, the proton transfer assisted by a water molecule presents a slightly lower energy with respect to that assisted by PhNH_2_ substrate, occurs in only one step, and would possibly occur even in presence of only traces of water.

In the hydroamination reactions conducted in this work, we can assume the presence of traces of water since substrates employed were not dried before the reaction.

**SCHEME 6:**
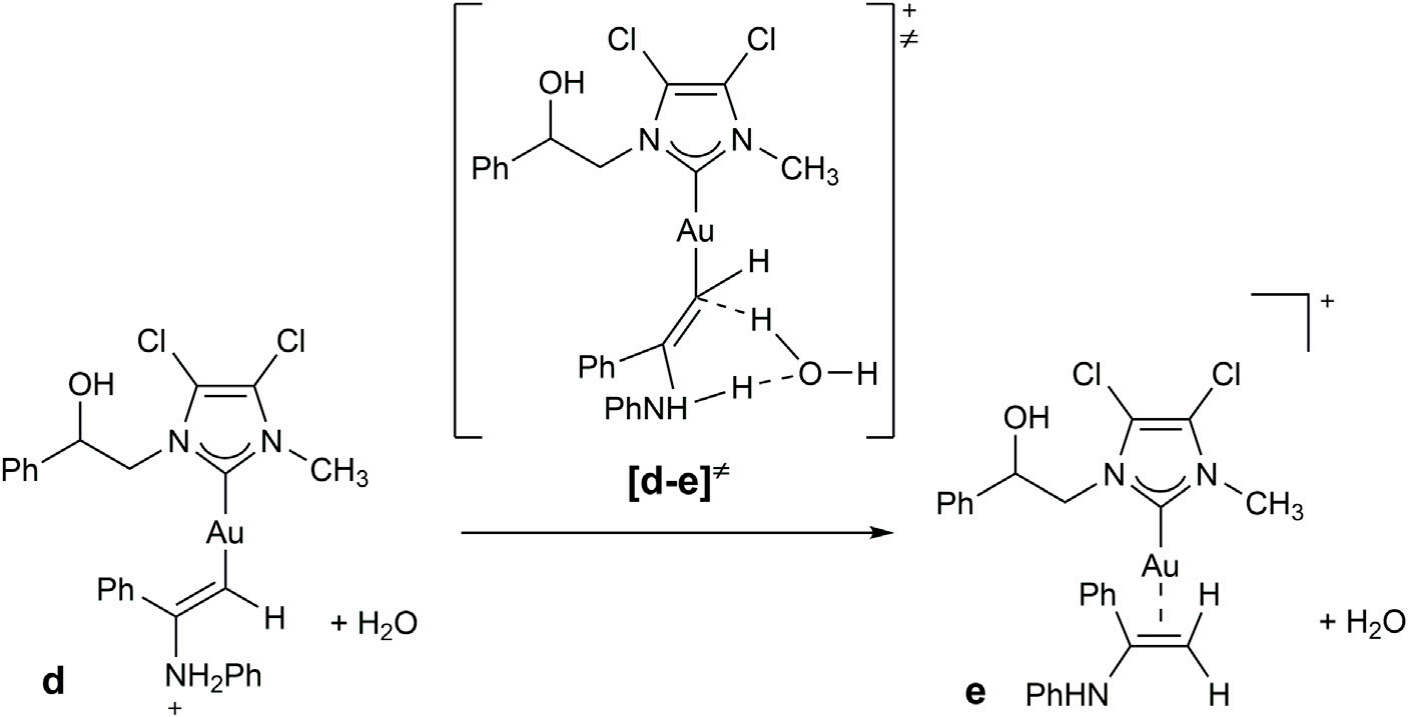
Proposed mechanism for the hydroamination reaction of phenylacetylene with aniline.

As a consequence, we investigated the proton transfer assisted by water from [(NHC)AuCH = CPhNH_2_Ph]^+^(**d**) to [(NHC)Au(PhNHPhC = CH_2_)]^+^(**e**) for selected substrates in the presence of **2b**. Substrates were chosen taking into account results of Table 5, in order to compare entries 1, 5, 10 and 12, involving phenylamines with different electron-withdrawing and donating groups and steric hindrance. Minimum energy intermediates (**d** in Scheme 6) and transition states (TS) of proton transfer ([**d-e]** ^**≠**^ of Scheme 6) were calculated and compared with the energy of the solvent coordinated initiating species [(NHC)AuCH_3_CN]^+^ (**a** in Scheme 5).

**FIGURE 4:**
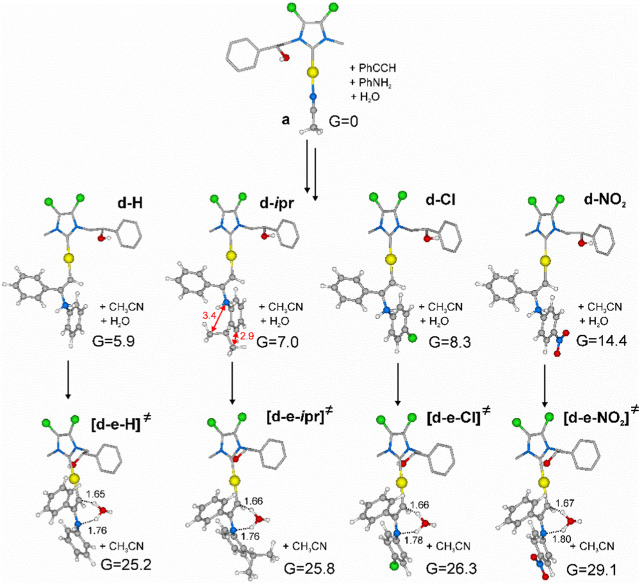
Minimum energy intermediates (**d-H**, **d-*i*pr**, **d-Cl**, **d-NO**_**2**_) and transition states relative to proton transfer (**[d-e-H]** ^**≠**^, **[d-e-*i*pr]** ^**≠**^, **[d-e-Cl]** ^**≠**^, **[d-e-NO**_**2**_ **]** ^**≠**^) located according to Scheme 6, starting from minimum energy active species **a** of Scheme 5. Free energies calculated at PBE0/6–311-G (d,p) in CH_3_CN are in kcal/mol. Distances are in Å. Some hydrogens of the NHC ligand skeleton were omitted for clarity.

Geometries and free energies calculated in acetonitrile have been reported in Figure 4.

According to molecular modeling results, intermediate **d-H** and TS **[d-e-H]** ^**≠**^, involving aniline, present higher energies with respect to those calculated by Ghosh and co-workers (Katari et al., 2012), possibly due to the higher steric hindrance of phenylacetylene with respect to propyne and to the higher electron-withdrawing NHC bearing chlorines on the backbone.

Shifting to a comparison among intermediates **d**, we can observe an increase of free energy for substituted phenylamines. As for **d-*i*pr**, the electro-donating ability of the isopropyl group does not compensate its steric hindrance, which introduces internal repulsions as reported in Figure 4. On the other hand, electron-withdrawing groups in *para* position, such as chlorine or -NO_2_, lead to intermediates with higher energy, possibly due to electron depletion of the metal.

The same energy trend was seen for hydrogen transfer TS ([**d-e]** ^**≠**^), although energy differences were reduced. This is possibly due to an increase of acidity of the hydrogen bound to the nitrogen, especially in the presence of electron-withdrawing groups.

To gain details on the electronic effects of the various substrates on the percentage yields, we calculated the Au charge for all intermediates and TS by carrying out a natural bond orbital (NBO) analysis. In Table 6, experimental percentage yield, intermediate and free energy barriers in gas phase and acetonitrile, and Au charges were collected. Electron depletion of the Au can be observed for amine with electron-withdrawing groups and could be responsible for the increase of energy barriers for these substrates.

**TABLE 6:** Comparison among percentage yield of hydroamination and calculated free energies and Au charges for intermediate d (d-H, d-*i*Pr, d-Cl, d-NO_2_) and TS [d-e]^≠^ ([d-e-H]^≠^, [d-e-*i*pr]^≠^, [d-e-Cl]^≠^, [d-e-NO_2_]^≠^) relative to water-assisted proton transfer according to Scheme 6.

Arylamine	% Yield	**d**	G (CH_3_CN) ^a^	Au charge	**[d-e]** ^≠^	G (CH_3_CN) ^a^	Au charge^b^
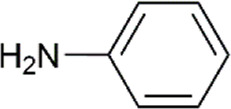	99	**d-H**	5.9	0.28529	**[d-e-H]** ^**≠**^	25.2	0.2871
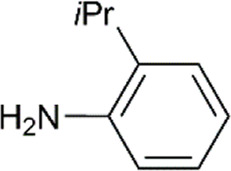	88	**d-*i*Pr**	7.0	0.28187	**[d-e-*i*pr]** ^**≠**^	25.8	0.2865
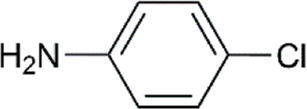	51	**d-Cl**	8.3	0.28478	**[d-e-Cl]** ^**≠**^	26.3	0.28967
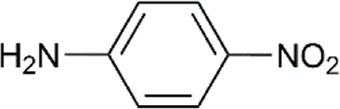	20	**d-NO**_**2**_	14.4	0.28689	**[d-e-NO**_**2**_ **]** ^**≠**^	29.1	0.29183

^a^ Free energies in kcal/mol are calculated with respect to intermediate active species **a**, according to Scheme 4.

^b^ Au charges were obtained from NBO, analysis.

On the other hand we can speculate that, besides the steric effects, the presence of electron-donating substituents increases the energy barrier due to a decrease of the amine acidity since electron-withdrawing substituents actually give lower energy barriers.

According to computational studies, the non-monotonic trend observed for the reactivity of substituted aniline, where a drop in the percentage yield has been observed with both electron-donating or withdrawing substituents, would be the balance of two contrasting effects: the stabilization of the intermediate preceding the rate-determining step barrier (favored for electron-donating substituted anilines) and the decrease of the barrier itself (favored for electron-withdrawing substituted anilines due to their acidity). The global effect leads to the highest percentage yield for non-substituted aniline where these two factors find the best balance.

In this framework, the higher performances of catalysts with chlorine on the backbone could be rationalized supposing that electron-withdrawing substituents on NHC would increase the acidity of the ammonium intermediate **d** decreasing the overall **[d-e]** ^**≠**^ barrier and favoring the hydrogen transfer.

This study confirms, as previously reported by Ghosh (Katari et al., 2012), that the hydrogen transfer represents a key step in the alkyne hydroamination, and variables able to decrease the energy of this step can be responsible for the increase of percentage yields. It is important to underline that the hypothesis that the proton transfer from the amine to the carbon bonded to the metal is mediated by water is also supported by the fact that when conducting the reaction between aniline and phenylacetylene as in Entry 1, but in the presence of molecular sieves to eliminate traces of H_2_O, no product is obtained.

## 6 Conclusion

NHC-Au(I) complexes (**1-8b**) have been tested as catalysts in the hydroamination reaction of alkynes to give imines. New complexes **3b**, **4b**, and **6b** were characterized by NMR, mass spectroscopy, elemental analysis, and, as for **4b**, by X-ray diffraction analysis. All complexes were shown to be active in the hydroamination reaction of phenylacetylene. During the optimization of the reaction conditions, AgSbF_6_ was selected as the best co-catalyst, and acetonitrile as the best solvent. Complex **2b** was revealed as the most active catalyst and was chosen to study the different catalytic behaviors on varying the involved arylamine. The presence of the two chlorine atoms (electron-withdrawing) positively influences the catalytic activity. Quantitative yields were obtained only by reacting aniline and *o*-methyl aniline. All other substituted arylamines gave lower yields independently from the electron-donating or -withdrawing ability of the substituents. This non-monotonic reactivity was investigated by DFT studies. According to theoretical results, the low reaction yields in the presence of electron-withdrawing groups is caused by the destabilization of the intermediate [(NHC)AuCH = CPhNH_2_Ph]^+^ preceding the water-assisted hydrogen transfer from nitrogen to carbon as consequence of electron depletion of the metal induced by electron-withdrawing groups on *N*-phenyl group of intermediate. On the other hand, the low reaction yield observed with electron-donating arylamine substituents would be the consequence of a decrease of acidity of the ammonium group of intermediate [(NHC)AuCH = CPhNH_2_Ph]^+^ that could increase the TS barrier of the hydrogen transfer. In the framework, aniline and *o*-methyl aniline perfectly balance these two opposite effects, giving the best yields.

## 7 Materials and methods

### 7.1 General methods

All the reactions were carried out using Schlenk and glove-box techniques, under a dry nitrogen atmosphere. Solvents were dehydrated by heating at reflux temperature over suitable drying agents. Reagents were purchased from Merck KGaA (Darmstadt, Germany) and TCI Chemicals (Tokyo, Japan), and they were used as received. NMR spectra were recorded on Brucker AM 300 spectrometers (300 MHz for ^1^H; 75 MHz for ^13^C) and Brucker AVANCE 400 spectrometer (400 MHz for ^1^H; 100 MHz for ^13^C) using DMSO-d_6_ and CDCl_3_ as solvents. The chemical shifts are referenced to tetramethylsilane (SiMe_4_, *δ* = 0) by using residual protons impurities of deuterated solvents as internal standards. The multiplicities of spectra are abbreviated in the following manner: singlet (s), doublet (d), triplet (t), multiplet (m), broad (br), and overlapped (o). Elemental analyses for C, H, and N were recorded with Thermo-Finnigan Flash EA 1112 following microanalytical procedures. Chloride and iodide were determined by the reaction of AgNO_3_ with halogen, precipitation of AgX (X = Cl, I), which was dissolved in Na_2_S_2_O_3_. The content of silver was determined by flame atomic absorption spectroscopy (FAAS), and halogen content was calculated by using the content of silver.

ESI-MS measurements of organic compounds were acquired on a Waters Quattro Micro triple quadrupole mass spectrometer equipped with an electrospray ion source. MALDI-MS was performed using a Brucker SolariX XR Fourier transform ion cyclotron resonance mass spectrometer (Brucker DaltonikGmBH, Bremen, Germany) equipped with a 7T refrigerated actively shielded superconducting magnet (Brucker Biospin, Wissembourg, France). The mass range is set to m/z 200–3000. To improve the mass accuracy, the sample spectra were calibrated internally by matrix ionization (2,5-dihydroxybenzoic acid). Single crystal X ray diffraction data of complex **4b** were collected at room temperature with a Bruker-Nonius X8APEXII CCD area detector system equipped with a graphite monochromator with radiation Mo Kα (*λ* = 0.71073 Å). Data were processed through the SAINT (SAINT, 2023) reduction and SADABS (Sheldrick, 2003) absorption software. The structure was solved by direct methods and refined by full matrix least-squares based on *F* ^2^ through the SHELX and SHELXTL-2018 structure determination package (Sheldrick, 2008; Sheldrick, 2015). All non-hydrogen atoms were refined anisotropically, and hydrogen atoms were included as idealized riding atoms. All graphical representations were obtained by using Olex2 (Dolomanov et al., 2009) and CCDC Mercury 4.0 (Macrae et al., 2020). Details of data and structure refinements are reported in Table 1. The supplementary crystallographic data were deposited as CCDC 2260506.

### 7.2 Synthesis and characterization

#### 7.2.1 General procedure for synthesis of N-heterocyclic carbene proligands (PL-3, PL-4, PL-6)

Proligands PL-3, PL-4, and P-L6 were synthesized following the strategy developed by Tacke (Patil et al., 2010) and employing the procedures reported in the literature (Napoli et al., 2013; Mariconda et al., 2014; 2020; Saturnino et al., 2016). Imidazole ring (1 eq) was dissolved in CH_3_CN and deprotonated by K_2_CO_3_ (2 eq), then it was reacted with (1.2 eq) epoxy-alkylating agent (styrene oxide or cyclohexene oxide) for 12 h at refluxing temperature. Afterwards, the reaction mixture was filtered, and iodomethane (5 eq) was added. The reaction mixture was stirred for 8 h. The imidazolium salts were recovered by removing the solvent, and precipitation in acetone. Finally, the product was washed with hexane (3 × 30 mL) and diethyl ether (2 × 30 mL).

Iodo [4,5-diphenyl N-methyl, N’-(2-hydroxy-2-phenyl) ethyl-imidazole-2-ylidine] PL-3.

Yield: 1.92 g; 88%

^**1**^ **H-NMR** (400 MHz, DMSO-d_6_): *δ* 9.50 (s, 1H, NC ***H*** N); 7.46–7.11 (m, 15H, ***Ph rings***); 6.12 (s, 1H, O ***H***); 4.72 (m, 1H, OC ***H***, *J*_*anti*_ *7.37 Hz, J*_*gauche*_ *5.80 Hz*); 4.26–4.11 (m, 2H, NC ***H***_***2,***_ *J*_*gem*_ *14.5 Hz, J*_*anti*_ *7.37 Hz, J*_*gauche*_ *5.80 Hz*); 3.84 (s, 3H, NC ***H***_***3***_).

^**13**^ **C-NMR** (100 MHz, DMSO-d_6_): *δ* 140.7 *(ipso aromatic carbon*, ***Ph ring***); 136.8 (N ***C*** N); 131.3–125.5 (*aromatic carbons*, ***Ph rings***), 125.5–125.0 (*backbone carbons*, N ***C*** Ph = ***C*** PhN), 70.0 (O ***C*** H); 54.0 (N ***C*** H_2_); 34.4 (N ***C*** H_3_).

**MALDI-ToF (m/z):** 355.18080 Da attributable to the cationic portion of the imidazolium salt [C_24_H_23_N_2_O]^+^.

***Elemental Analysis:*** calculated for C_24_H_23_I N_2_O, C 59.76, H 4.81, I 26.31, N 5.81, O 3.32; Found: C 59.60, H 4.70, I 26.58, N 5.60, O. 3.43.

Iodo [N-methyl, N’-(2-hydroxy-2-phenyl) ethyl-benzoimidazol-2-ylidine] PL-4.

Yield: 2.85 g; 85%

^**1**^ **H-**NMR (400 MHz, DMSO-d_6_): *δ* 9.77 (s, 1H, NC ***H*** N); 8.06–7.31 (m, 9H, ***Ph rings***); 5.99 (d, 1H, O ***H***); 5.10–5.07 (dd, 1H, OC ***H*** *, J*_*anti*_ *7.58 Hz, J*_*gauche*_ *5.50 Hz*); 4.61–4.52 (m, 2H, NC ***H***_***2*,**_ *J*_*gem*_ *14.7 Hz, J*_*anti*_ *7.58 Hz, J*_*gauche*_ *5.50 Hz*); 4.14 (s, 3H, NC ***H***_***3***_).

^**13**^ **C-NMR** (100 MHz, DMSO-d_6_): *δ* 144.0 (N ***C*** N); 141.9 (*ipso aromatic carbon*, ***Ph ring***); 131.6–131.3 (*backbone carbons*, N ***C*** = ***C*** N); 128.3, 127.9, 126.3, 126.1 (*aromatic carbons*, ***Ph rings***); 114.0, 113.4 (*aromatic carbons*, ***Ph rings***); 70.0 (O ***C*** H); 54.0 (N ***C*** H_2_), 34.4 (N ***C*** H_3_).

**MALDI-ToF** (m/z): 253.13474 Da attributable to the cationic portion of the imidazolium salt [C_16_H_17_N_2_O]^+^.

**Elemental Analysis*:*** calculated for C_16_H_17_I N_2_O, C 50.44, H 4.10, I 33.38, N 7.37, O 4.41; Found: C 50.50, H 4.50, I 33.20, N 7.40, O. 4.40.

Iodo [4,5-dichloro l N-methyl, N’-(cyclohexane-2-ol) imidazole-2-ylidine] PL-6.

Yield: 1.88 g; 70%

^**1**^ **H-NMR** (400MHz, DMSO-d_6_): *δ* 9.72 (s, 1H, NC ***H*** N); 5.25 (m, 1H, O ***H***); 4.09 (m, 1H, OC ***H,*** *J*_*ax-eq*_ *4.81 Hz, J*_*ax-eq*_ *4.50 Hz, J*_*eq-eq*_ *2.8Hz*); 3.84 (s, 3H, NC ***H***_***3***_); 3.70 (m, 1H, NC ***H,*** *J*_*ax-ax*_ *11.5 Hz, J*_*ax-eq*_ *4.81 Hz, J*_*eq-eq*_ *2.7 Hz*); 2.08–1.36 (m, 8H, ***Cyclohexyl protons***).

^**13**^ **C-NMR** (100 MHz, DMSO-d_6_): *δ* 135.6 (N ***C*** N); 118.8 (*backbone carbons*, N ***C*** Cl = ***C*** ClN); 71.5 (O ***C*** H); 65.2 (N ***C*** H); 35.2 (N ***C*** H_3_); 34.0, 30.68, 24.2, 23.5 (***Cyclohexyl carbons***).

**MALDI-ToF (m/z):** 249.05614 Da attributable to the cationic portion of the imidazolium salt [C_10_H_15_Cl_2_N_2_O]^+^.

**Elemental Analysis*:*** calculated for C_10_H_15_Cl_2_IN_2_O, C 31.86, H 4.01, Cl 18.80, I 33.60, N 7.43, O 4.24; Found: C 31.70, H 4.00, Cl 18.96, I 33.50, N 7.48, O. 4.29.

#### 7.2.2 General procedure for synthesis of Ag-NHC complexes (3a, 4a, 6a)

NHC-Ag(I) complexes were synthesized using the procedure published in literature by Nolan and Gimeno (Collado et al., 2013; Visbal et al., 2013) and slightly modified by us. The imidazolium salt (1 eq) and AgNO_3_ (1 eq) were suspended in dichloromethane (25 mL) and stirred for 2 h. Then, K_2_CO_3_ (10 eq) was added, and the resulting mixture was stirred for 6 h with the exclusion of light. Then, the reaction mixture was filtered through Celite, and the complex was obtained removing the solvent *in vacuo*.

Iodo [4,5-diphenyl N-methyl, N’-(2-hydroxy-2-phenyl) ethyl-imidazole-2-yden]silver(I) 3a.

Yield: 0.412 g, 70%

^**1**^ **H-NMR** (400 MHz, DMSO-d_6_): *δ* 7.46–7.04 (m, 15H, ***Ph rings***); 4.76 (m, 1H, OC ***H,*** *J*_*anti*_ *7.73 Hz, J*_*gauche*_ *5.00 Hz*); 4.19 (m, 2H, NC ***H***_***2***_ ***,*** *J*_*gem*_ *14.0 Hz, J*_*anti*_ *7.73 Hz, J*_*gauche*_ *5.00 Hz*); 3.82 (s, 3H, NC ***H***_***3***_).

^**13**^ **C-NMR** (100 MHz, DMSO-d_6_): *δ* 181.4 (N ***C*** N); 142.2 *(ipso aromatic carbon*, ***Ph ring***); 131.9–125.6 (*aromatic carbons*, ***Ph rings***); 72.2 (O ***C*** H); 56.0 (N ***C*** H_2_); 37.4 (N ***C*** H_3_).

**ESI-MS (m/z):** 647.45829 Da attributable to a bis-carbene structure [C_35_H_30_AgN_4_O_2_]^+^.

**Elemental Analysis:** calculated for C_24_H_22_AgIN_2_O, C, 48.92; H, 3.76; Ag, 18.31; I, 21.54; N, 4.75; O, 2.72; Found: C 48.60, H 3.70, Ag 18.30, I 21.84, N 5.66, O 2.72.

Iodo [N-methyl, N’-(2-hydroxy-2-phenyl) ethyl-benzoimidazol-2-yden]silver(I) 4a.

Yield: 0.267 g, 55%

^**1**^ **H-NMR** (400 MHz, DMSO-d_6_): *δ* 7.82–7.24 (m, 9H, ***Ph rings***); 5.86 (s, 1H, O ***H***); 5.09–5.07 (dd, 1H, OC ***H,*** *J*_*anti*_ *7.85 Hz, J*_*gauche*_ *5.40 Hz*); 4.65 (m, 2H, NC ***H***_***2,***_ *J*_*gem*_ *13.83 Hz, J*_*anti*_ *7.85 Hz, J*_*gauche*_ *5.40 Hz*); 4.04 (s, 3H, NC ***H***_***3***_).

^**13**^ **C-NMR** (100 MHz, DMSO-d_6_): *δ* 190.9 (N ***C*** N); 142.3 *(ipso aromatic carbon*, ***Ph-ring***); 134.4, 133.9 (*backbone carbons*, N ***C*** = ***C*** N); 128.2, 127.5, 126.2, 123.6 (*aromatic carbons*, ***Ph-rings***); 112.6, 111.7 (*aromatic carbons*, ***Ph rings***); 72.0 (O ***C*** H); 55.8 (N ***C*** H_2_); 35.5 (N ***C*** H_3_).

**MALDI-ToF (m/z):** 517.26179 Da attributable to bis-carbene structure [C_26_H_25_AgN_4_O]^+^.

**Elemental Analysis:** calculated for C_16_H_16_IN_2_O, C 39.45, H 3.31, Ag 22.15, I 26.05, N 5.75, O 3.28; Found: C 39.00, H 3.30, Ag 22.00, I 26.50, N 5.60, O 3.43.

Iodo [4,5-dichloro N-methyl, N’-(cyclohexane-2-ol) imidazole-2-yden] silver (I) 6a.

Yield: 0.310 g, 64%

^**1**^ **H-NMR** (400 MHz, DMSO-d_6_): *δ* 4.93 (b, 1H, O ***H***); 4.00 (m, 1H, HOC ***H*** *J*_*ax-eq*_ *4.81 Hz, J*_*ax-eq*_ *4.50 Hz, J*_*eq-eq*_ *2.8Hz*); 3.86 (o, 4H, NC ***H***, NC ***H***_***3***_); 1.96–1.31 (m, 8H, ***Cyclohexyl protons***).

^**13**^ **C-NMR** (100 MHz, DMSO-d_6_)**:** *δ* 182.9 (N ***C*** N); 118.6, 117.3 (***backbone carbons,*** N ***C*** Cl = ***C*** ClN); 76.1 (***C*** HOH); 70.4 (N ***C*** H); 38.8 (N ***C*** H_3_); 35.0, 30.7, 24.2, 23.9 (***Cyclohexyl carbons***).

**MALDI-ToF (m/z):** 606.99642 Da attributable to [C_20_H_28_AgCl_4_N_4_O_2_]^+^.

**Elemental Analysis:** calculated for C_10_H_14_AgCl_2_IN_2_O, C 24.82, H 2.92, Ag 22.29, Cl 14.65 I 26.22, N 5.79, O 3.31; Found: C 24.53, H 2.92, Ag 22.00, Cl 14.35, I 26.30, N 5.81, O 3.51.

#### 7.2.3 General procedure for synthesis of Au-NHC complexes (3b, 4b, 6b)

NHC-gold(I) complexes were prepared by the *trans*-metalation route, following the procedure published in the literature (Lin and Vasam, 2007). The suitable Ag-NHC complex (1.00 eq) was reacted with chloro (dimethylsulfide) gold(I) (1 eq) in dry CH_2_Cl_2_ (25 mL) for 4 h at room temperature. Afterward, the mixture was filtered, through Celite, to remove the AgI byproduct. NHCAu complex was obtained after removal of the solvent *in vacuo*.

Chloro [4,5-diphenyl N-methyl, N’-(2-hydroxy-2-phenyl) ethyl-imidazole-2-ylidine] gold(I) 3b

Yield: 0.16 g, 70%

^**1**^ **H-NMR** (400 MHz, DMSO-d_6_): *δ* 7.36–7.14 (m, 15H, ***Ph rings***); 5.13 (m, 1H, OC ***H,*** *J*_*anti*_ *7.00 Hz, J*_*gauche*_ *5.30 Hz*); 4.11 (m, 2H, NC ***H***_***2***_ ***,*** *J*_*gem*_ *13.89 Hz, J*_*anti*_ *7.00 Hz, J*_*gauche*_ *5.30Hz*); 3.72 (s, 3H, NC ***H***_***3***_).

^**13**^ **C-NMR** (100 MHz, DMSO-d_6_): *δ* 169.5 (N ***C*** N); 141.8 *(ipso aromatic carbon*, ***Ph ring***); 131.7–125.5 (*aromatic carbons*, ***Ph rings***); 72.3 (O ***C*** H); 55.6 (N ***C*** H_2_); 36.9 (N ***C*** H_3_).

**MALDI-ToF (m/z):** 905.31858 Da attributable to bis-carbene structure [C_48_H_44_AuN_4_O_2_]^+^.

**Elemental Analysis:** calculated for C_24_H_22_AuClN_2_O, C 49.12, H 3.78, Au 33.56, Cl 6.04, N 4.77, O 2.73; Found: C 49.00, H 3.70, Cl 6.10, N 4.70.

Chloro [N-methyl, N’-(2-hydroxy-2-phenyl) ethyl-benzimidazol-2-ylidine] gold(I) 4b

Yield: 0.150 g, 60%

^**1**^ **H-NMR** (400 MHz, DMSO-d_6_): *δ* 7.80–7.28 (m, 9H, ***Ph rings***); 5.76 (s, 1H, O ***H***); 5.22 (m, 1H, OC ***H,*** *J*_*anti*_ *7.68 Hz, J*_*gauche*_ *5.89 Hz*); 4.60 (m, 2H, NC ***H***_***2***_ ***,*** *J*_*gem*_ *14.65 Hz, J*_*anti*_ *7.68 Hz, J*_*gauche*_ *5.89Hz*); 4.00 (s, 3H, NC ***H***_***3***_).

^**13**^ **C-NMR** (100 MHz, DMSO-d_6_): *δ* 177.4 (N ***C*** N); 141.9 *(ipso aromatic carbon*, ***Ph ring***); 133.5, 133.1 (*backbone carbons*, N ***C*** = CN); 128.3, 127.7, 126.0, 124.1 (*aromatic carbons*, ***Ph rings***); 112.8, 111.8 (*aromatic carbons*, ***Ph rings***); 72.5 (O ***C*** H); 55.4 (N ***C*** H_2_); 35.0 (N ***C*** H_3_).

**MALDI-ToF (m/z):** 701.22194 Da attributable to bis-carbene structure [C_32_H_32_AuN_4_O_2_]^+^.

**Elemental Analysis:** calculated for C_16_H_16_AuClN_2_O, C 39.65, H 3.33, Au 40.63, Cl 7.31, N 5.78, O 3.30; Found: C 40.00, H 3.00, Cl 7.41, N 5.80.

Chloro [4,5-dichloro N-methyl, N’-(2-hydroxy-2-phenyl) ethyl-benzimidazol-2-ylidine] gold(I) 6b

Yield: 0.220 g, 90%.

^**1**^ **H-NMR** (400 MHz, DMSO-d_6_): *δ* 4.17–4.12 (m, 1H, HOC ***H***); 3.89–3.86 (m, 1H, NC ***H***); 3.73 (s, 3H, NC ***H***_***3***_); 1.97–1.29 (***Cyclohexyl protons***).

^**13**^ **C-NMR** (100 MHz, DMSO-d_6_)**:** *δ* 171.2 (N ***C*** N); 119.6, 117.2 (*backbone carbons,* N ***C*** Cl = ***C*** ClN); 77.1 (***C*** HOH); 71.4 (N ***C*** H); 39.1 (N ***C*** H_3_); 35.1, 30.6, 24.3, 23.8 (***Cyclohexyl carbons***).

**MALDI- ToF:** 695.05472 Da attributable to bis-carbene structure [C_20_H_28_AuCl_4_N_4_O_2_]^+^

**Elemental Analysis:** calculated for C_10_H_14_AuCl_3_N_2_O, C 24.94, H 2.93, Au 40.90, Cl 22.08, N 5.82, O 3.32; Found: C 24.90, H 3.00, Cl 22.30, N 5.22.

### 7.3 Catalytic studies

#### 7.3.1 General procedure for hydroamination reaction promoted by gold(I) NHC complexes

In a Schlenk tube under an inert atmosphere, arylamine (1.0 mmol), alkyne (1.5 mmol), Au-NHC precatalyst (1% mol), silver salt (2% mol), and 1.0 mL of the solvent (listed in Table 2) were loaded. The flask was placed in preheated oil bath at 90°C and stirred for 16 h. After this time, the solvent was removed under reduced pressure, and at crude oil was added the internal standard (CH_2_Br_2_ 1.0 mmol in 0.7 mL of CDCl_3_). The yield of imines (listed in Table 4) was determined by ^1^H-NMR spectroscopy. The degree conversion was determined by integrating the ^1^H NMR signals of the two protons of the internal standard (CH_2_Br_2_) and the methyl group of the imine products. ^1^H NMR characterization of the isolated imines has been reported in the literature (Anderson et al., 2004; Wang et al., 2006; Chen et al., 2010; Peeters et al., 2013; Kumar et al., 2020).

## Data Availability

The structural data is deposited in the CCDC database repository: https://www.ccdc.cam.ac.uk/structures/ (CCDC 2260506).
