# Supplementary material for: Hydroamination of alkynes catalyzed by NHC-Gold(I) complexes: the non-monotonic effect of substituted arylamines on the catalyst activity
Source: Front Chem. 2023 Dec 6;11:1260726. doi: 10.3389/fchem.2023.1260726 (PMC10731675; doi:10.3389/fchem.2023.1260726)
Supplement: Supplementary file 1 [file DataSheet1.docx]

Supplementary Material

List of contents

[^1^H-NMR of 1a 3](#_Toc142474258)

[^13^C-NMR of 1a 4](#_Toc142474259)

[^1^H-NMR of 1b 5](#_Toc142474260)

[^13^C-NMR of 1b 6](#_Toc142474261)

[^1^H-NMR of 2a 7](#_Toc142474262)

[^13^C-NMR of 2a 8](#_Toc142474263)

[^1^H-NMR of 2b 9](#_Toc142474264)

[^13^C-NMR of 2b 10](#_Toc142474265)

[^1^H-NMR of PL-3 11](#_Toc142474266)

[^13^C-NMR of PL-3 12](#_Toc142474267)

[DEPT 135 of PL-3 13](#_Toc142474268)

[MALDI-ToF of PL-3 14](#_Toc142474269)

[^1^H-NMR of 3a 15](#_Toc142474270)

[^13^C-NMR of 3a 16](#_Toc142474271)

[ESI-MS of 3a 17](#_Toc142474272)

[^1^H-NMR 3b 18](#_Toc142474273)

[^13^C-NMR 3b 19](#_Toc142474274)

[MALDI-ToF 3b 20](#_Toc142474275)

[^1^H-NMR of PL-4 21](#_Toc142474276)

[^13^C-NMR of PL-4 22](#_Toc142474277)

[DEPT-135 of PL-4 22](#_Toc142474278)

[MALDI-ToF of PL-4 23](#_Toc142474279)

[^1^H-NMR of 4a 24](#_Toc142474280)

[^13^C-NMR of 4a 25](#_Toc142474281)

[MALDI-MS of 4a 26](#_Toc142474282)

[^1^H-NMR4b 27](#_Toc142474283)

[^13^C-NMR 4b 28](#_Toc142474284)

[MALDI-ToF 4b 29](#_Toc142474285)

[^1^H-NMR 5b 30](#_Toc142474286)

[^13^C-NMR 5b 31](#_Toc142474287)

[^1^H-NMR of PL-6 32](#_Toc142474288)

[^13^C-NMR of PL-6 33](#_Toc142474289)

[MALDI-ToF of PL-6 33](#_Toc142474290)

[^1^H-NMR 6a 34](#_Toc142474291)

[^13^C-NMR 6a 35](#_Toc142474292)

[MALDI-ToF of 6a 36](#_Toc142474293)

[^1^H-NMR of 6b 37](#_Toc142474294)

[^13^C-NMR of 6b 38](#_Toc142474295)

[MALDI-ToF of 6b 38](#_Toc142474296)

[^1^H-NMR of 7b 40](#_Toc142474297)

[13C-NMR of 7b 41](#_Toc142474298)

[^1^H-NMR of 8b 42](#_Toc142474299)

[^13^C-NMR of 8b 42](#_Toc142474300)

[Computational details 43](#_Toc142474301)

[Cartesian coordinates and energies of calculated structures. 43](#_Toc142474302)

[References 53](#_Toc142474303)

# ^1^H-NMR of 1a

1


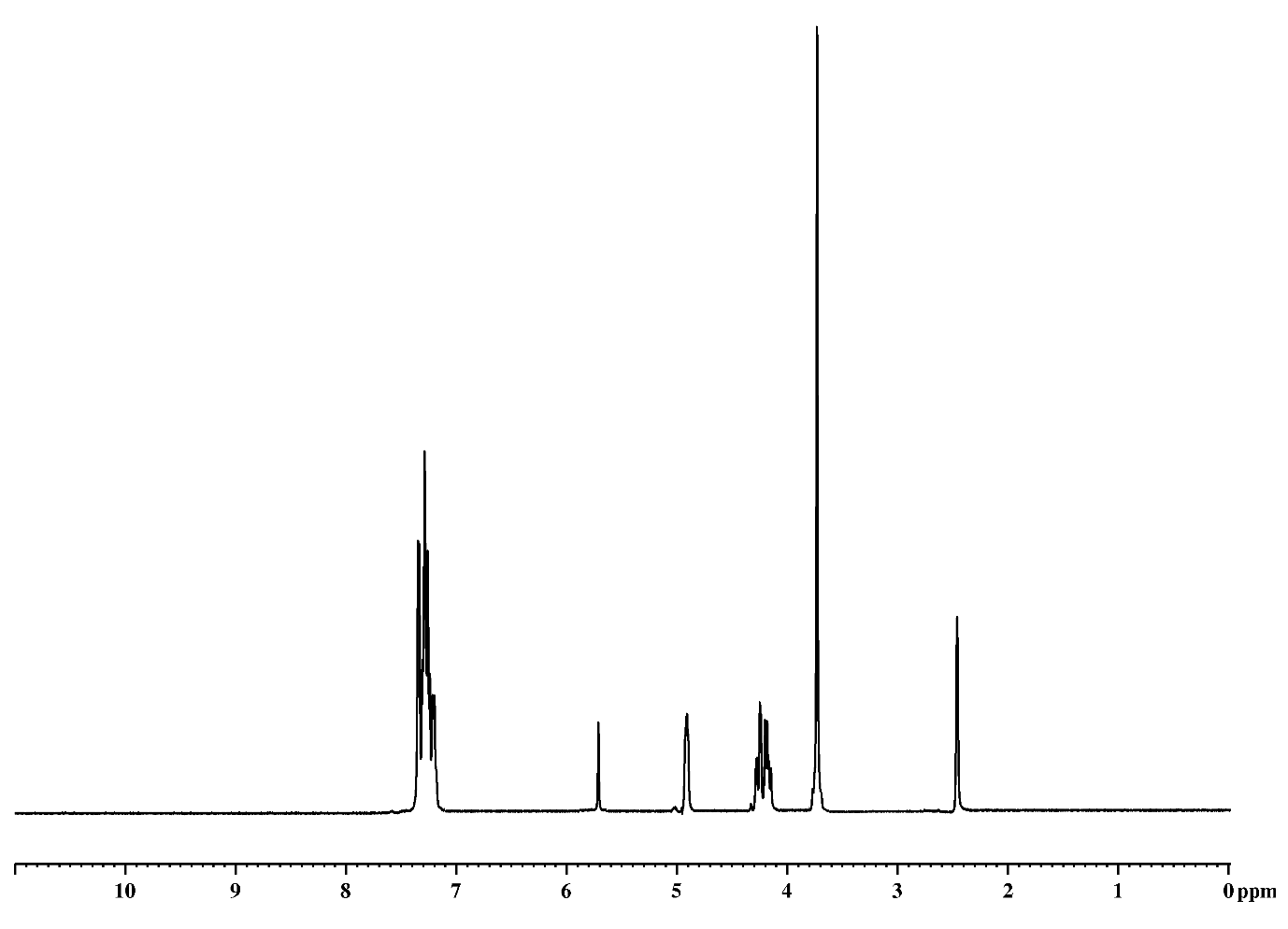


**DMSO**

Ph ring 2,3

OH

4

5

**^1^H-NMR** (400 MHz, DMSO-d_6_): *δ* 7.38-7.29 (m, 7H, ***Ph ring*** and ***backbone protons***); 5.68 (s, 1H, O***H***); 4.95-4.91 (m, 1H, C***H***OH); 4.31-4.19 (dd, 2H, NC***H_2_***); 3.75(s, 3H,C***H_3_***).

# ^13^C-NMR of 1a

**
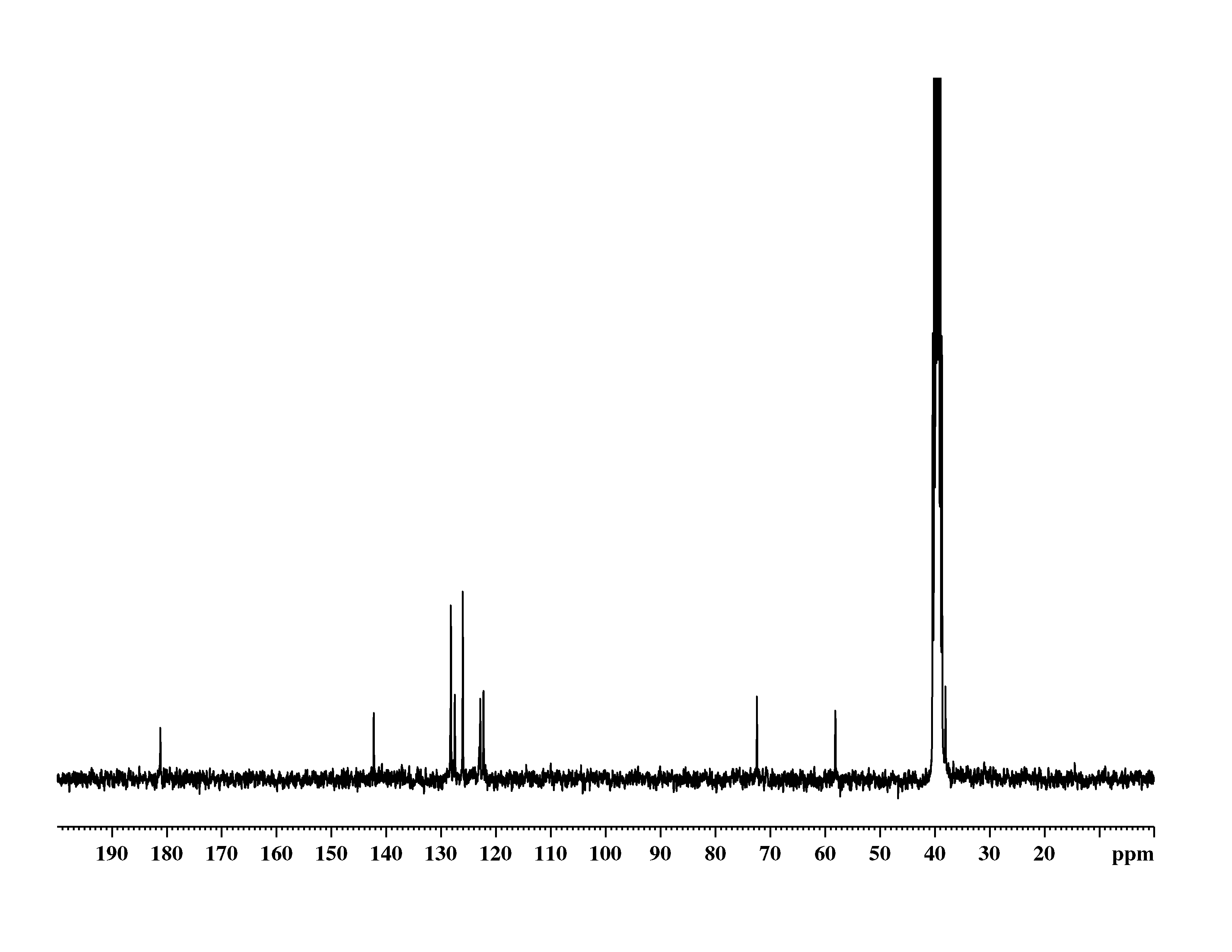
**

**DMSO**

1

4

5

2,3

Ph ring

6

**^13^C-NMR** (100 MHz, DMSO-d_6_): *δ* 181.2 (N***C***N); 142.3 (*ipso aromatic carbon,* **Ph ring**); 128.2, 127.6, 126.0 (***Ph ring***); 122.8, 122.3 (*backbone carbons,* N***C***H=***C***HN); 72.4 (***C***HOH); 58.1 (N***C***H_2_); 38.0 (***C***H_3_).

**MALDI-TOF (m/z):** 513.1456 attributable to bis carbene structure [C_24_H_30_AgN_4_O_2_]^+^

# ^1^H-NMR of 1b

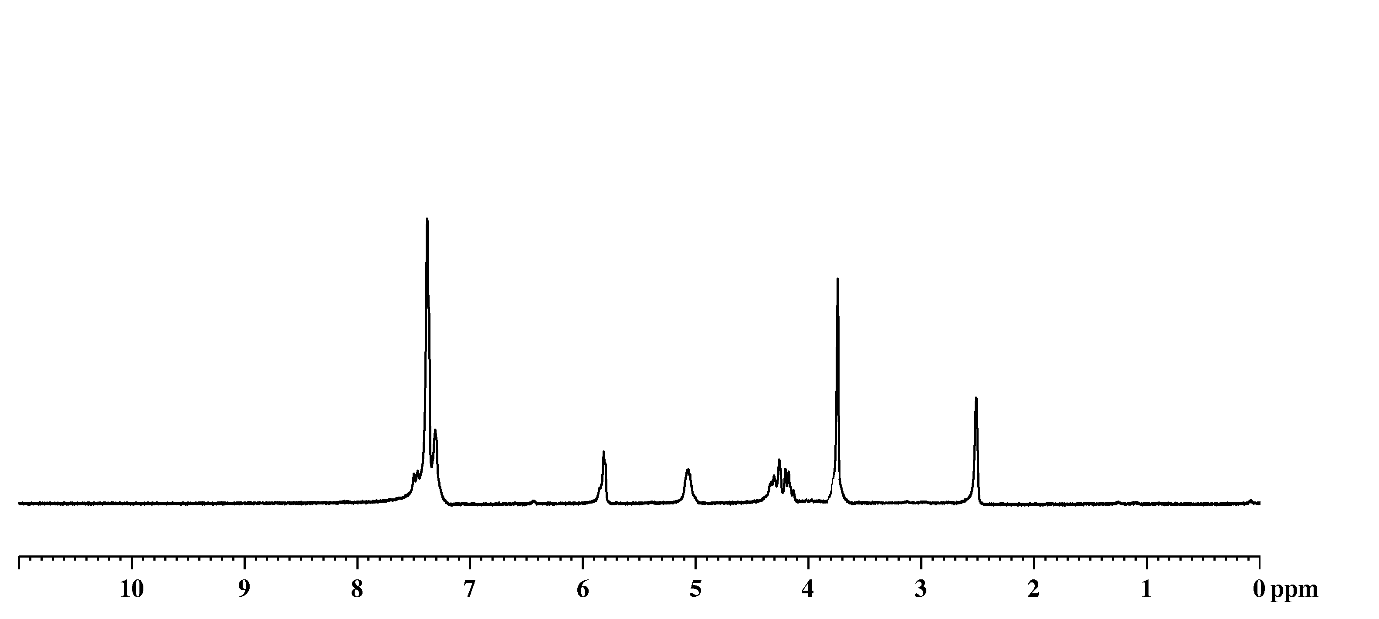


Ph ring - 4, 5

**DMSO**

1

2

3

OH

**^1^H-NMR** (400 MHz, DMSO-d_6_): *δ* 7.41-6.99 (m, 7H, ***Ph ring*** *and* ***backbone protons***); 4.31 (t, 1H, C***H***OH); 3.94 (d, 2H, NC***H_2_***); 3.71 (s, 3H, C***H_3_***).

# ^13^C-NMR of 1b

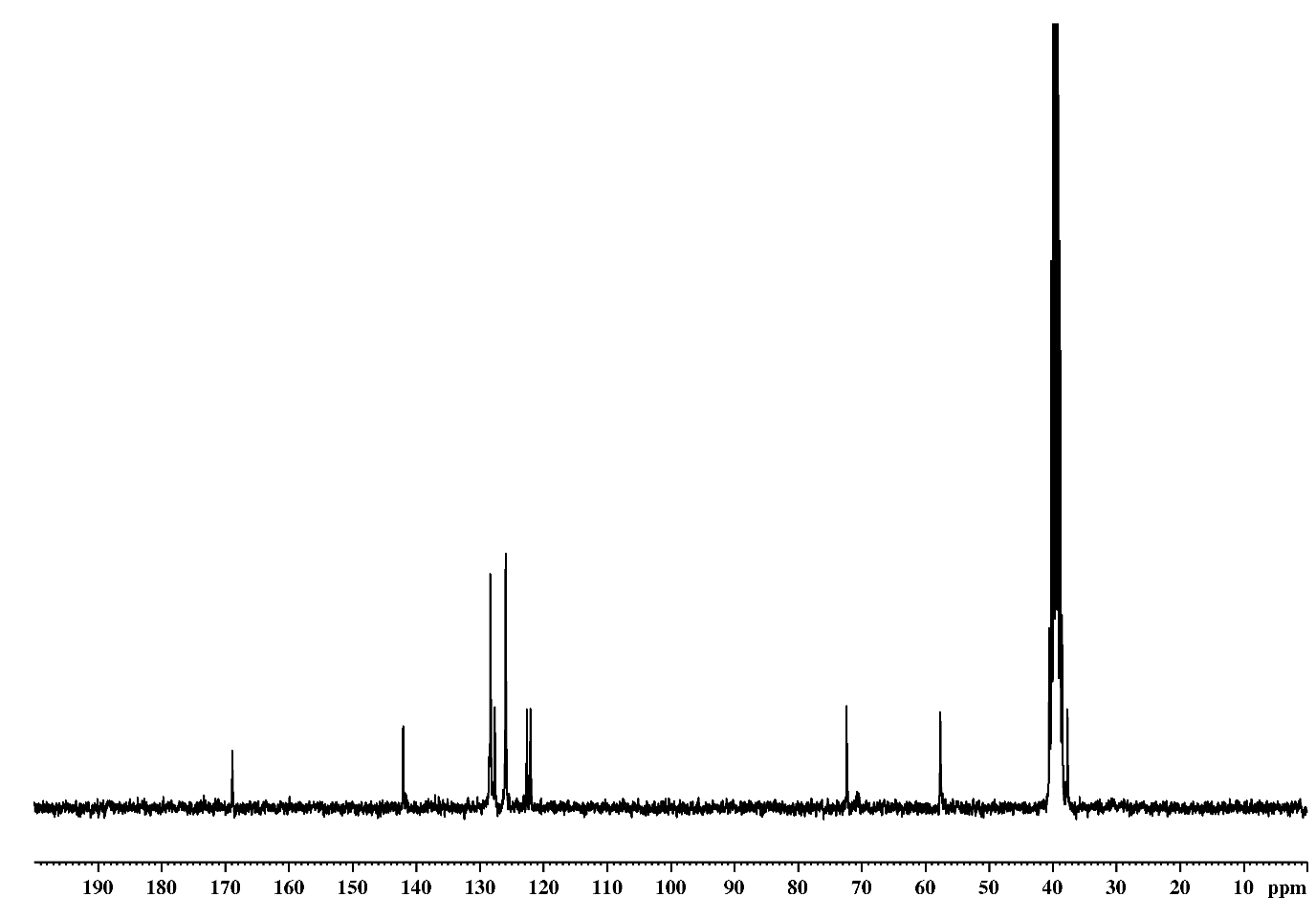


Ph ring

**DMSO**

1

6

4, 5

3

2

**^13^C-NMR** (100 MHz, DMSO-d_6_): *δ* 169.0 (N***C***N); 143.8 (*ipso aromatic carbon*, *,* **Ph ring**); 129.2, 127.5, 126.6 (***Ph* ring**); 122.9, 122.0 (*backbone carbons,* N***C***H=***C***HN); 73.2 (O***C***H); 58.3 (N***C***H_2_); 39.00 (N***C***H_3_).

**MALDI-TOF (m/z):** 601.06319 attributable to bis carbene structure [C_24_H_28_AuN_4_O_2_]^+^

# ^1^H-NMR of 2a

**
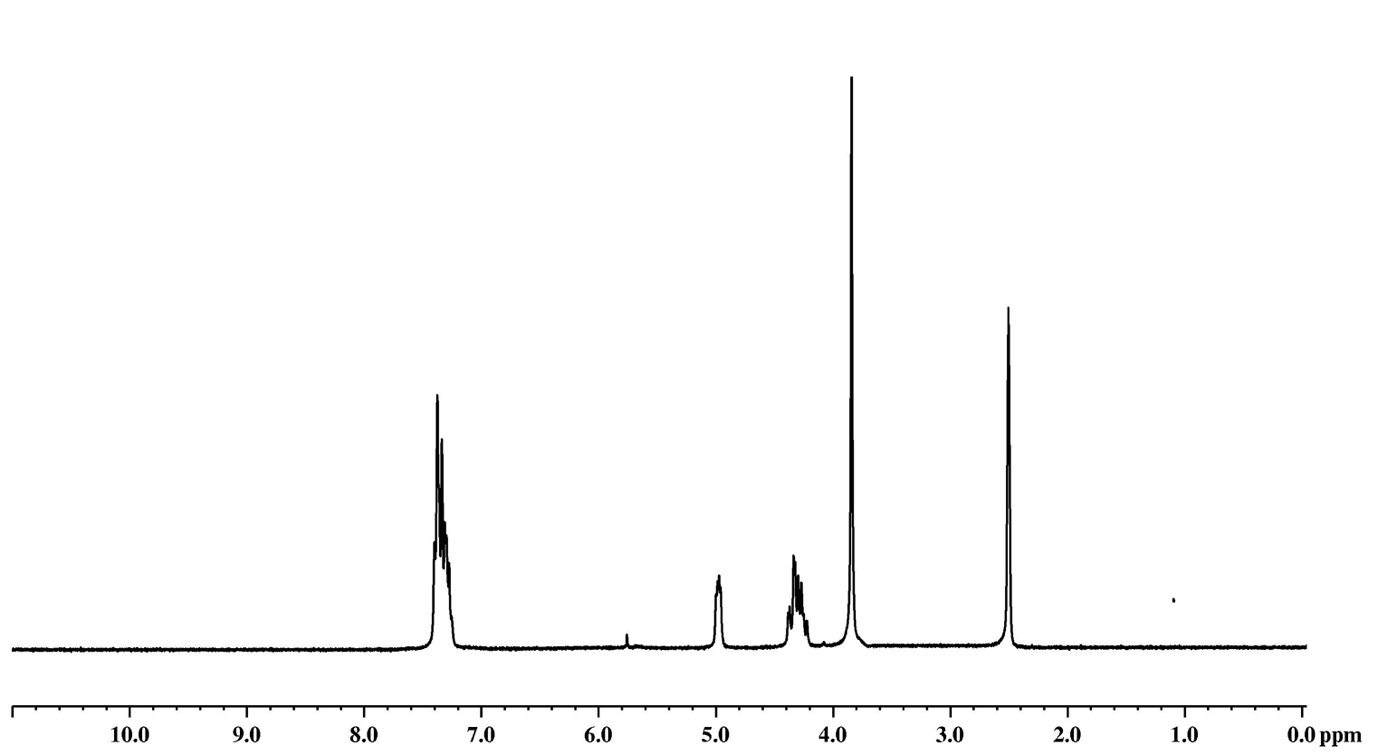
**

Ph group

3

2

1

DMSO

**^1^H-NMR** (400 MHz, DMSO-d_6_): *δ* 7.39 (m, 5H, ***Ph ring***); 4.90 (m, 1H, C***H***OH); 4.35 (dd, 2H, NC***H_2_***); 3.85 (s, 3H, C***H_3_***).

# ^13^C-NMR of 2a

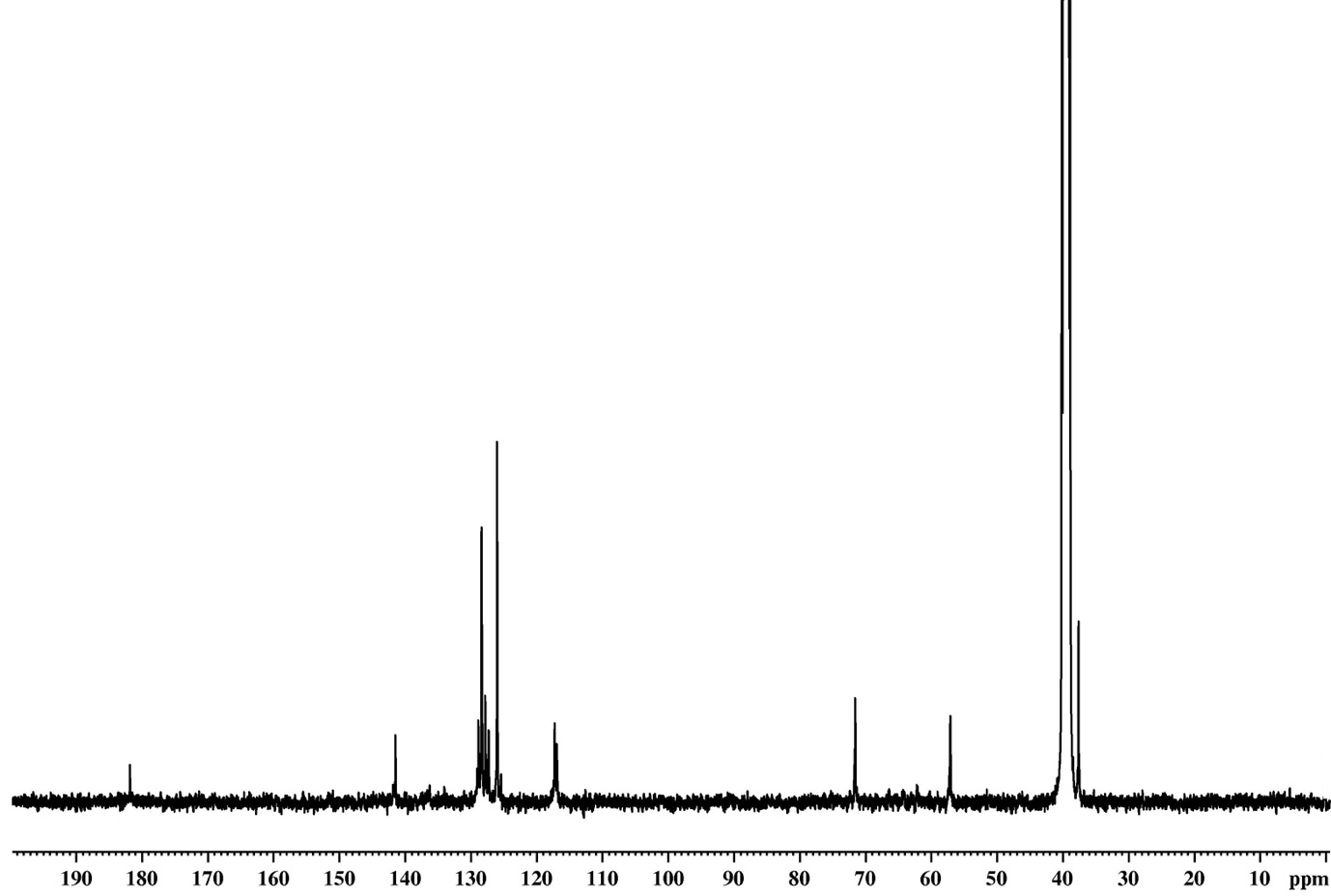


**DMSO**

Ph ring

6

2, 3

5

4

1

**^13^C-NMR** (75 MHz, DMSO-d_6_): *δ* 181.6 (N***C***N); 141.4 (*ipso aromatic carbon,* **Ph ring**); 128.3, 127.7, 125.9 (***Ph ring***); 117.2, 116.6 (*backbone carbons,* N***C***Cl=***C***ClN); 71.8 (***C***HOH); 56.9 (N***C***H_2_); 37.5 (***C***H_3_).

**MALDI-TOF (m/z):** 648.9909 attributable to bis carbene structure [C_24_H_26_AgCl_4_N_4_O_2_]^+^

# ^1^H-NMR of 2b

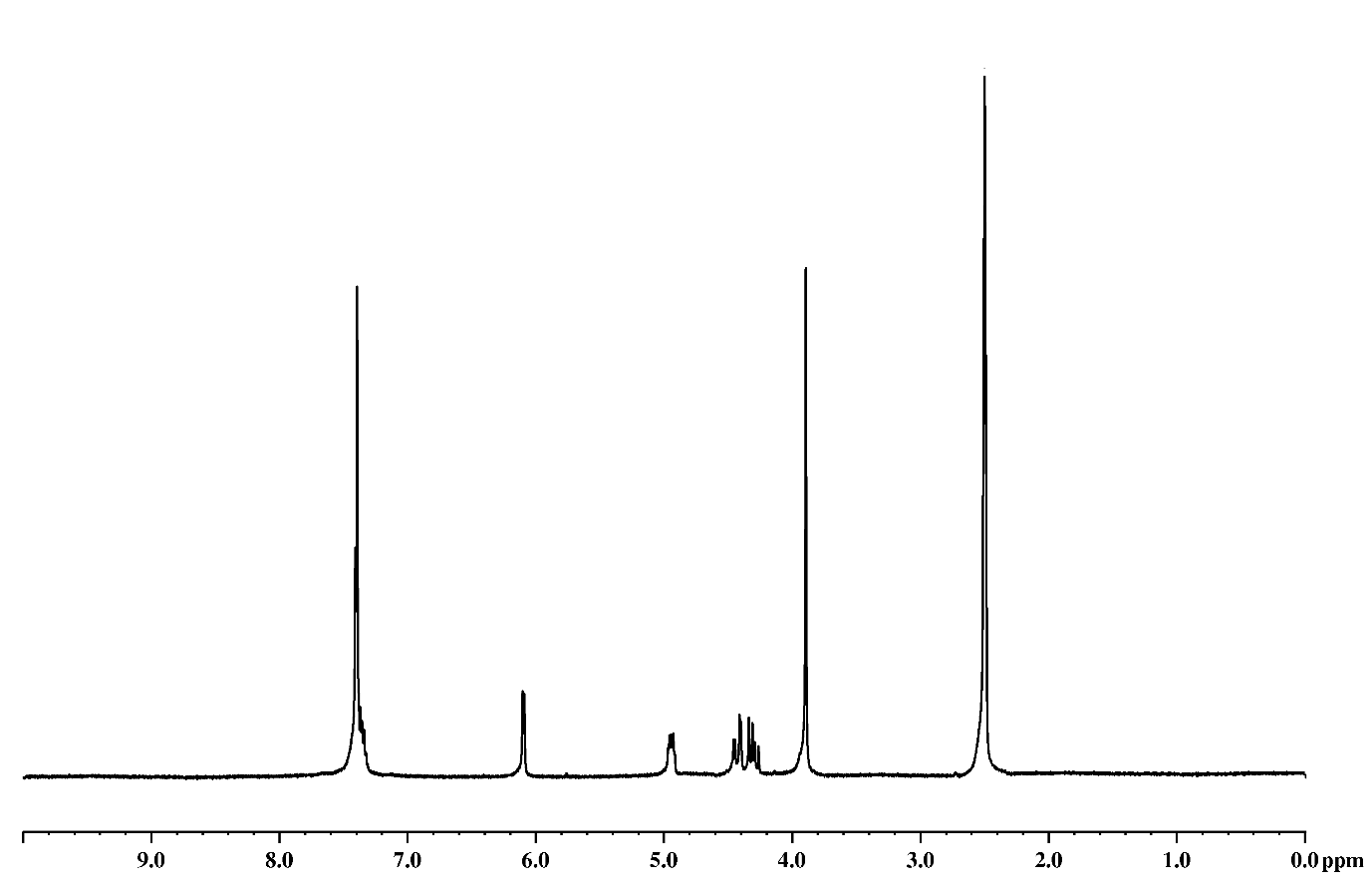


1

3 2

Ph ring

**DMSO**

OH

**^1^H-NMR** (400 MHz, DMSO-d_6_): *δ* 7.33 (m, 5H, ***Ph ring***); 5.88 (d, 1H, O***H***); 5.13 (m, 1H, C***H***OH); 4.23 (m, 2H, NC***H_2_***); 3.82 (s, 3H, C***H_3_***).

# ^13^C-NMR of 2b

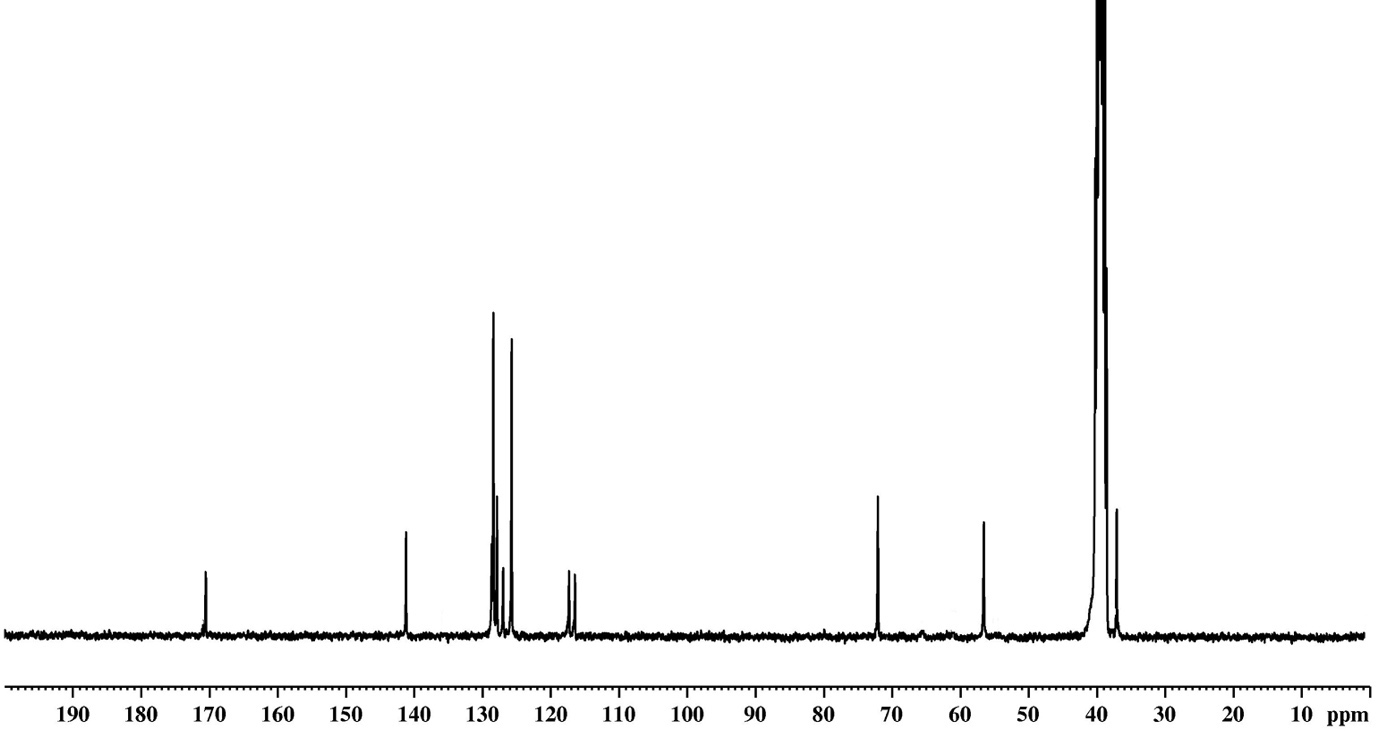


3 2

4, 5

**DMSO**

Ph ring

6

1

**^13^C-NMR** (100 MHz, DMSO-d_6_*) δ*: 170.7 (N***C***N); 141.2 (*ipso aromatic carbon,* **Ph ring**); 128.5, 127.0, 125.7 (***Ph ring***); 117.3, 116.4 (*backbone carbons,* N***C***Cl=***C***ClN); 72.0 (***C***HOH); 56.6 (N***C***H_2_); 37.1 (N***C***H_3_).

**MALDI-TOF (m/z):** 739.03526 attributable to bis carbene structure [C_24_H_24_N_4_O_2_Cl_4_Au]^+^

# ^1^H-NMR of PL-3

**DMSO**


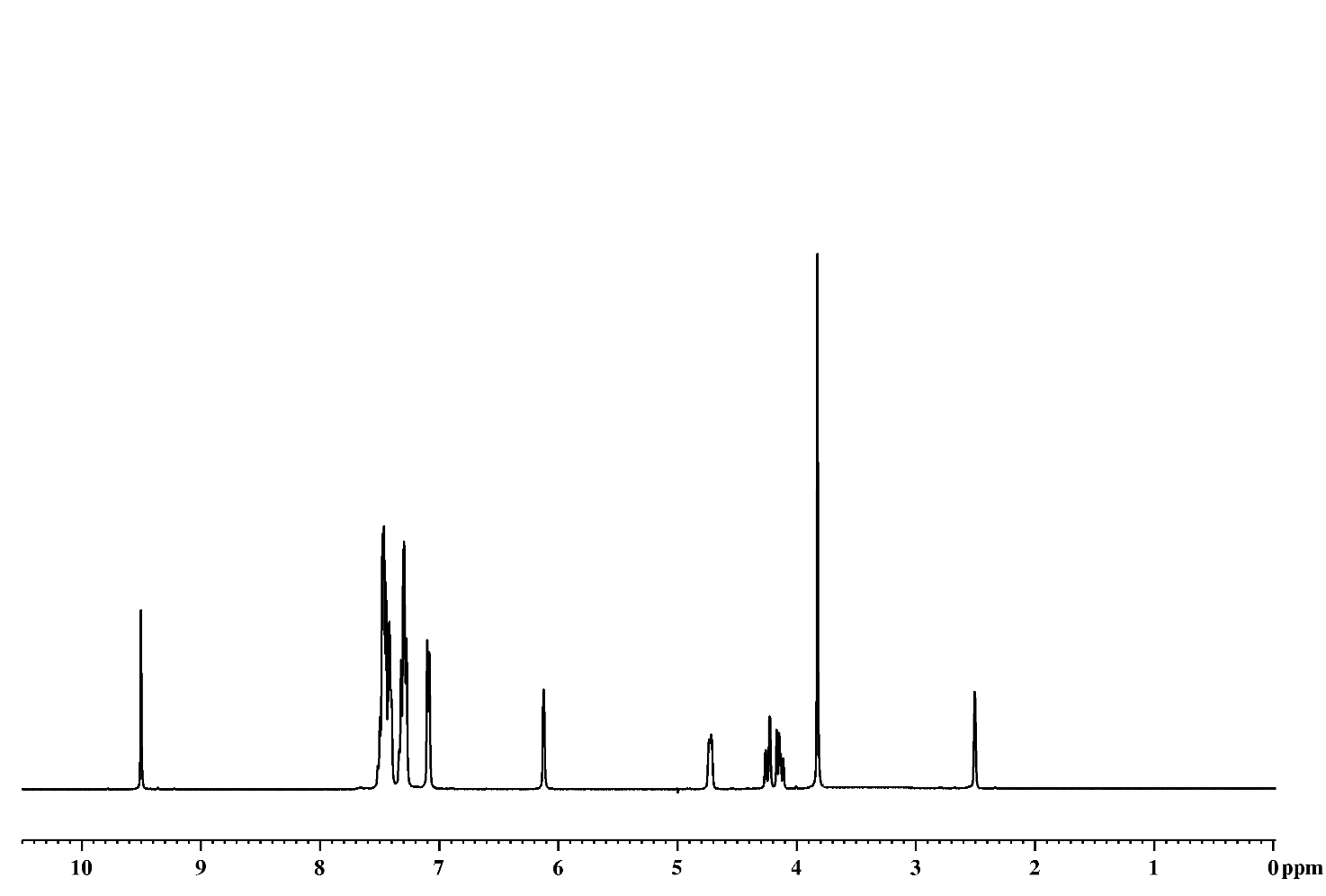


5

Ph rings

OH-group

4

2,3

1

**^1^H-NMR** (400 MHz, DMSO-d_6_): *δ* 9.50 (s, 1H, NC***H***N); 7.46-7.11 (m, 15H, ***Ph rings***); 6.12 (s, 1H, O***H***); 4.72 (m, 1H, OC***H***, *J_anti_ 7.37 Hz, J_gauche_ 5.80 Hz*); 4.26-4.11 (m, 2H, NC***H_2,_*** *J_gem_14.5 Hz, J_anti_ 7.37 Hz, J_gauche_ 5.80 Hz*); 3.84 (s, 3H, NC***H_3_***).

# ^13^C-NMR of PL-3

**DMSO**


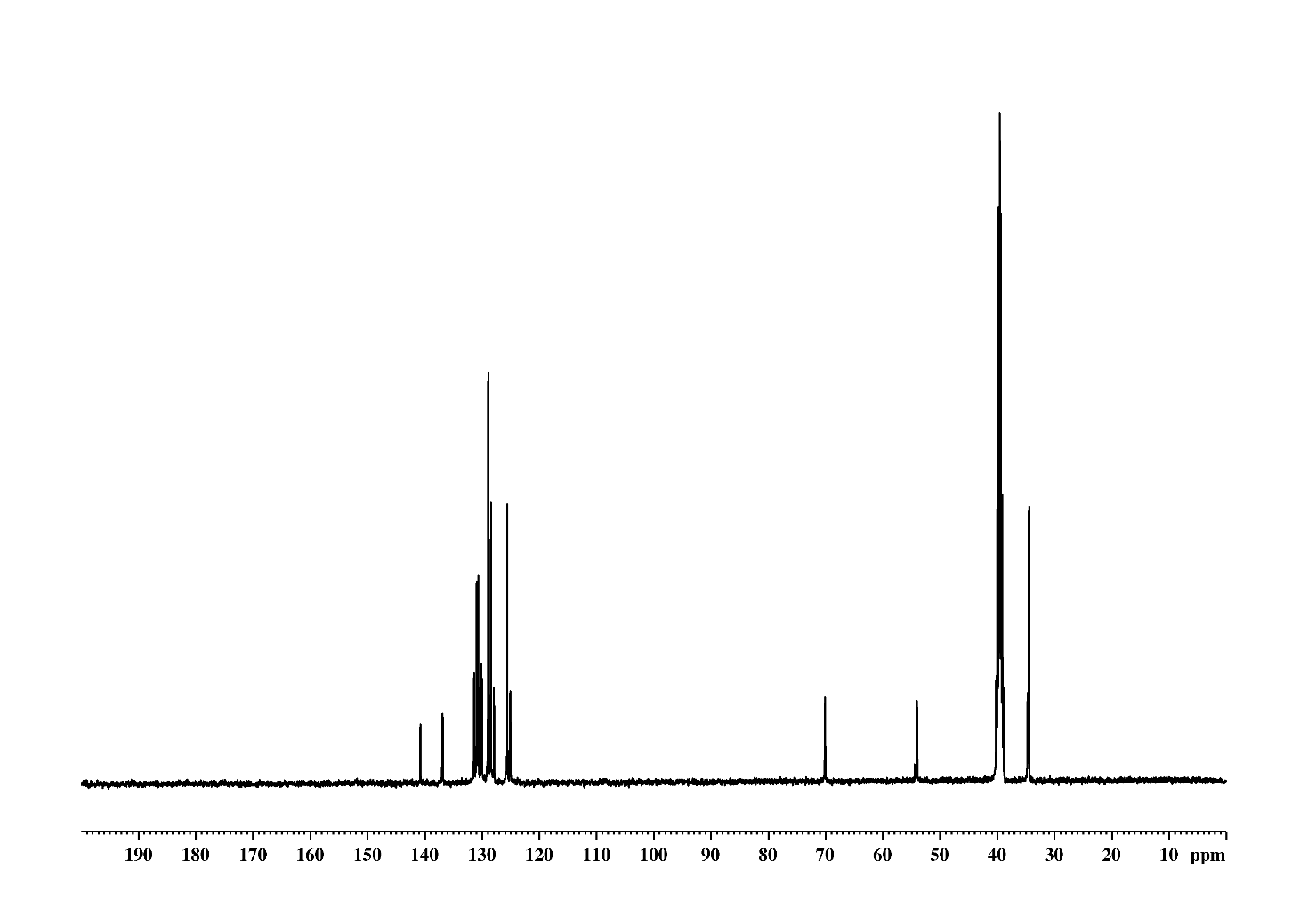


1’

6

Ph ring
2’,3’,4’,5’,6’,7’,8’,9’10’,11’,12’

3

2

1


**^13^C-NMR** (100 MHz, DMSO-d_6_): *δ* 140.7 *(ipso aromatic carbon*, **Ph-ring**); 136.8 (N***C***N); 131.3-125.5 (*aromatic carbons*, **Ph rings**), 125.5-125.0 (*backbone carbons*, N***C***Ph=***C***PhN), 70.0

(O***C***H); 54.0 (N***C***H_2_); 34.4 (N***C***H_3_).

# DEPT 135 of PL-3


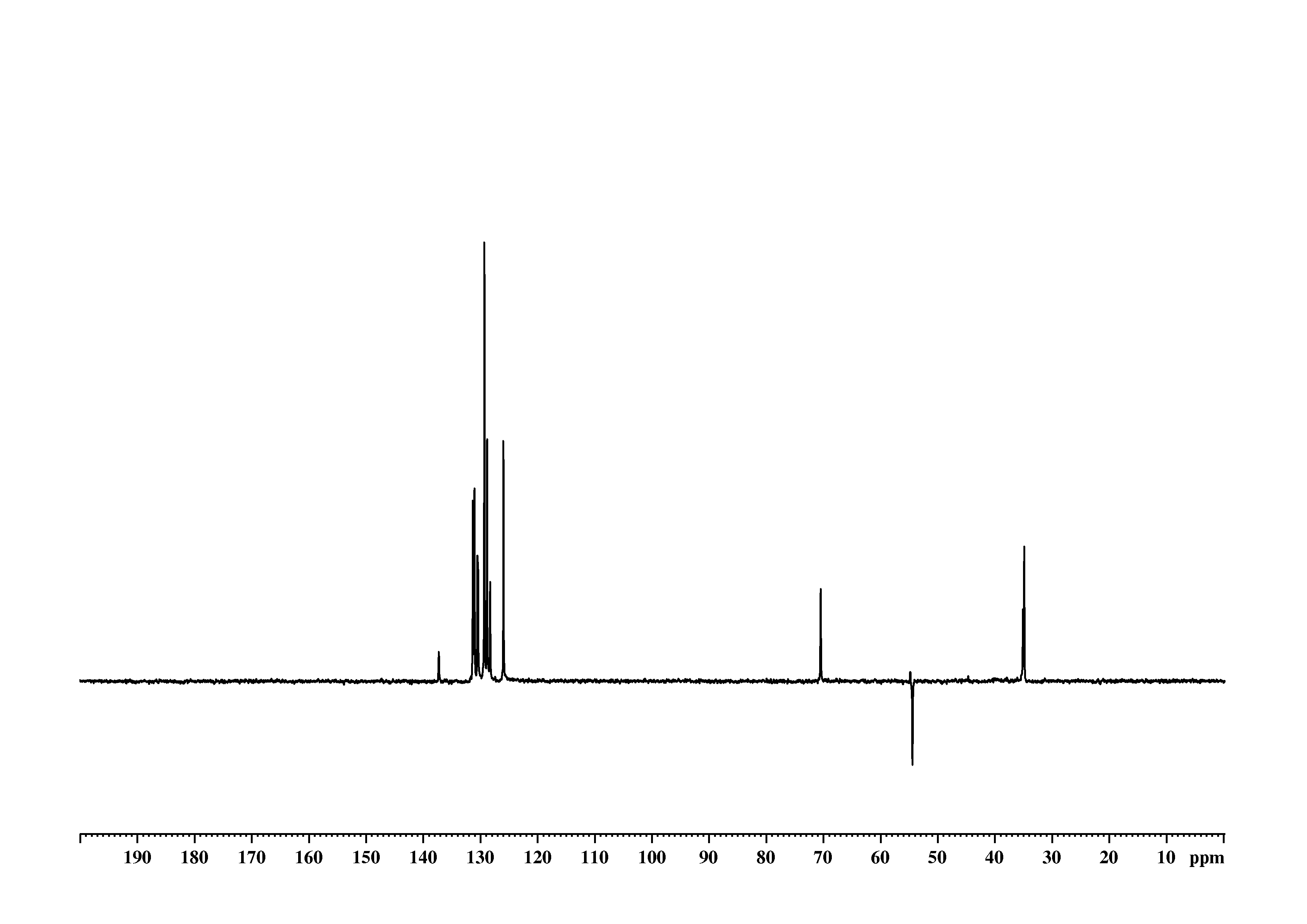


**Comparison among ^13^C-NMR and DEPT135 spectra of PL-3 from 124 to 142 ppm**


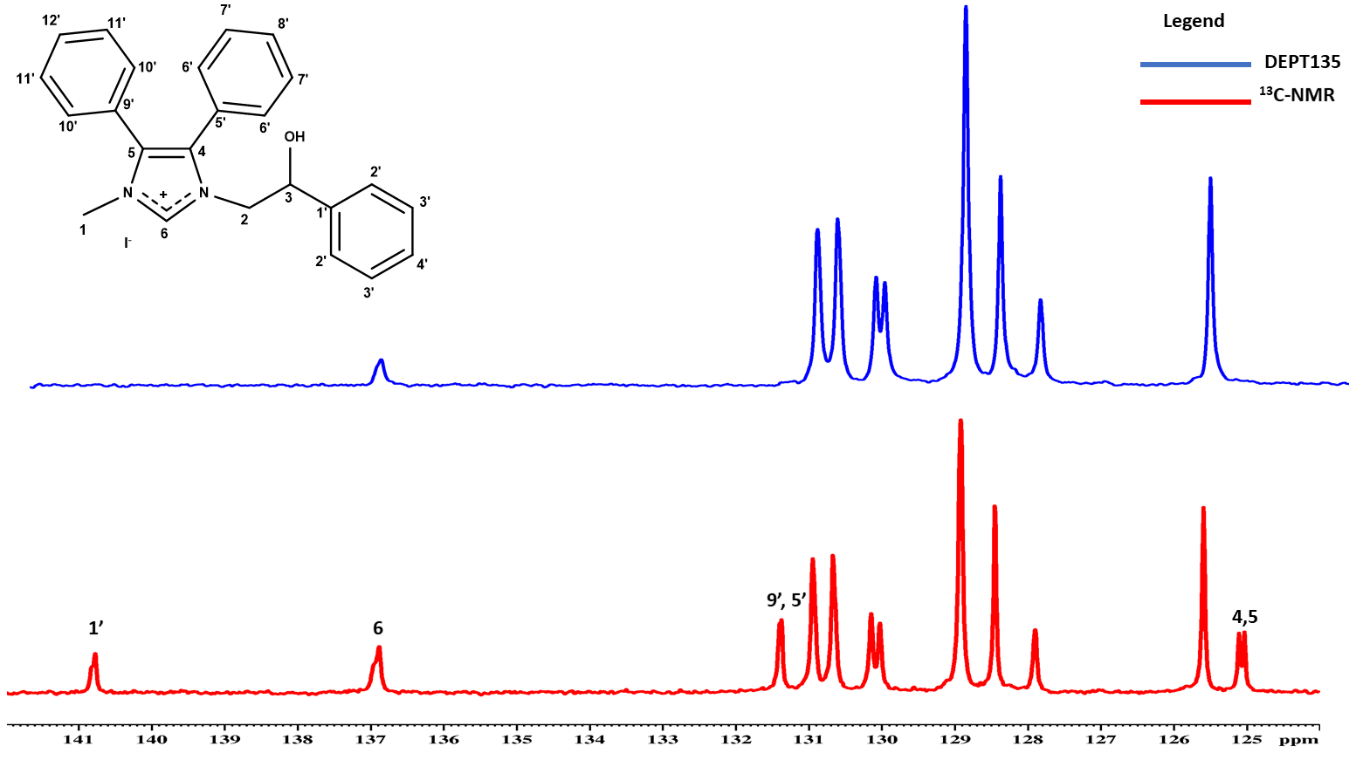


# MALDI-ToF of PL-3


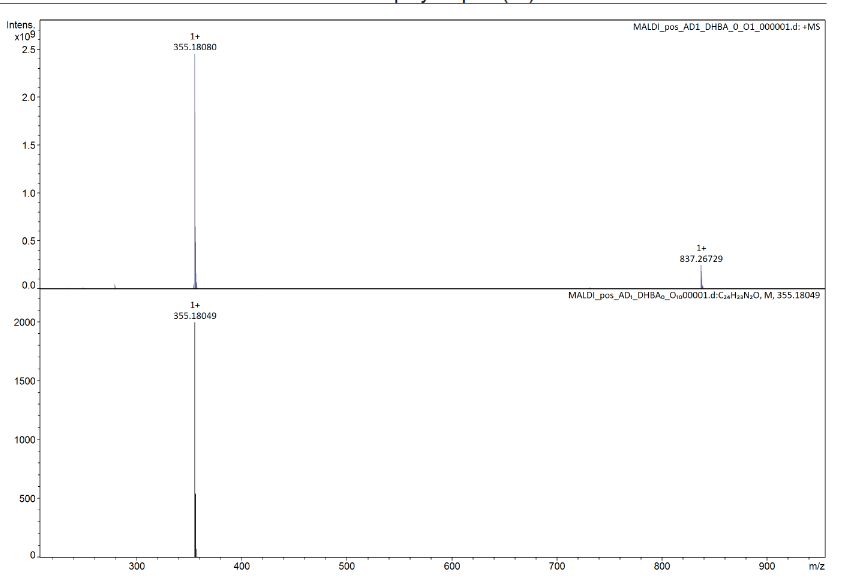


**MALDI-ToF (m/z):** 355.18080 Da attributable to the cationic portion of the imidazolium salt [C_24_H_23_N_2_O]^+^;

# ^1^H-NMR of 3a

**DMSO**


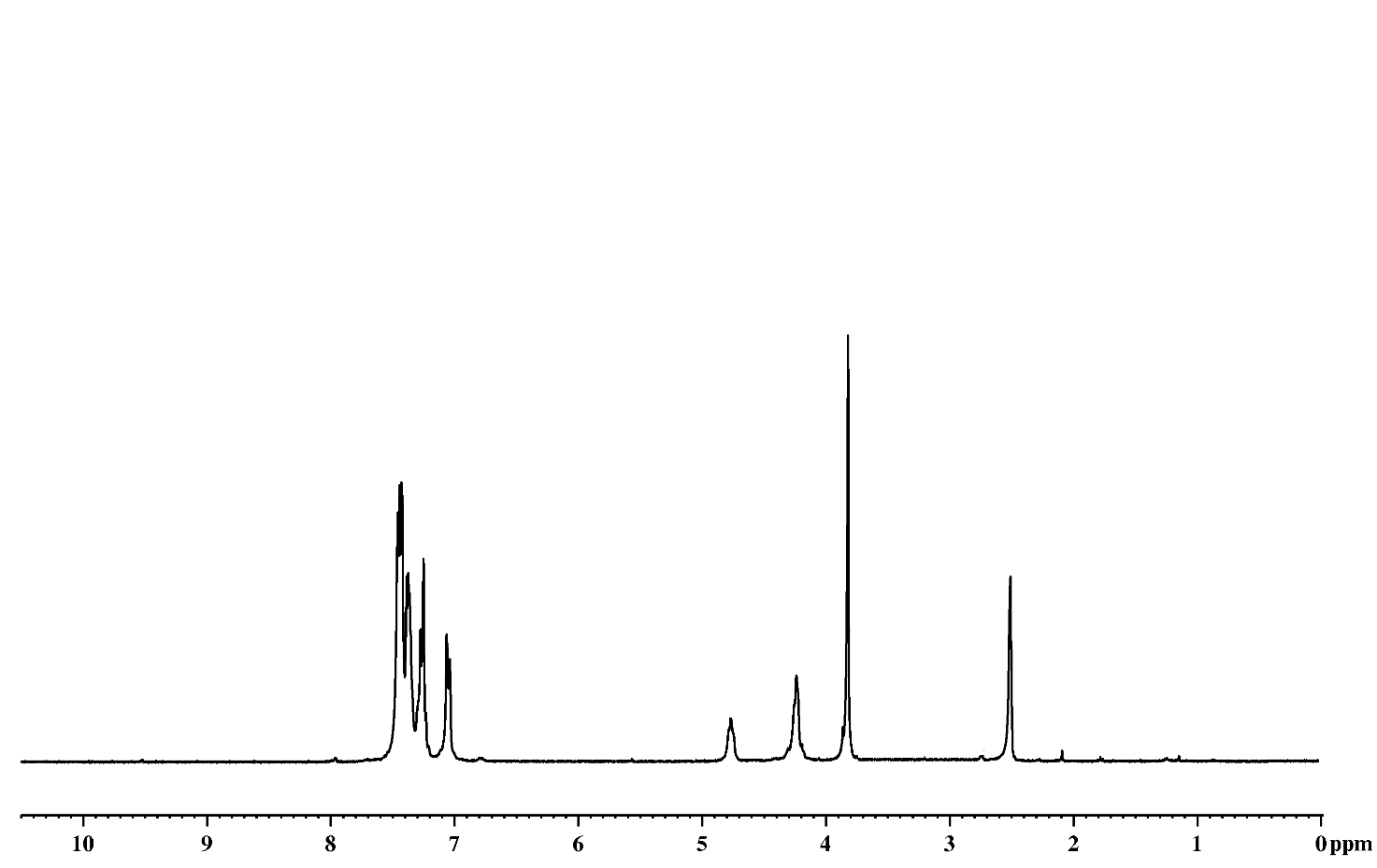


1

2,3

4

Ph rings

**^1^H-NMR** (400 MHz, DMSO-d_6_): *δ* 7.46-7.04 (m, 15H, ***Ph rings***); 4.76 (m, 1H, OC***H,*** *J_anti_ 7.73 Hz, J_gauche_ 5.00 Hz*); 4.19 (m, 2H, NC***H_2_,*** *J_gem_ 14.0 Hz, J_anti_ 7.73 Hz, J_gauche_ 5.00 Hz*); 3.82 (s, 3H, NC***H_3_***).

# ^13^C-NMR of 3a

**DMSO**


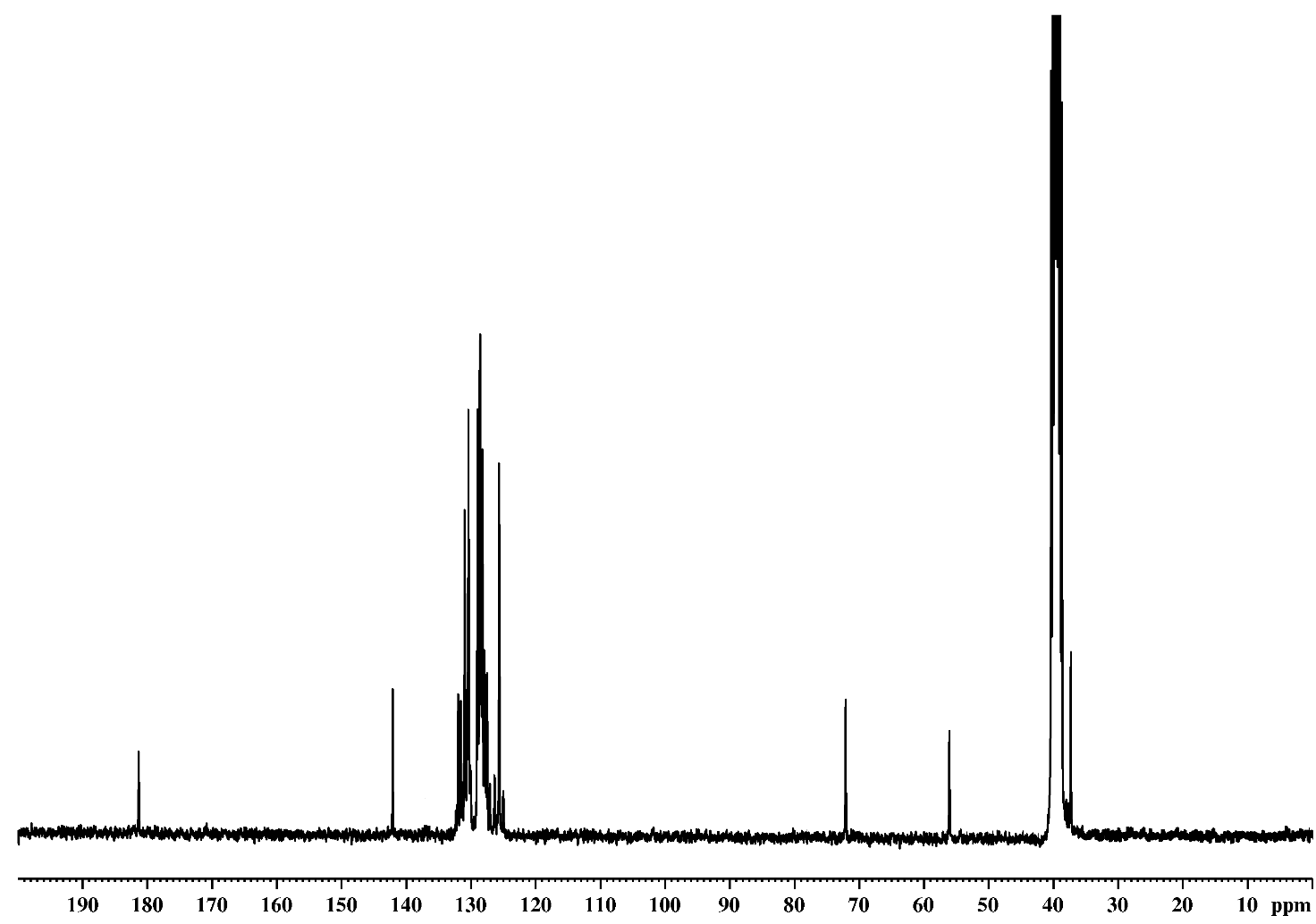


6

Ph rings

backbone 4, 5

3

2

1

**^13^C-NMR** (100 MHz, DMSO-d_6_): *δ* 181.4 (N***C***N); 142.2 *(ipso aromatic carbon*, ***Ph ring***); 131.9-125.6 (*aromatic carbons*, ***Ph rings***); 72.2 (O***C***H); 56.0 (N***C***H_2_); 37.4 (N***C***H_3_).

# ESI-MS of 3a


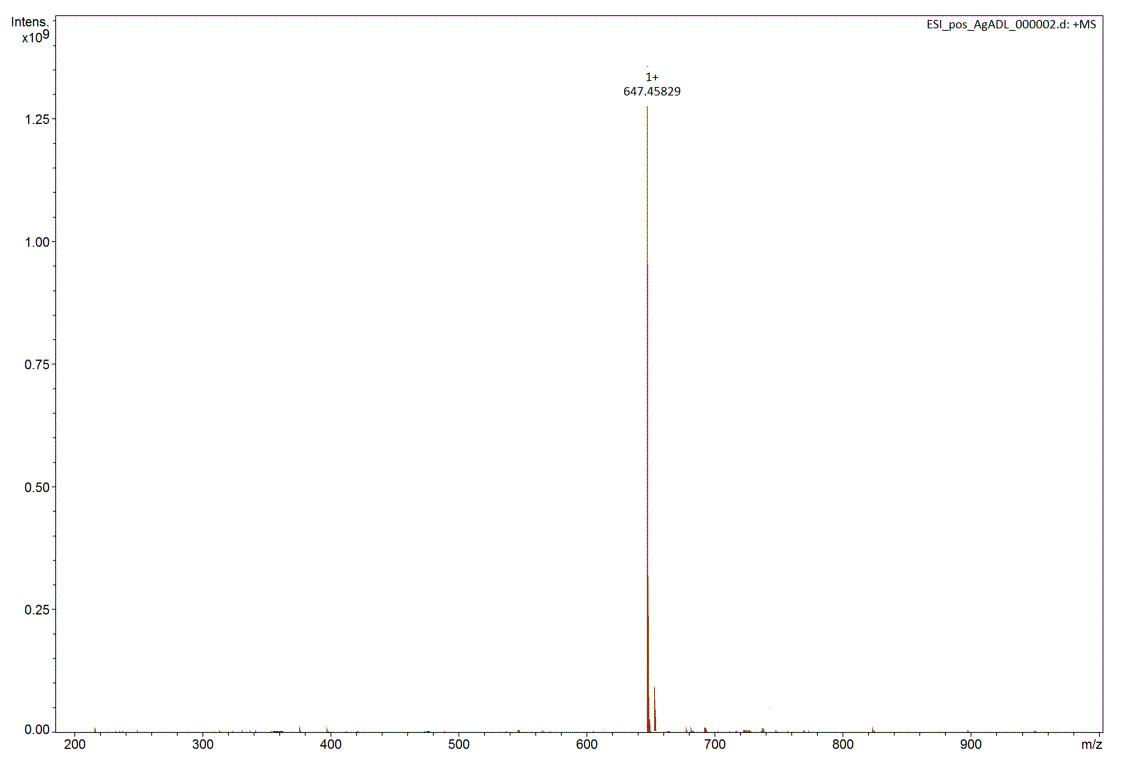


**ESI (m/z):** 647.45829 Da attributable to bis-carbene structure [C_35_H_30_AgN_4_O_2_]^+^.

# ^1^H-NMR 3b

**DMSO**


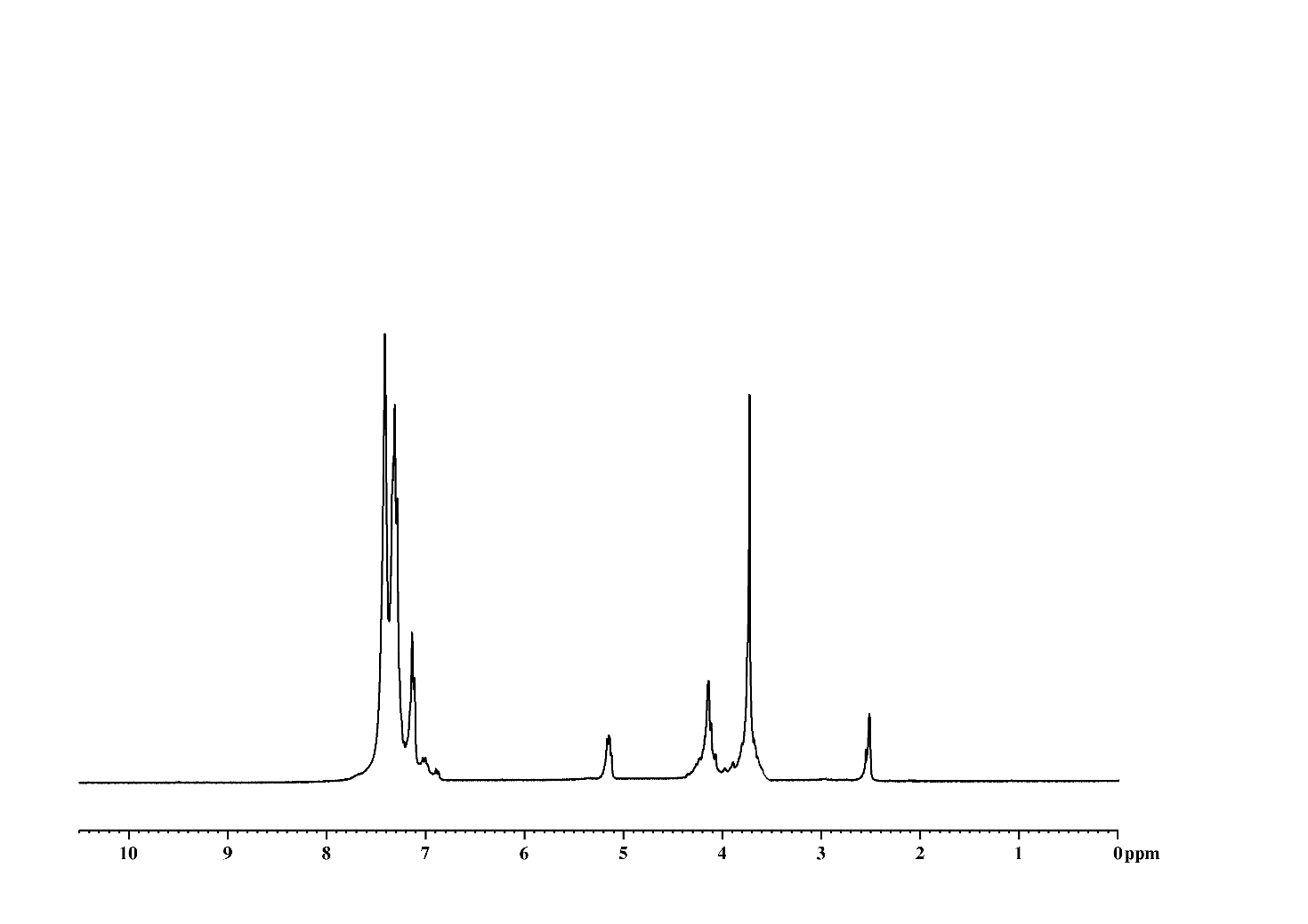


1

2, 3

4

Ph-rings

**^1^H-NMR** (400 MHz, DMSO-d_6_): *δ* 7.36-7.14 (m, 15H, ***Ph rings***); 5.13 (m, 1H, OC***H***, *J_anti_ 7.00 Hz, J_gauche_ 5.30 Hz*); 4.11 (m, 2H, NC***H_2_***, *J_gem_ 13.89 Hz, J_anti_ 7.00 Hz, J_gauche_ 5.30 Hz*); 3.72 (s, 3H, NC***H_3_***).

# ^13^C-NMR 3b

1’


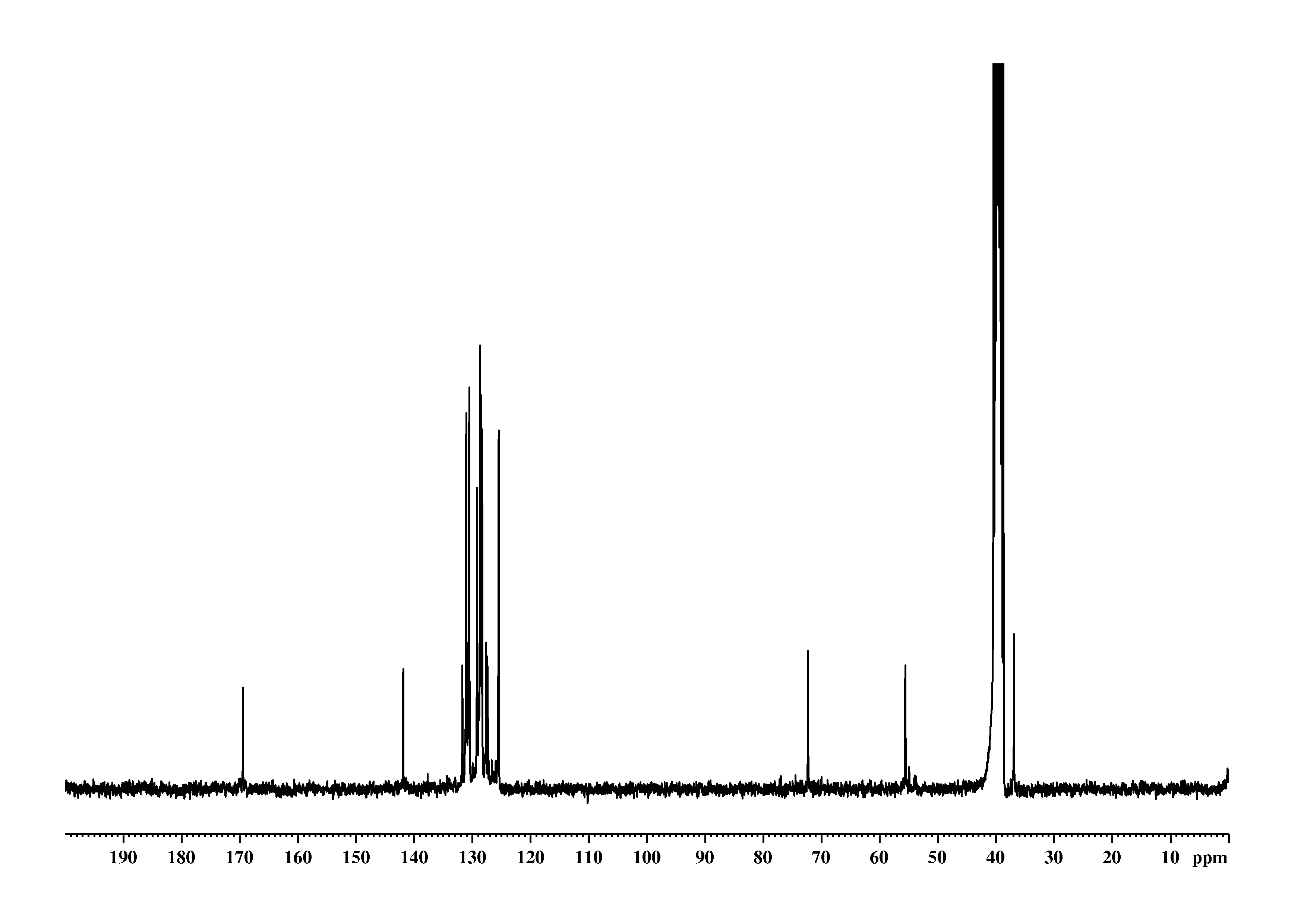


1

2

3

Ph-rings

*Backbone* 4, 5

6

**DMSO**

**^13^C-NMR** (100 MHz, DMSO-d_6_): *δ* 169.5 (N***C***N); 141.8 *(ipso aromatic carbon*, ***Ph ring***); 131.7-125.5 (*aromatic carbons*, ***Ph rings***); 72.3 (O***C***H); 55.6 (N***C***H_2_); 36.9 (N***C***H_3_).

# MALDI-ToF 3b

+


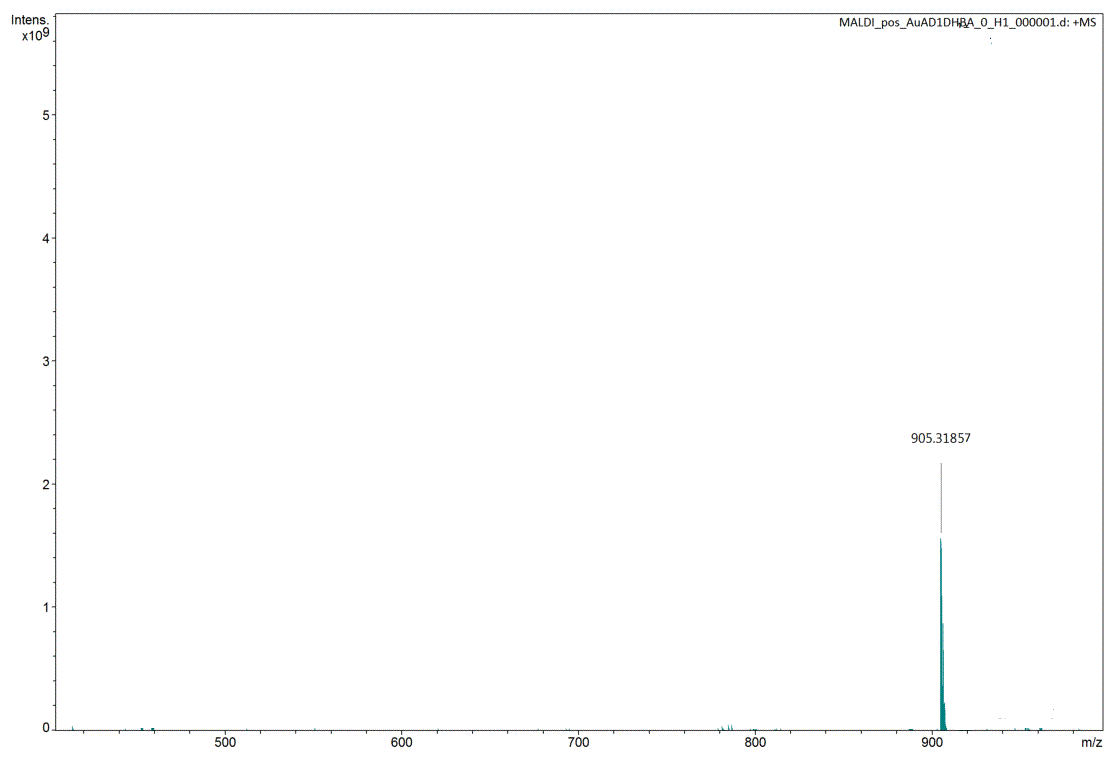


**MALDI-ToF (m/z):** 905.31857 Da attributable to bis-carbene structure [C_48_H_44_AuN_4_O_2_]^+^.

# ^1^H-NMR of PL-4

**DMSO**


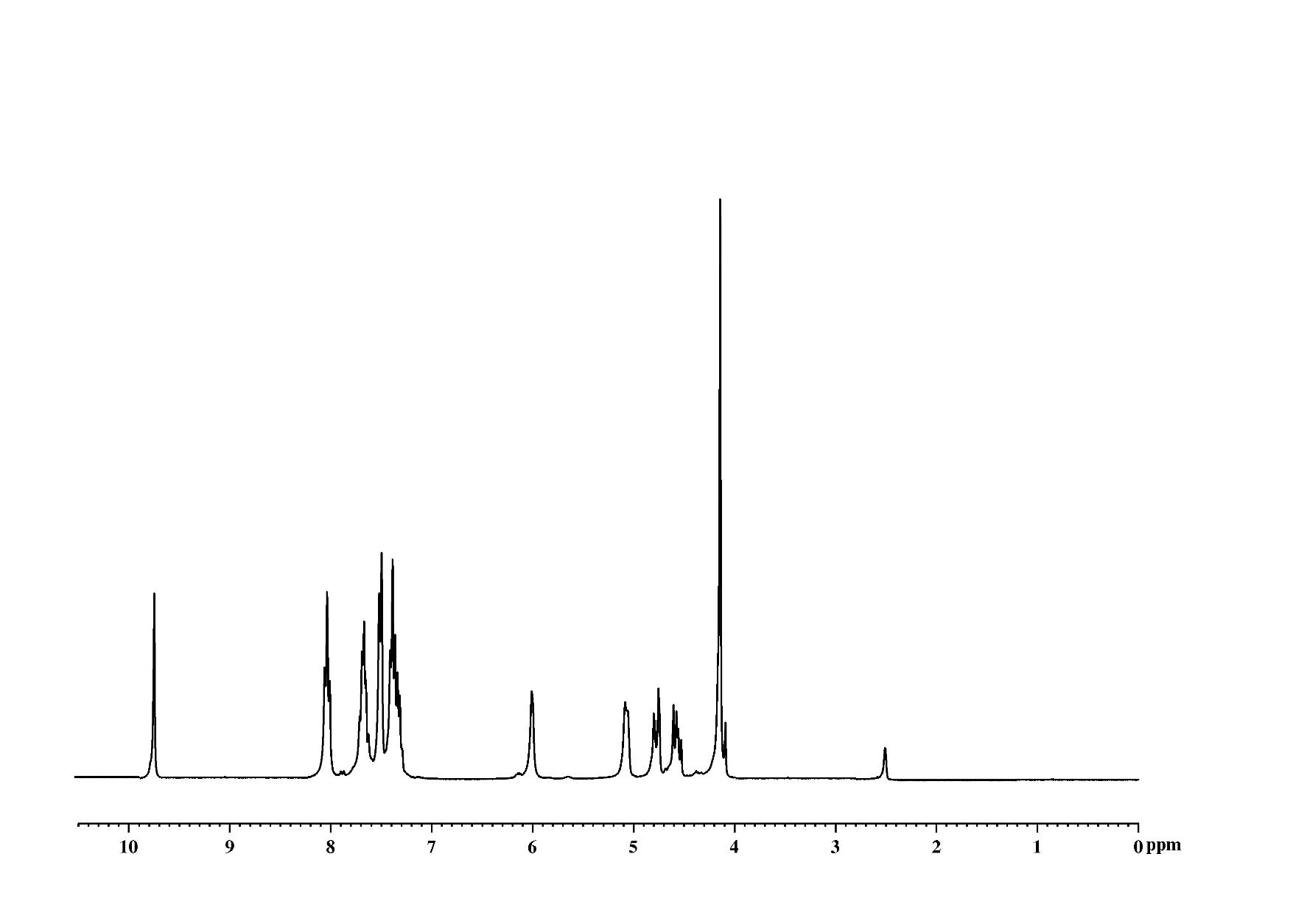


5

Ph rings

OH-group

4

2, 3

1

**^1^H-NMR** (400 MHz, DMSO-d_6_): *δ* 9.77 (s, 1H, NC***H***N); 8.06-7.31 (m, 9H, ***Ph rings***); 5.99 (d, 1H, O***H***); 5.10-5.07 (dd, 1H, OC***H****,* *J_anti_ 7.58 Hz, J_gauche_ 5.50 Hz*); 4.61-4.52 (m, 2H, NC***H_2_*_,_** *J_gem_14.7 Hz, J_anti_ 7.58 Hz, J_gauche_5.50 Hz*); 4.14 (s, 3H, NC***H_3_***).

# ^13^C-NMR of PL-4

**DMSO**


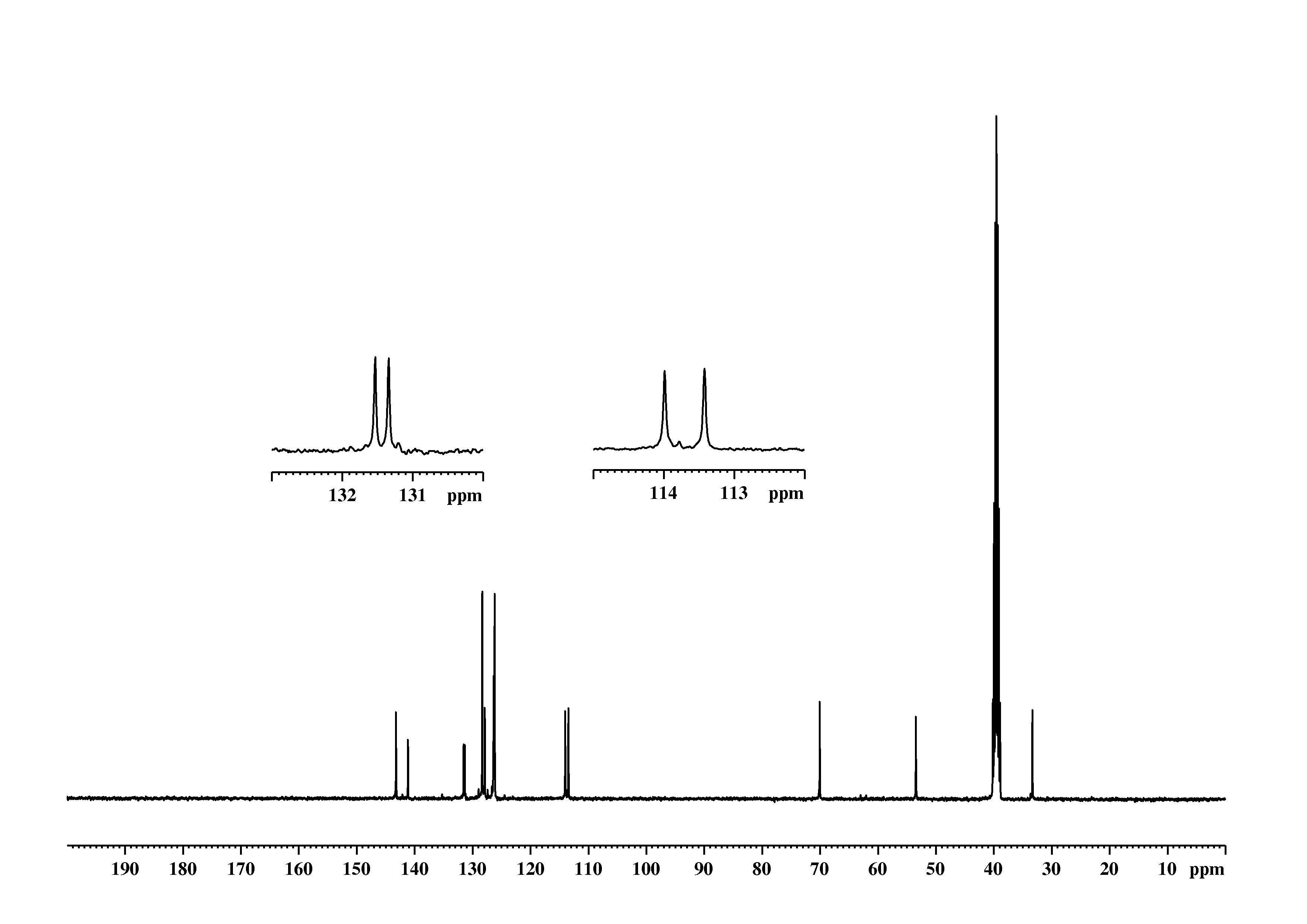


6

1’

4, 5

8, 5’

Ph-ring

(2’,3’,4’,7’,6’)

3

2

1

**^13^C-NMR** (100 MHz, DMSO-d_6_): *δ* 144.0 (N***C***N); 141.9 (*ipso aromatic carbon*, ***Ph-ring***); 131.6-131.3 (*backbone carbons*, N***C***=***C***N); 128.3, 127.9, 126.3, 126.1 (*aromatic carbons*, ***Ph rings***); 114.0, 113.4 (*aromatic carbons*, ***Ph rings***); 70.0 (O***C***H); 54.0 (N***C***H_2_), 34.4 (N***C***H_3_).

# DEPT-135 of PL-4


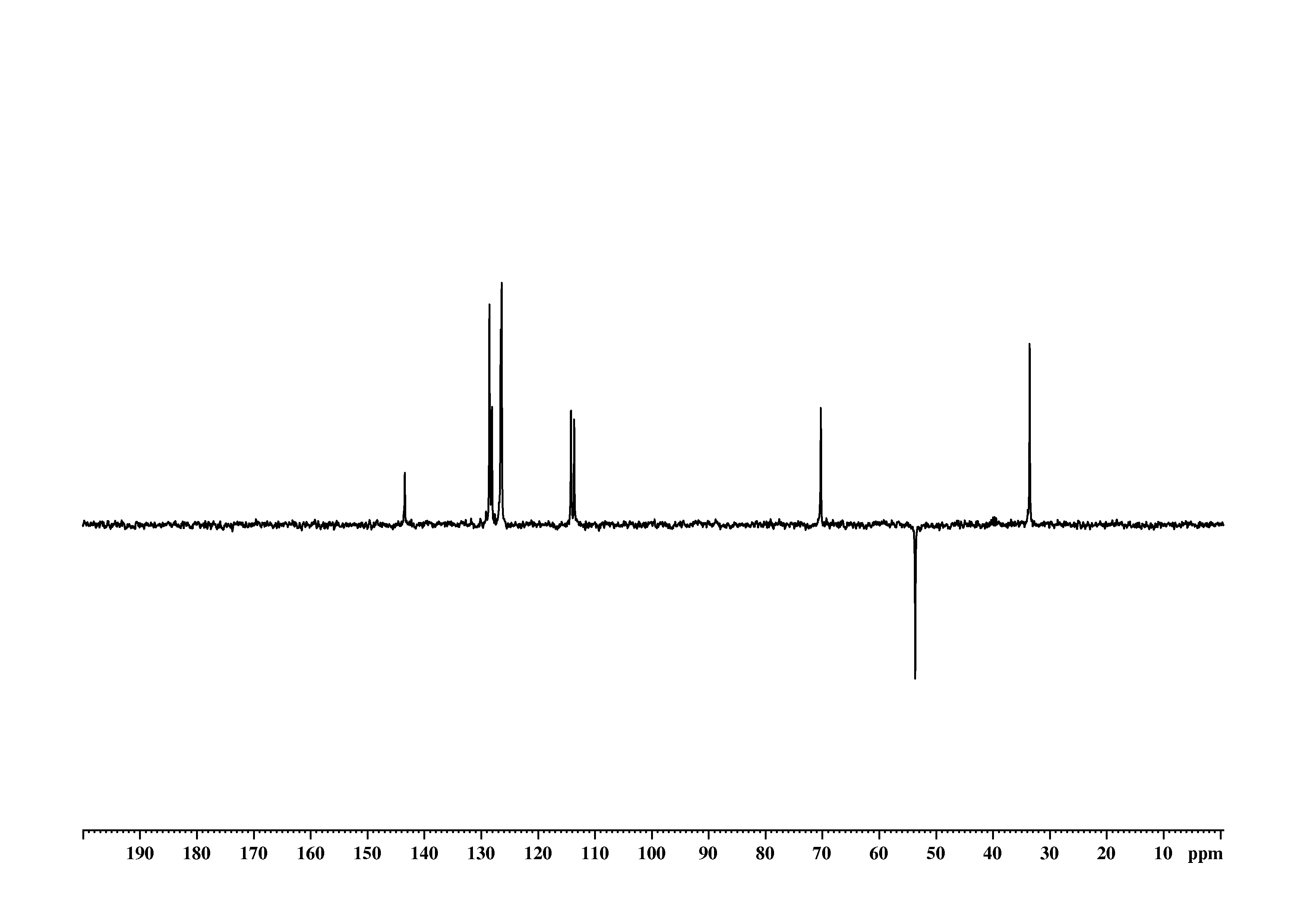


# MALDI-ToF of PL-4


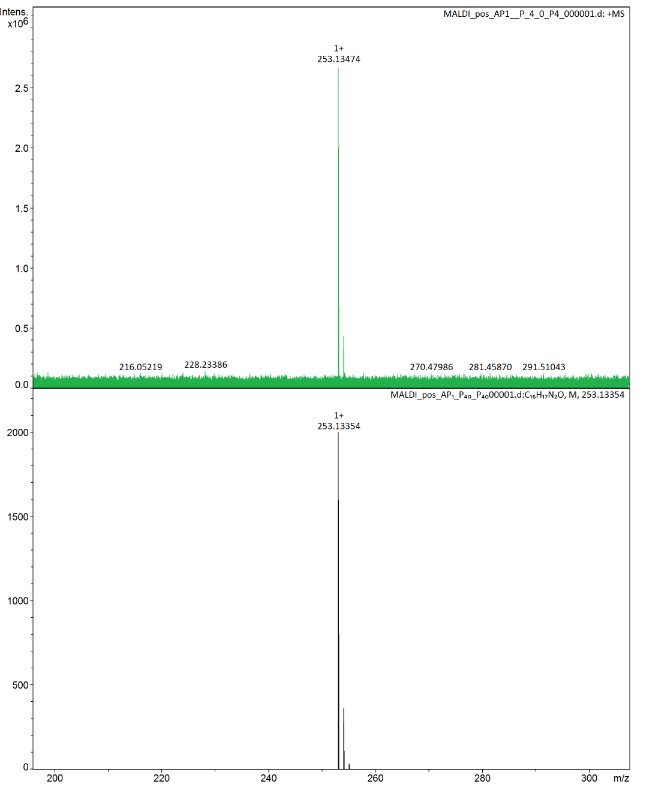


**MALDI-ToF** (m/z): 253.13474 Da attributable to the cationic portion of the imidazolium salt [C_16_H_17_N_2_O]^+^.

# ^1^H-NMR of 4a

**DMSO**


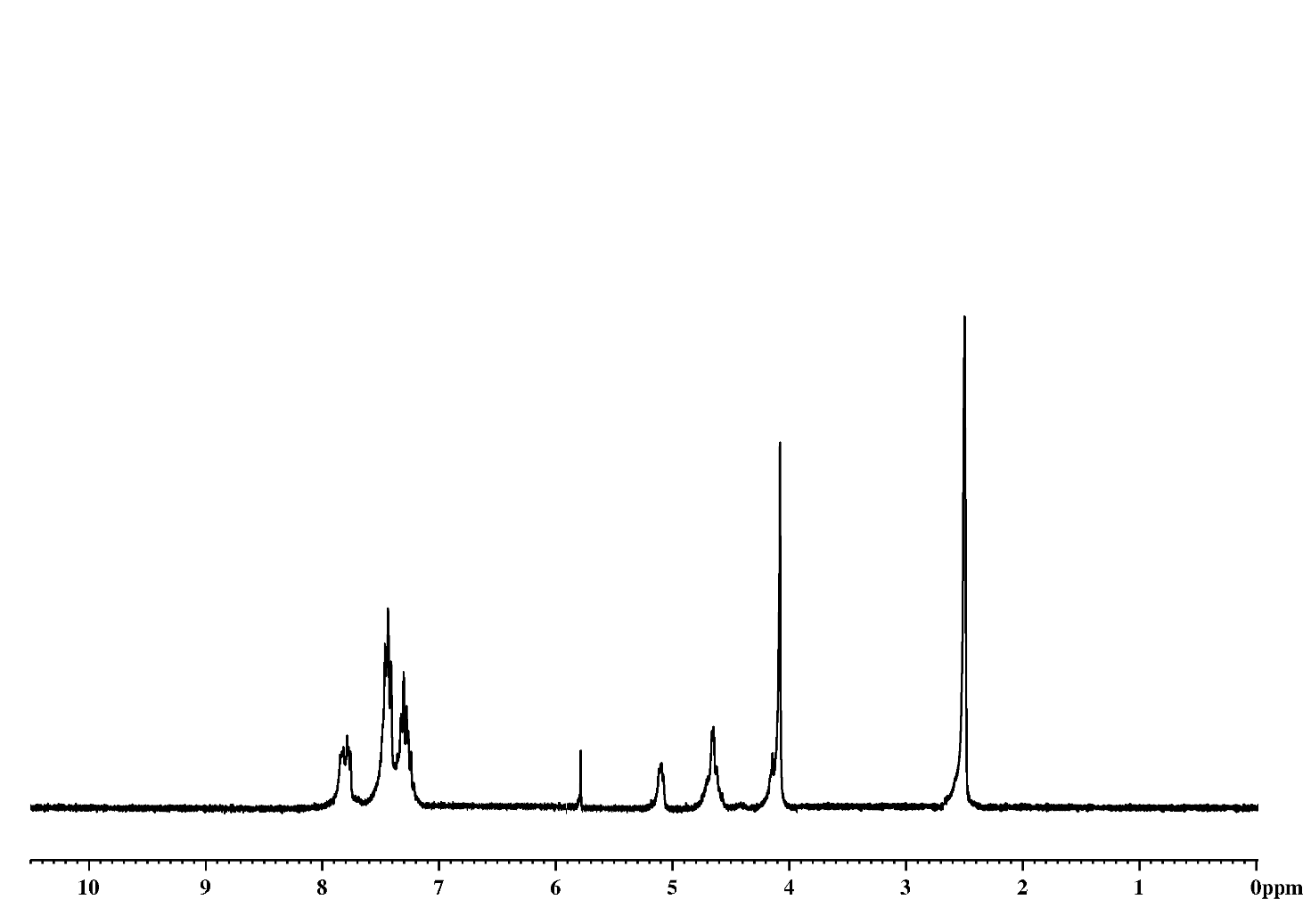


Ph-rings

OH

4

2, 3

1

**^1^H-NMR** (400 MHz, DMSO-d_6_): *δ* 7.82-7.24 (m, 9H, ***Ph rings***); 5.86 (s, 1H, O***H***); 5.09-5.07 (m, 1H, OC***H****,* *J_anti_ 7.85 Hz, J_gauche_ 5.40 Hz*); 4.65 (m, 2H, NC***H_2,_*** *J_gem_ 13.83 Hz, J_anti_ 7.85 Hz, J_gauche_ 5.40 Hz*); 4.04 (s, 3H, NC***H_3_***).

# ^13^C-NMR of 4a

**DMSO**


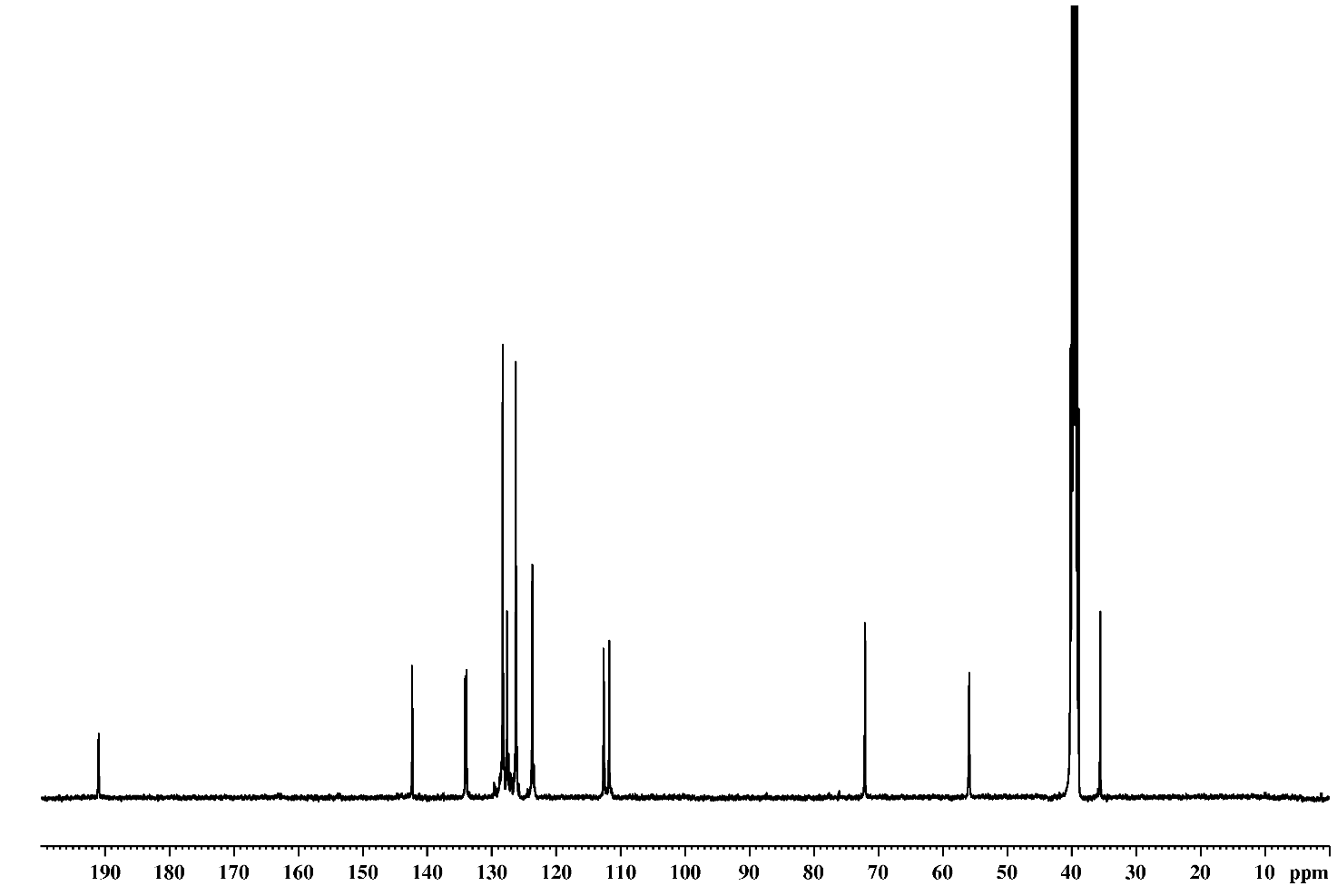


6

1’

5, 4

Ph-rings
2’ 3’ 4’ 7’ 6’

8’ 5’

3

2

1

**^13^C-NMR** (100 MHz, DMSO-d_6_): *δ* 190.9 (N***C***N); 142.3 *(ipso aromatic carbon*, ***Ph ring***); 134.4, 133.9 (*backbone carbons*, N***C***=***C***N); 128.2, 127.5, 126.2, 123.6 (*aromatic carbons*, ***Ph rings***); 112.6, 111.7 (*aromatic carbons*, ***Ph rings***); 72.0 (O***C***H); 55.8 (N***C***H_2_); 35.5 (N***C***H_3_).

# MALDI-MS of 4a


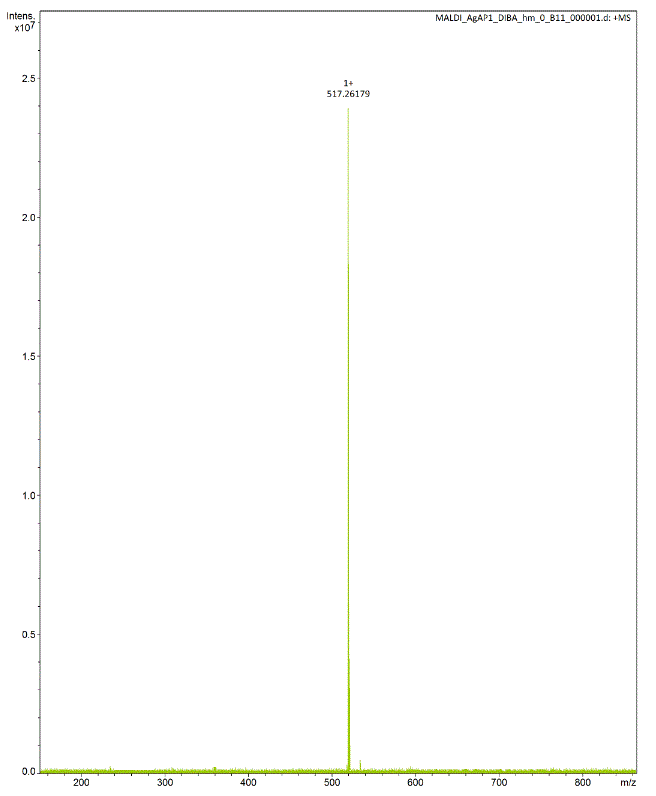


**MALDI-ToF (m/z):** 517.26179 Da attributable to bis-carbene structure [C_26_H_25_AgN_4_O]^+^.

# ^1^H-NMR of 4b

**DMSO**


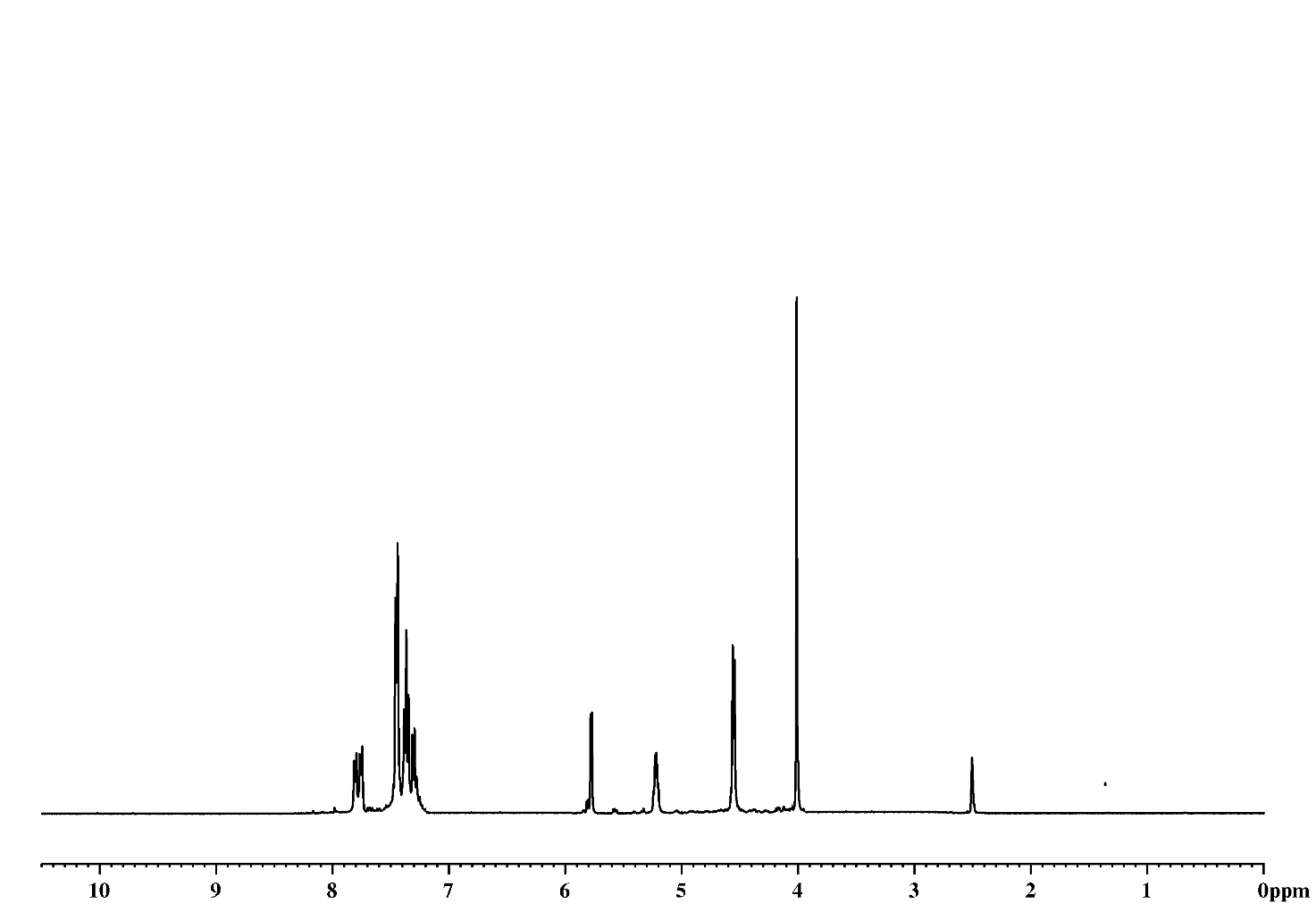


1

2, 3

4

OH

Ph rings

**^1^H-NMR** (300 MHz, DMSO-d_6_): *δ* 7.80-7.28 (m, 9H, ***Ph rings***); 5.76 (s, 1H, O***H***); 5.22 (m, 1H, OC***H,*** *J_anti_ 7.68 Hz, J_gauche_ 5.89 Hz*); 4.60 (m, 2H, NC***H_2_,*** *J_gem_ 14.65 Hz, J_anti_ 7.68 Hz, J_gauche_ 5.89 Hz*); 4.00 (s, 3H, NC***H_3_***).

# ^13^C-NMR of 4b

**DMSO**


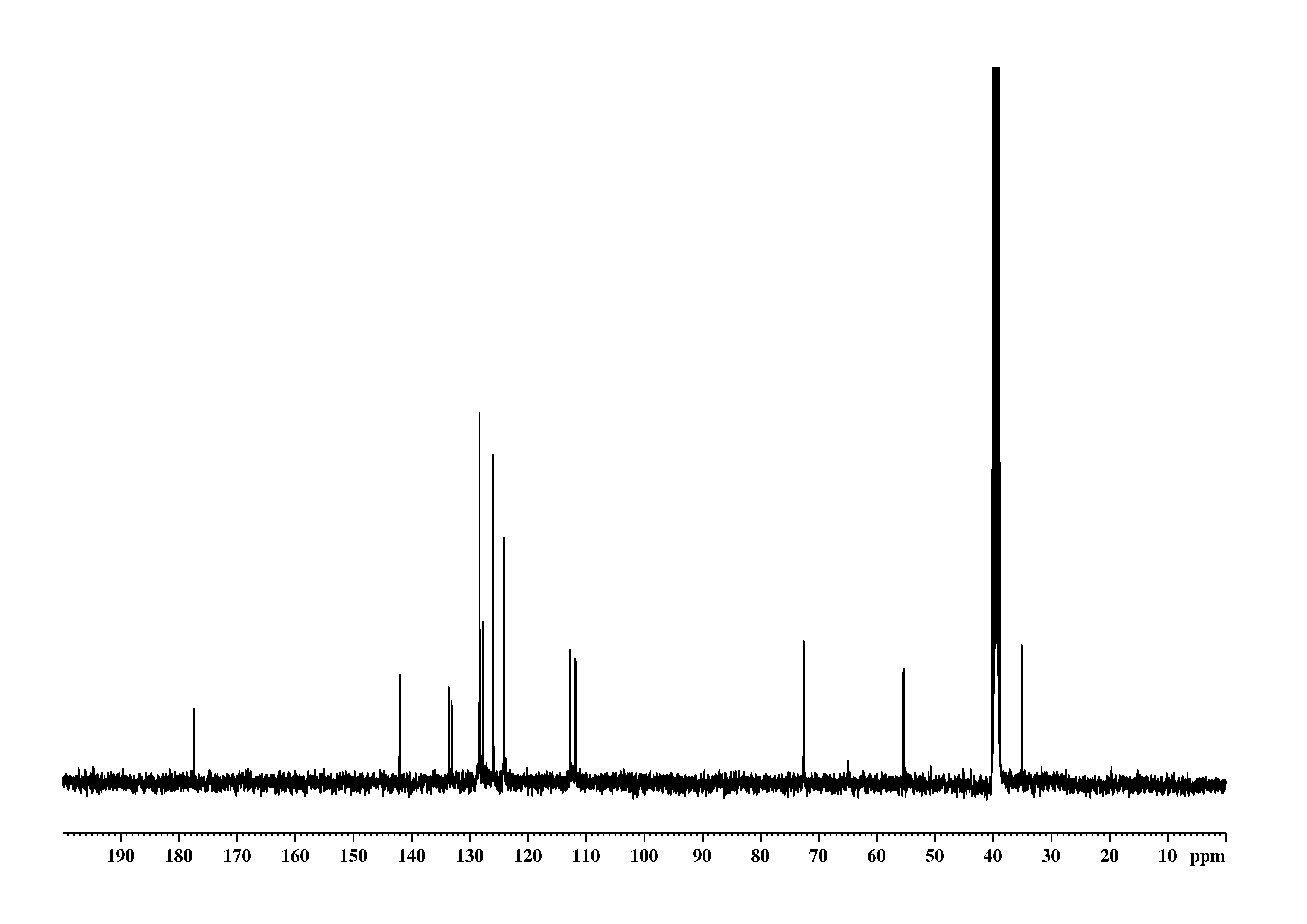


6

1’

4,5

Ph rings

8’,5’

3

2

1

**^13^C-NMR** (75 MHz, DMSO-d_6_): *δ* 177.4 (N***C***N); 141.9 *(ipso aromatic carbon*, ***Ph ring***); 133.5, 133.1 (*backbone carbons*, N***C***=***C***N); 128.3, 127.7, 126.0, 124.1 (*aromatic carbons*, ***Ph rings***); 112.8, 111.8 (*aromatic carbons*, ***Ph rings***); 72.5 (O***C***H); 55.4 (N***C***H_2_); 35.0 (N***C***H_3_).

# MALDI-ToF of 4b

**MALDI- ToF (m/z):** 701.22194 Da attributable to bis-carbene structure [C_32_H_32_AuN_4_O_2_]^+^

+


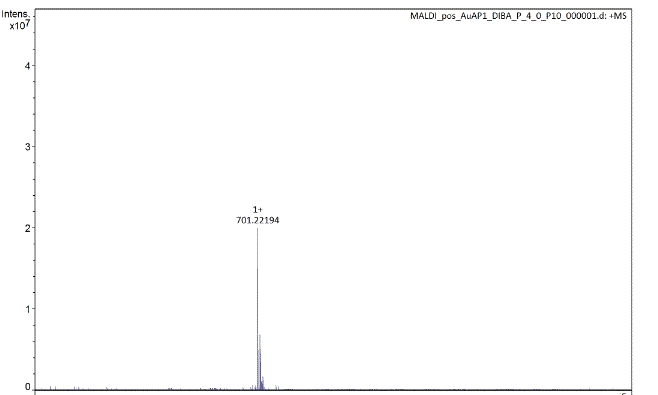


# ^1^H-NMR of 5b

1


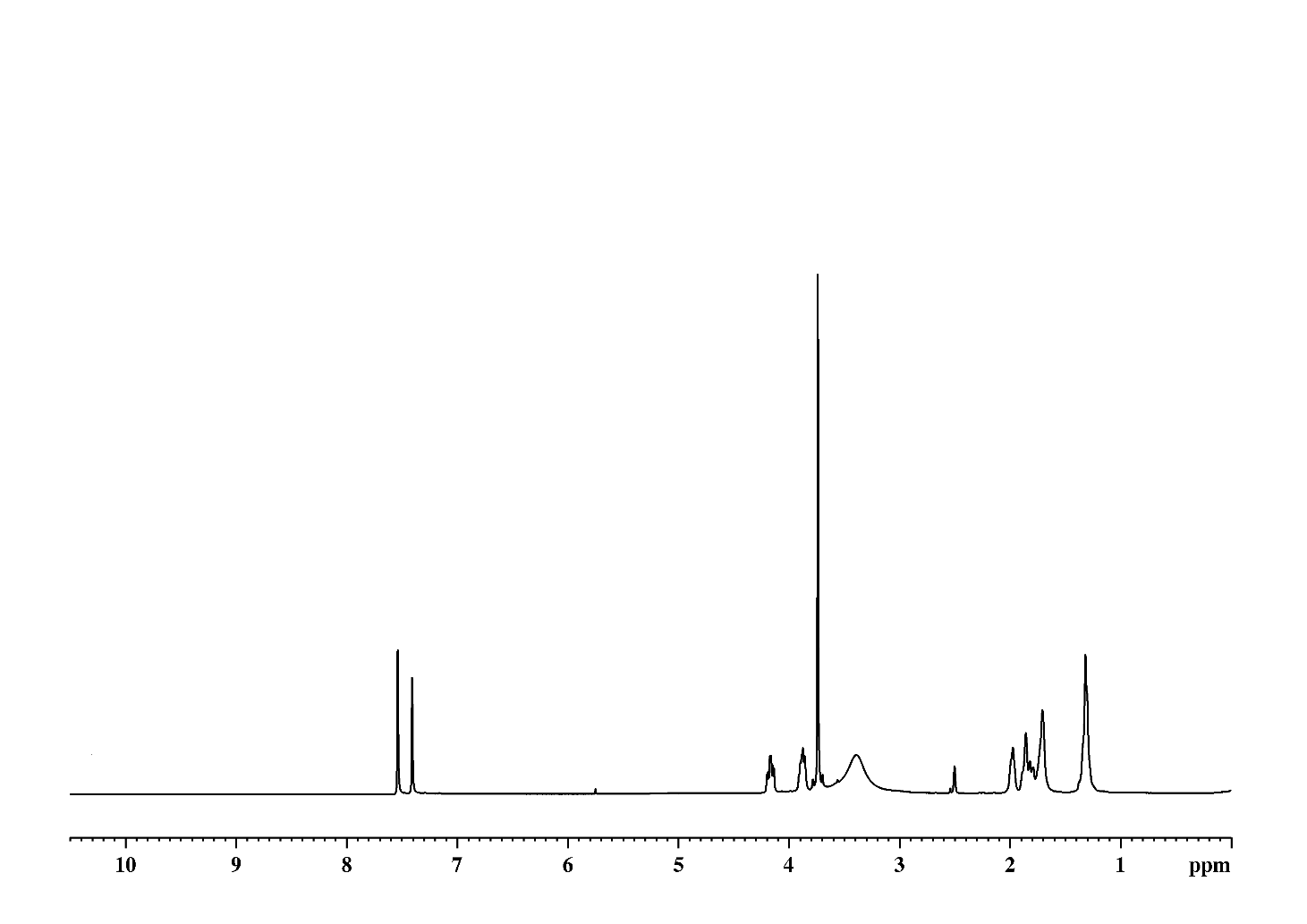


H -cyclohexyl group

**DMSO**

H_2_O

2,3

6, 5

**^1^H-NMR** (400 MHz, DMSO-d_6_): *δ* 7.01-6.99 (s, 2H, NC***H***C***H***N); 5.48 (s, 1H, O***H***CH); 4.48 (m, 1H, OC***H***); 3.62 (m, 1H, NC***H***); 3.69 (s, 3H, C***H_3_***); 2.29-1.40 (m, cyclohexyl group, 8H)

# ^13^C-NMR of 5b

DMSO


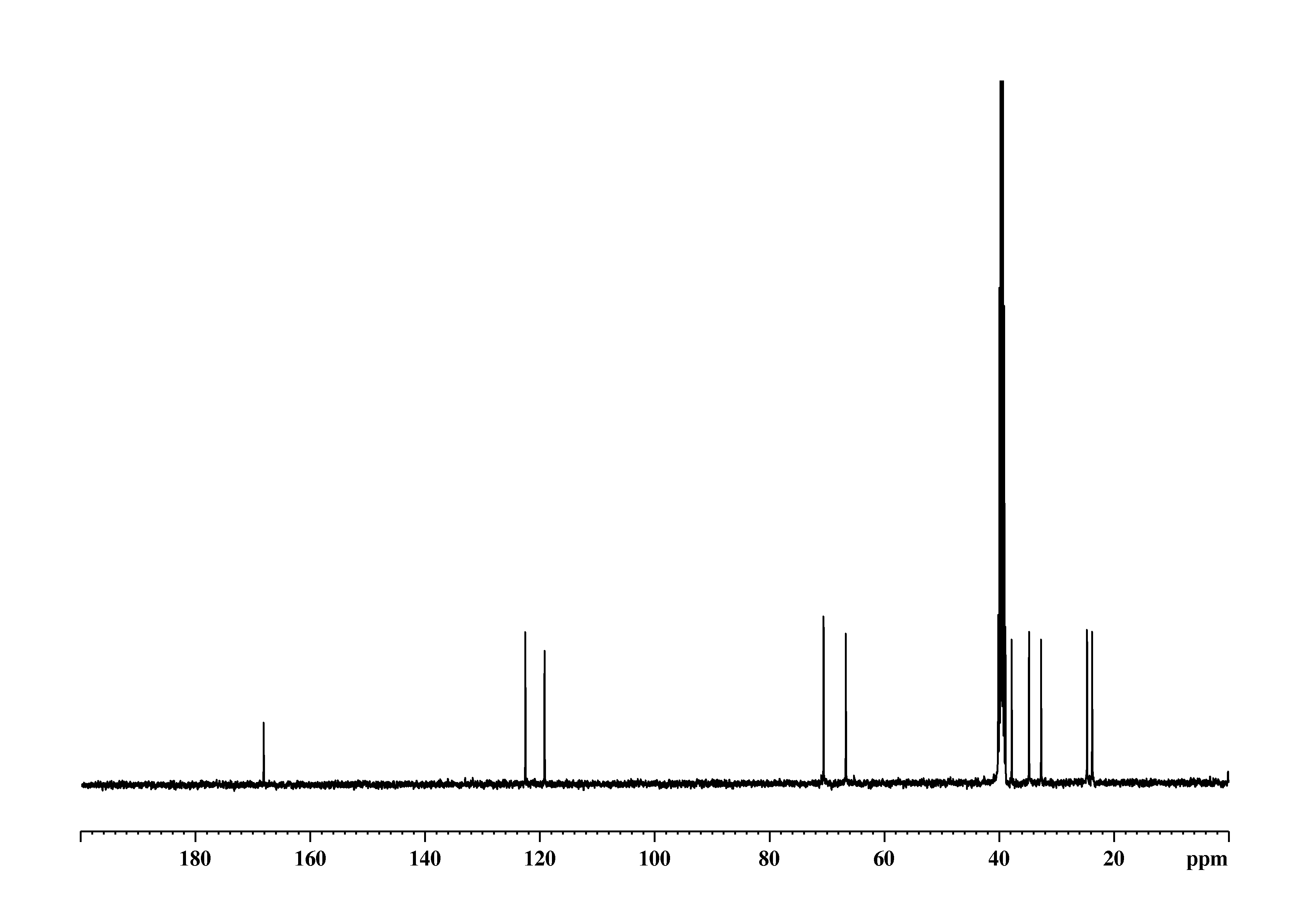


1

6, 5

7

2, 3

C -cyclohexyl group

**^13^C-NMR** (100 MHz, DMSO-d_6_): *δ* 178.9 (N***C***N); 131.5-126.9 (*backbone carbons,* N***C***H=***C***HN); 72.9 (O***C***H); 67.4 (N***C***H); 43.8 (N**C**H_3_); 41.9 (OCH***C***H_2_); 35.1, 33.2, 26.8 (cyclohexyl group).

**MALDI- ToF (m/z):** 557.2191 attributable to bis carbene structure [C_20_H_32_AuN_4_O_2_]^+^

# ^1^H-NMR of PL-6

**DMSO**


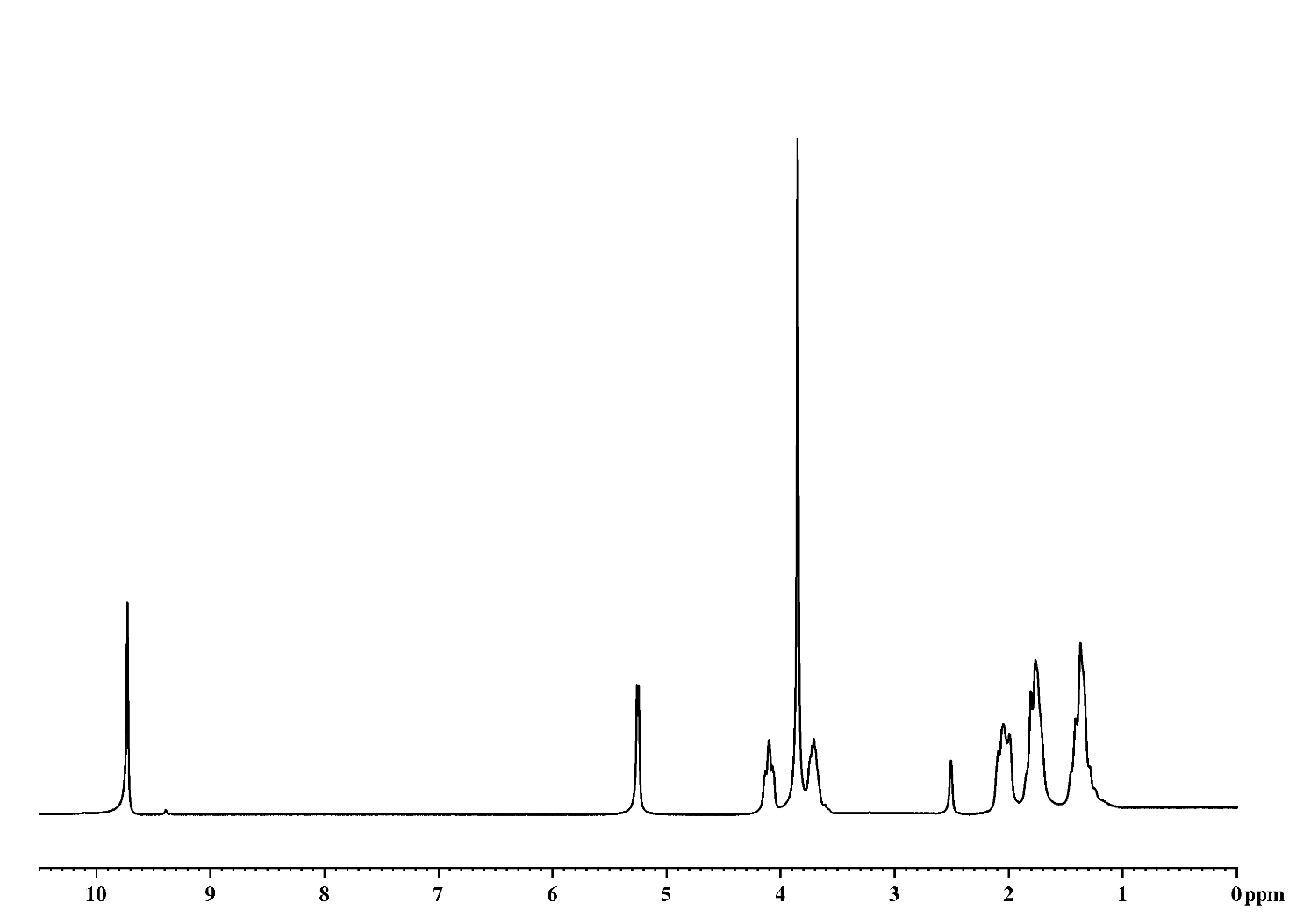


12

OH-group

2

1

11

Cyclohexyl group

3, 4, 5, 6, 7, 8, 9, 10

**^1^H-NMR** (300 MHz, DMSO-d_6_): *δ* 9.72 (s, 1H, NC***H***N); 5.25 (m, 1H, O***H***); 4.09 (m, 1H, OC***H,*** *J_ax-eq_ 4.81 Hz, J_ax-eq_ 4.50 Hz, J_eq-eq_ 2.8Hz*); 3.84 (s, 3H, NC***H_3_***); 3.70 (m, 1H, NC***H,*** *J_ax-ax_ 11.5 Hz, J_ax-eq_ 4.81 Hz, J_eq-eq_ 2.7 Hz*); 2.08-1.36 (m, 8H, ***Cyclohexyl protons***).

# ^13^C-NMR of PL-6

**DMSO**


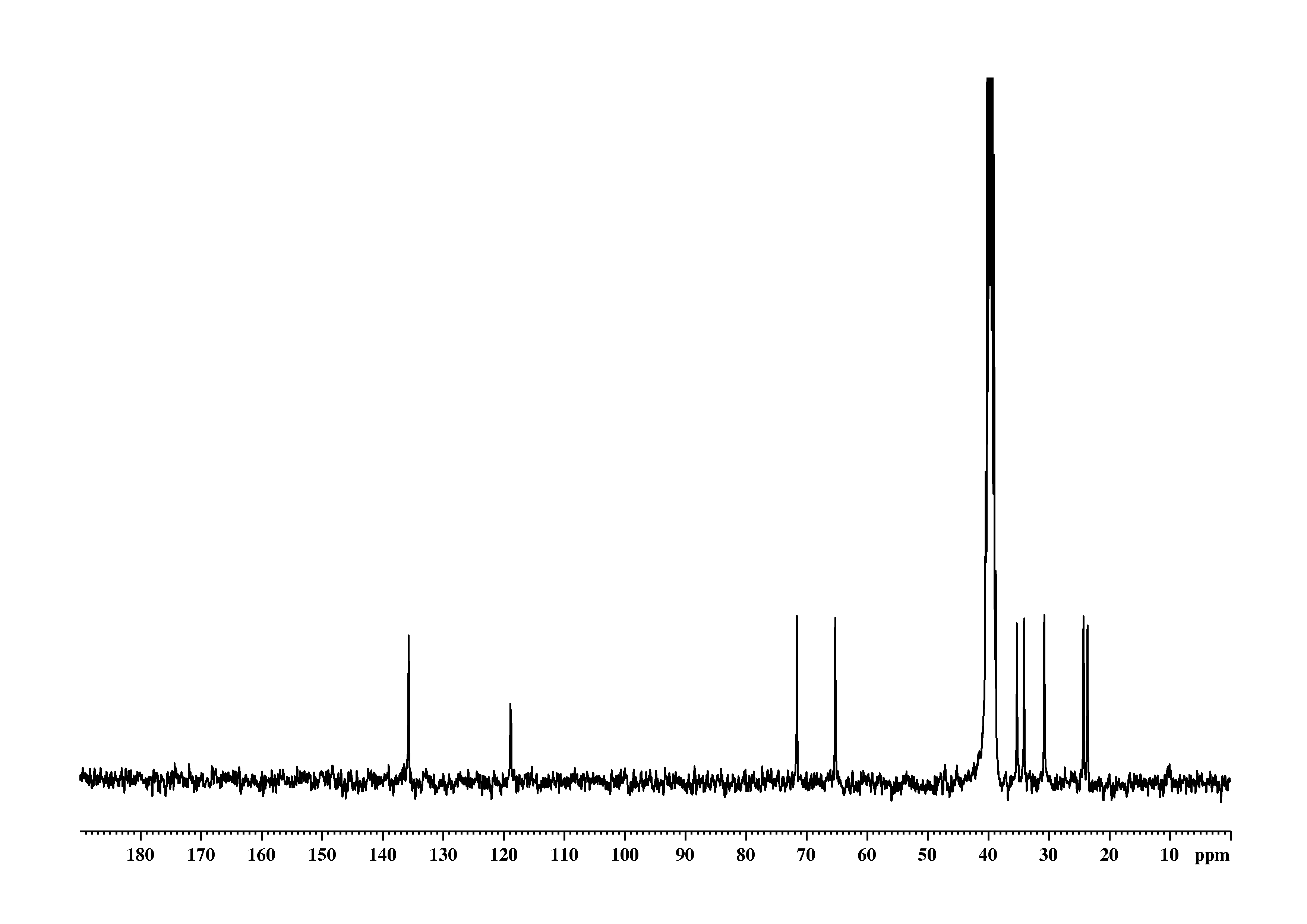


10

8,7

2

1

3,4,5,6

9

**^13^C-NMR** (75 MHz, DMSO-d_6_): *δ* 135.6 (N***C***N); 118.8 (*backbone carbons*, N***C***Cl=***C***ClN); 71.5 (O***C***H); 65.2 (N***C***H); 35.2 (N***C***H_3_); 34.0, 30.68, 24.2, 23.5 (***Cyclohexyl carbons***).

# MALDI-ToF of PL-6


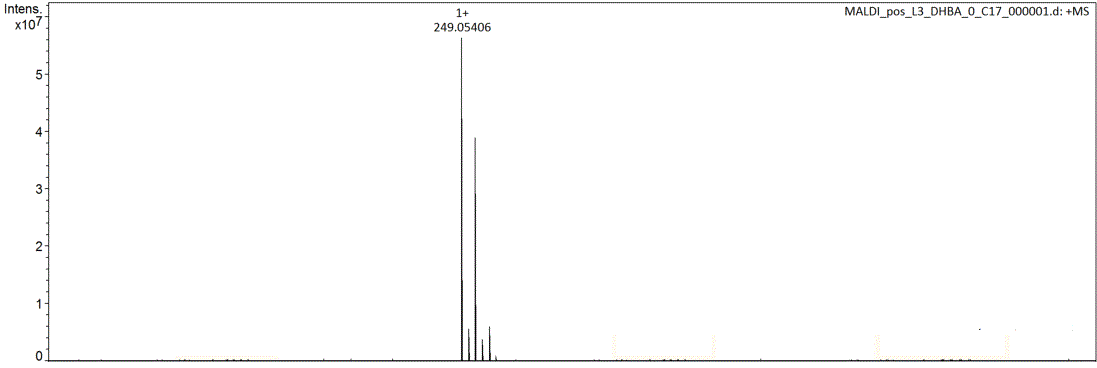


**MALDI-ToF (m/z):** 249.05406 Da attributable to the cationic portion of the imidazolium salt [C_10_H_15_Cl_2_N_2_O]^+^.

# ^1^H-NMR of 6a

**DMSO**


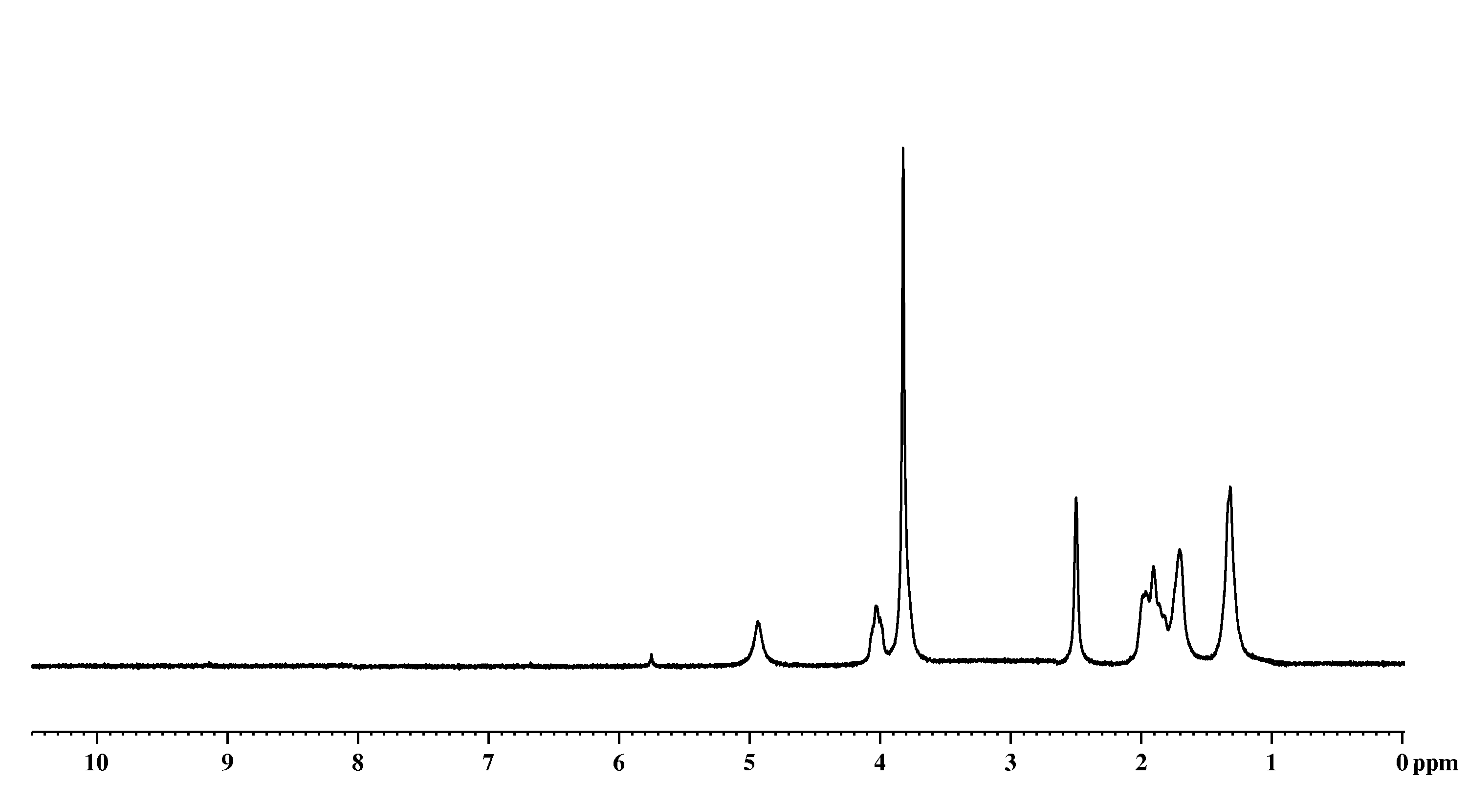


OH

3

1,2

Cyclohexyl group

4, 5, 6, 7, 8, 9, 10, 11

**^1^H-NMR** (400 MHz, DMSO-d_6_): *δ* 4.93 (b, 1H, O***H***); 4.00 (m, 1H, HOC***H*** *J_ax-eq_ 4.81 Hz, J_ax-eq_ 4.50 Hz, J_eq-eq_ 2.8Hz*); 3.86 (o, 4H, NC***H***, NC***H_3_***); 1.96-1.31 (m, 8H, ***Cyclohexyl protons***).

# ^13^C-NMR of 6a

**DMSO**


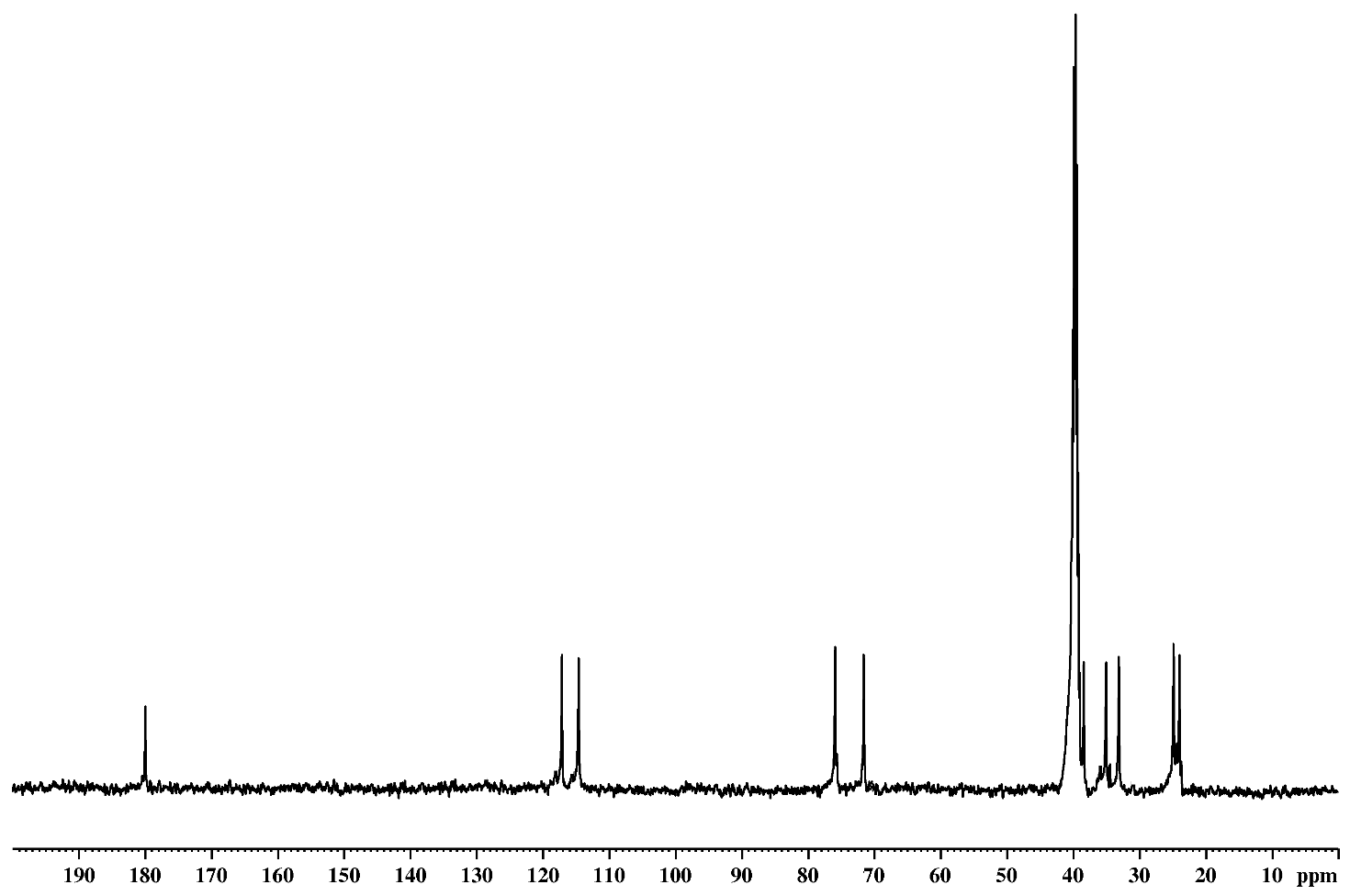


10

8,9

3, 2

1

Cyclohexyl

4, 5, 6, 7

**^13^C-NMR** (100 MHz, DMSO-d_6_)**:** *δ* 182.9 (N***C***N); 118.6, 117.3 (***backbone carbons,*** N***C***Cl=***C***ClN); 76.1 (***C***HOH); 70.4 (N***C***H); 38.8 (N***C***H_3_); 35.0, 30.7, 24.2, 23.9 (***Cyclohexyl carbons***).

# MALDI-ToF of 6a


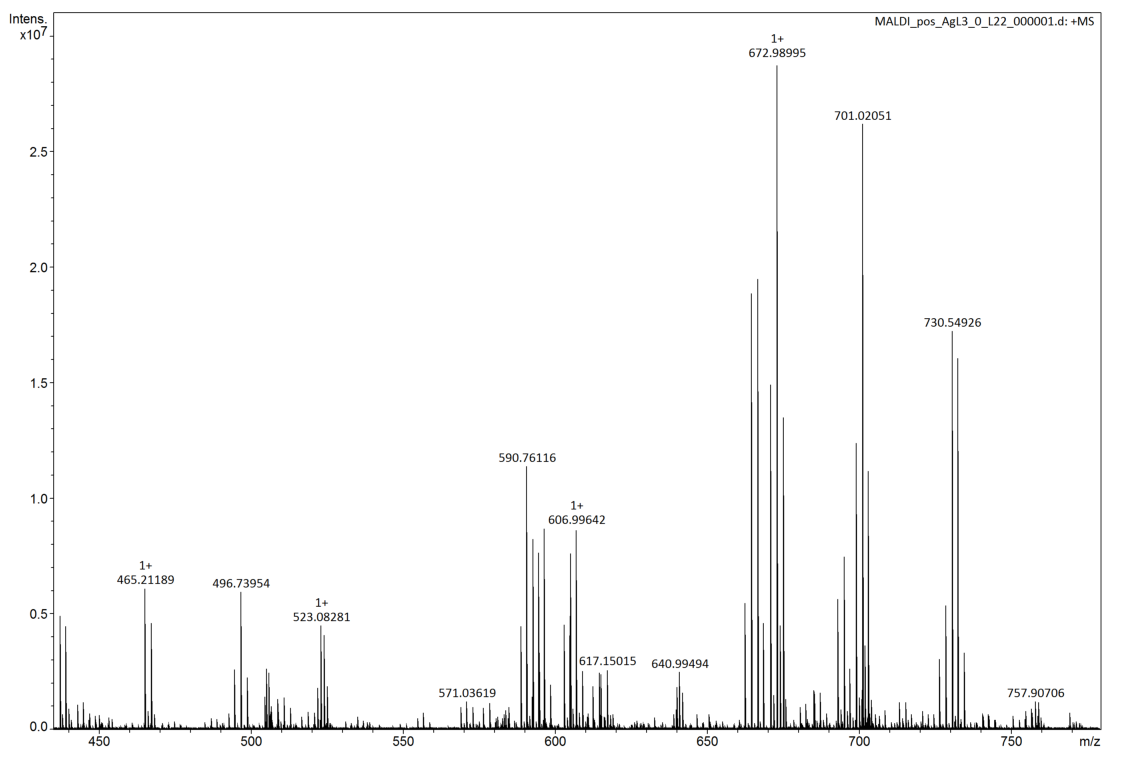

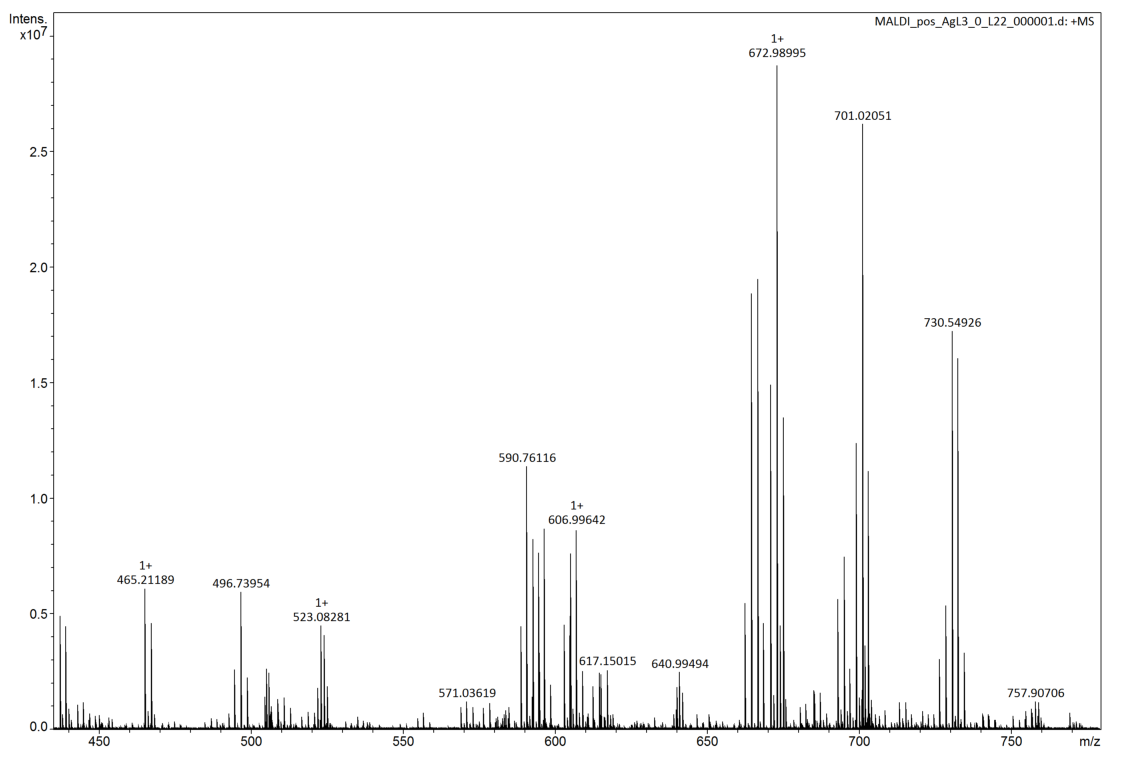

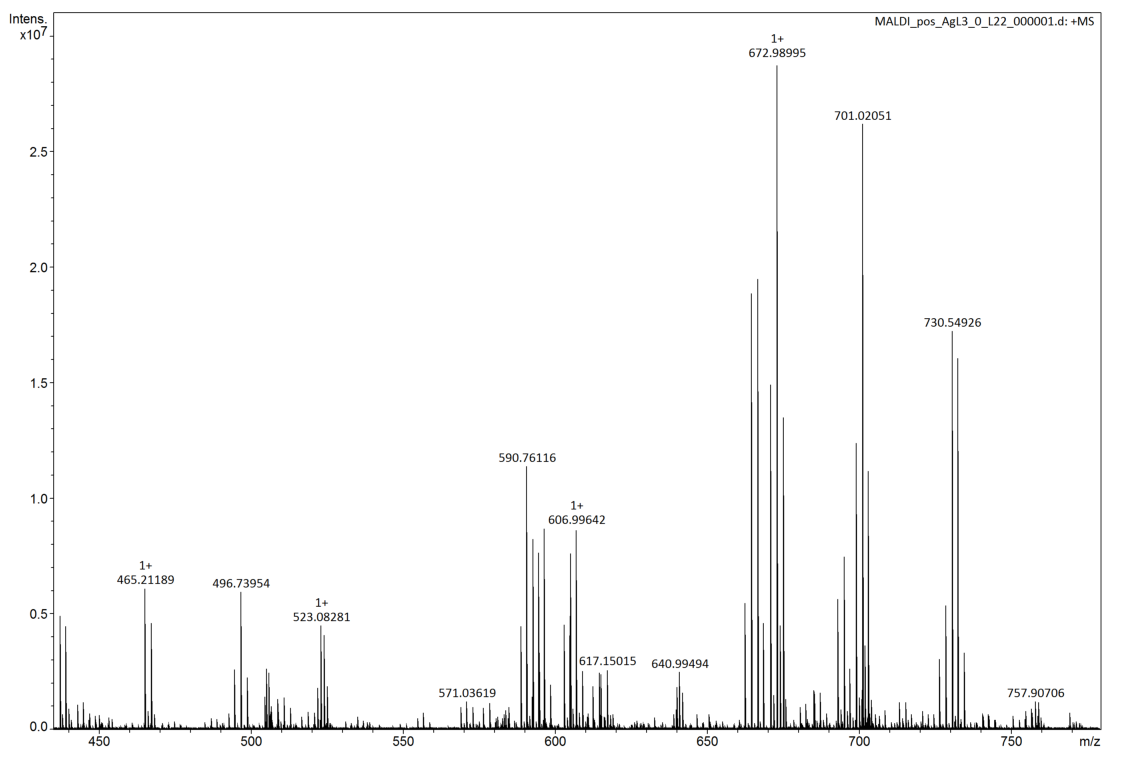


**MALDI-ToF (m/z):** 606.99642 Da attributable to bis carbene strucuture [C_20_H_28_AgCl_4_N_4_O_2_]^+^.

# ^1^H-NMR of 6b

**DMSO**


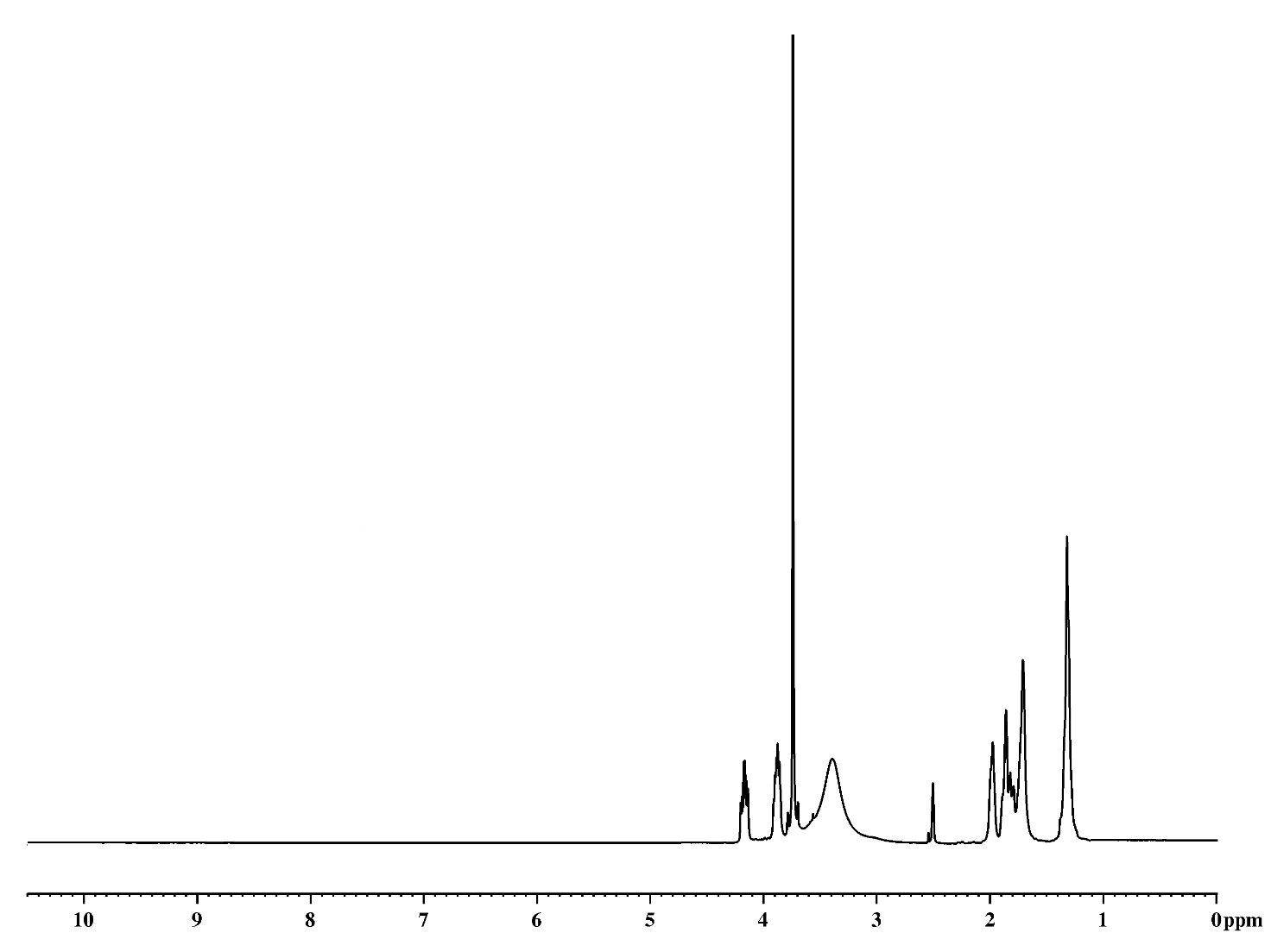


3

2

1

Cyclohexyl group

*4, 5, 6, 7, 8, 9, 10, 11*

*H_2_O*

**^1^H-NMR** (400 MHz, DMSO-d_6_): *δ* 4.17-4.12 (m, 1H, HOC***H***); 3.89-3.86 (m, 1H, NC***H***); 3.73 (s, 3H, NC***H_3_***); 1.97-1.29 (***Cyclohexyl* *protons***).

# ^13^C-NMR of 6b


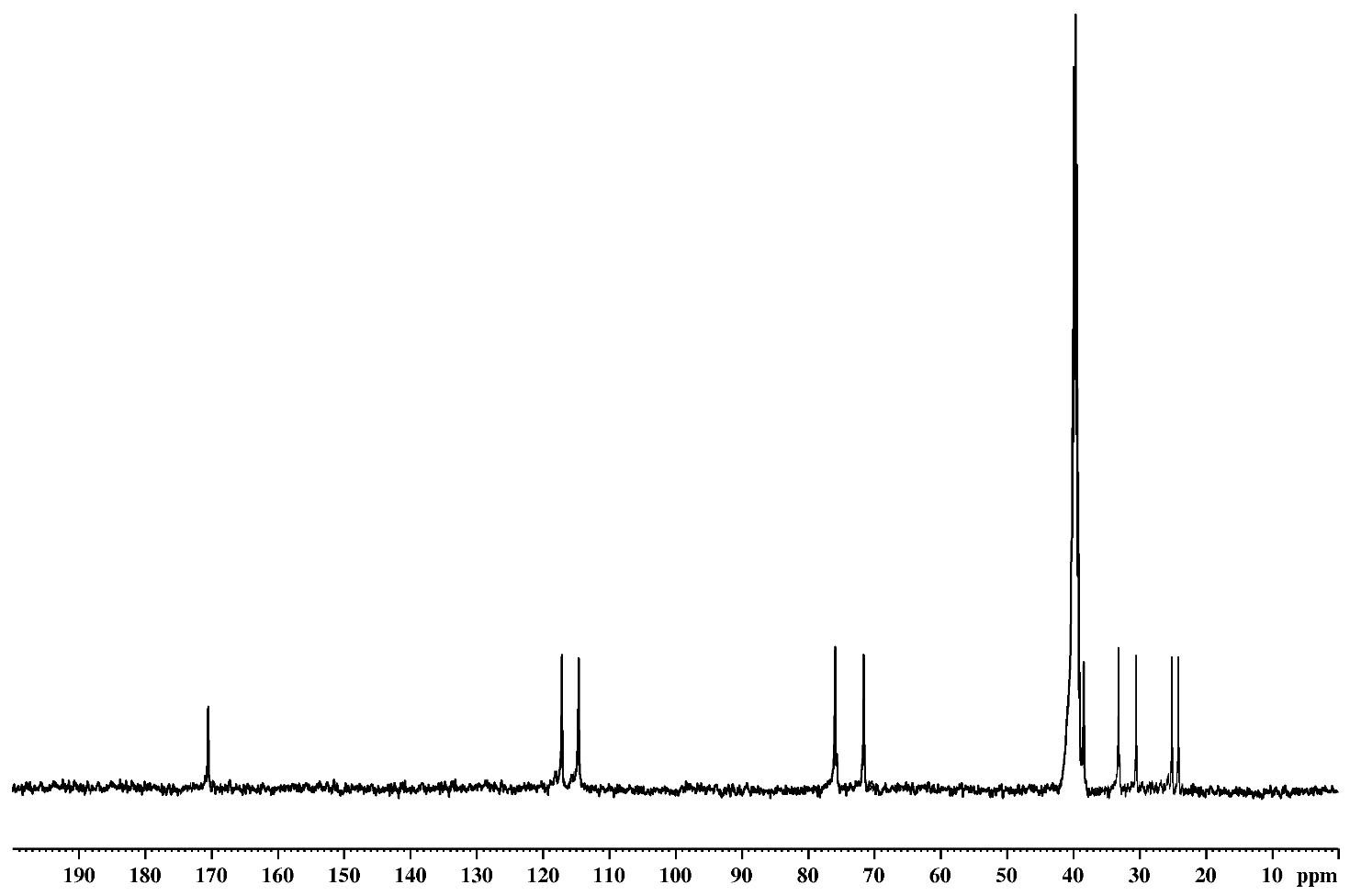


10

9 8

3 2

*1*

Cyclohexyl

**DMSO**

**^13^C-NMR** (100 MHz, DMSO-d_6_)**:** *δ* 171.2 (N***C***N); 119.6, 117.2 (*backbone carbons,* N***C***Cl=***C***ClN); 77.1 (***C***HOH); 71.4 (N***C***H); 39.1 (N***C***H_3_); 35.1, 30.6, 24.3, 23.8 (***Cyclohexyl carbons***).

# MALDI-ToF of 6b


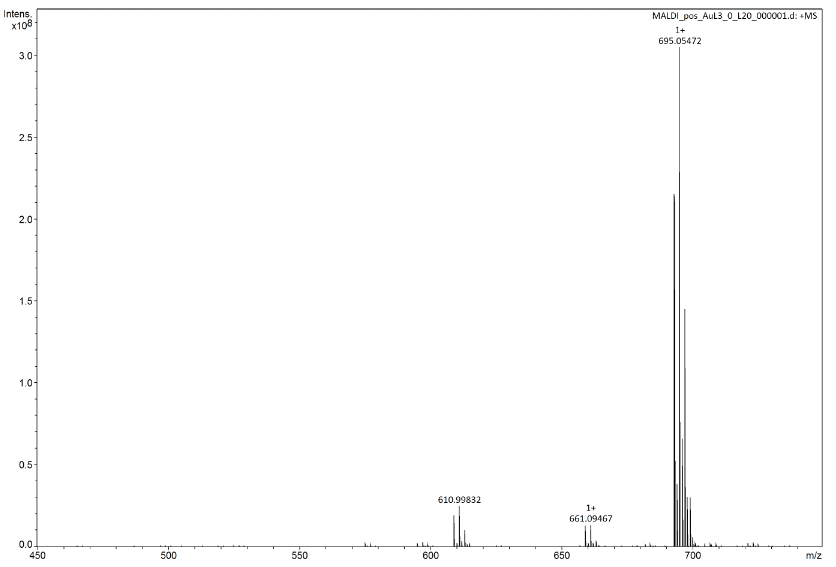


**MALDI- ToF:** 695.05472 Da attributable to [C_20_H_28_AuCl_4_N_4_O_2_]^+^

# ^1^H-NMR of 7b

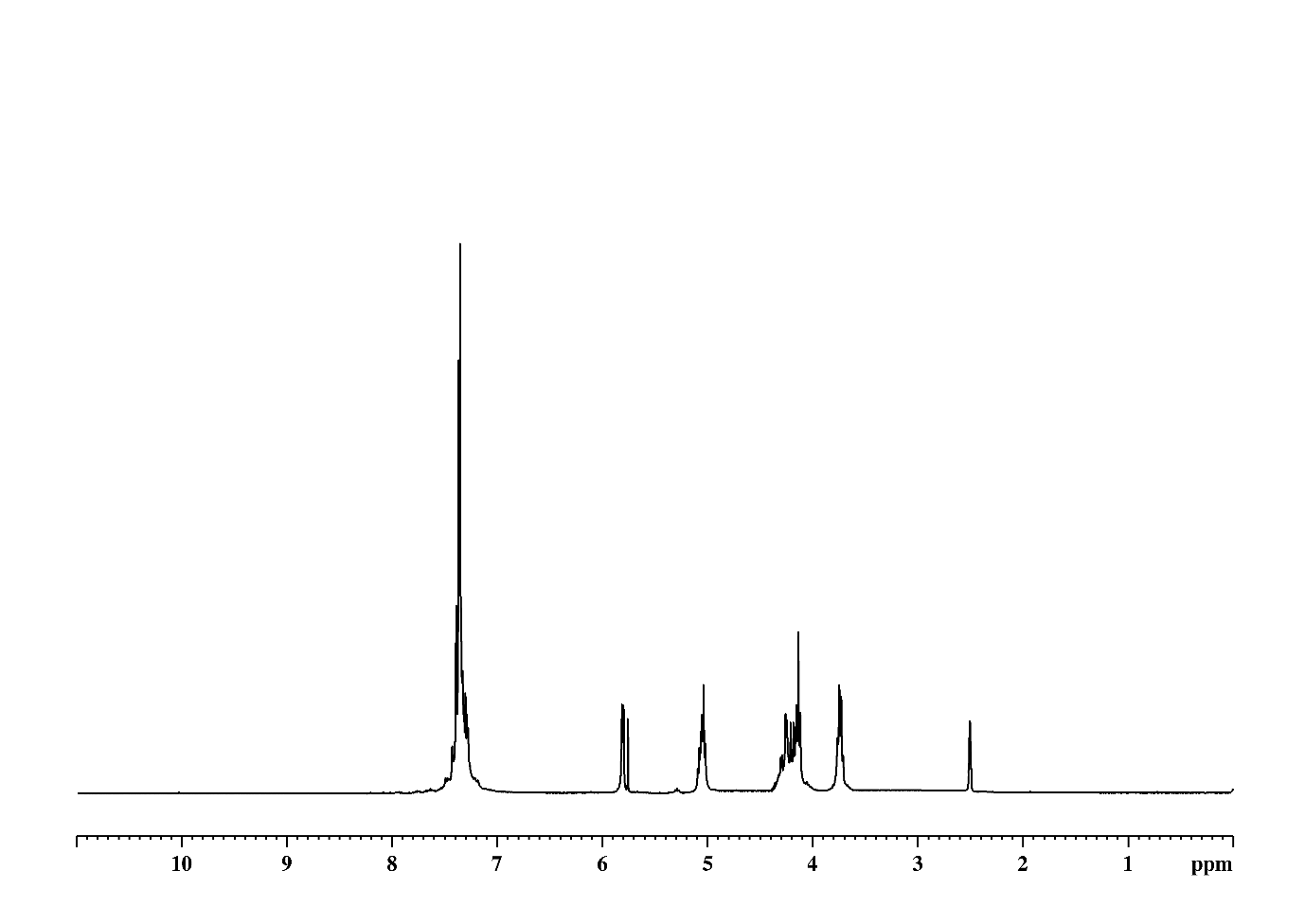


3, 1

Ph ring

OH group

4

2

**DMSO**

**^1^H-NMR** (400 MHz, DMSO-d_6_) *δ*: 7.40-7.36 (m, 7H, ***Ph ring*** and ***backbone protons***); 5.81 (m; C***H***OH; 1H); 5.08-5.02 (m, NC***H***_2_CH_2_OH; NC***H***_2_CHOH, 4H); 3.74 (d, NC***H***_2_CH_2_, 2H).

# ^13^C-NMR of 7b

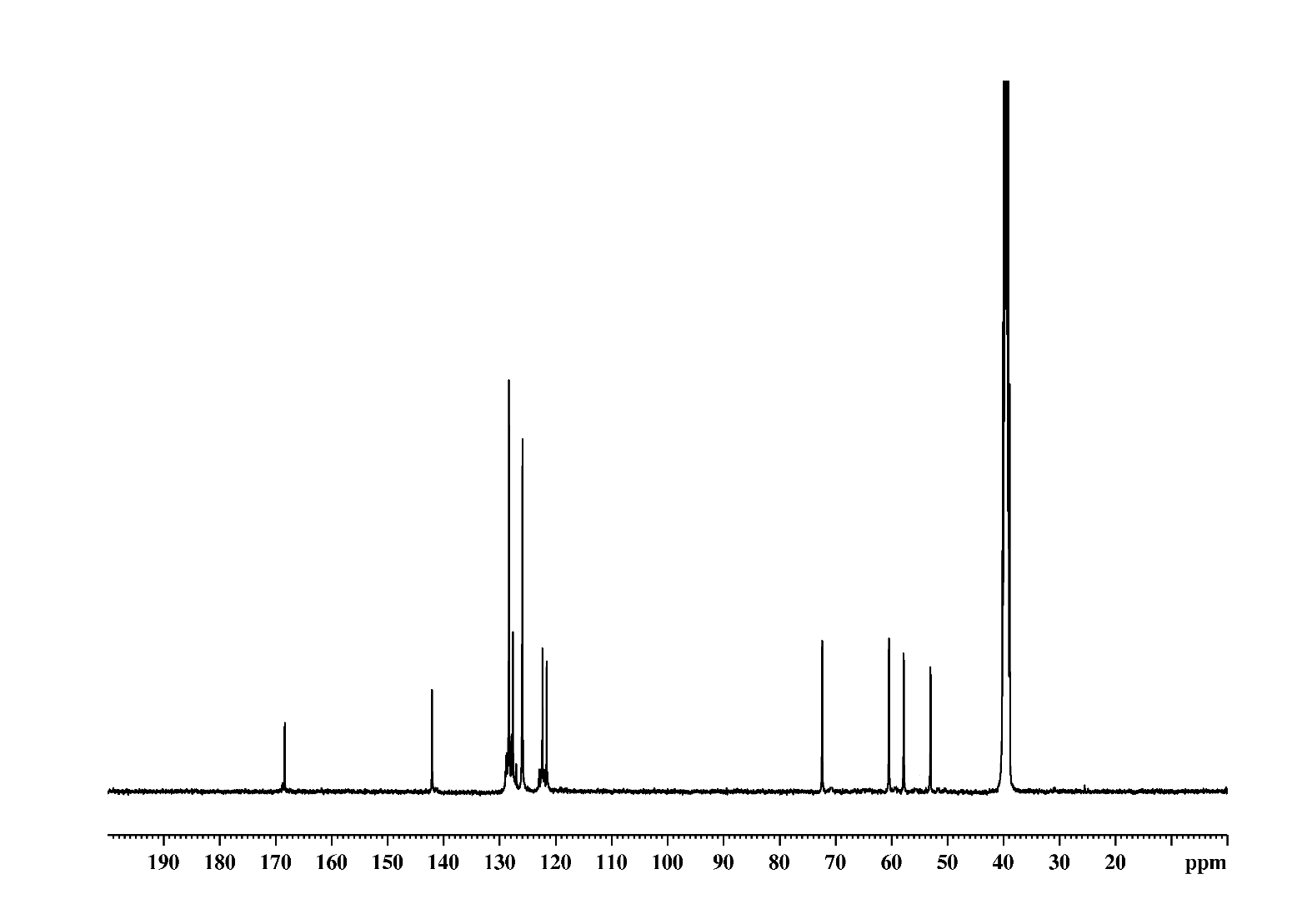


Ph ring

**DMSO**

1 3 2

4

5, 6

7

**^13^C-NMR** (100 MHz, DMSO-d_6_): *δ* 168.37 (N***C***N); 142.1 (*ipso* carbon aromatic ring, ***Ph ring***); 128.3, 127.8, 127.6 (*aromatic carbons*, ***Ph ring***); 122.3, 121.6 (*backbone carbons,* NH***C***=***C***HN); 72.4 (***C***HOH); 60.4 (***C***H_2_OH); 57.8 (N***C***H_2_CHOH); 53.0 (N***C***H_2_CH_2_OH).

**MALDI-TOF (m/z):** 661.20838 attributable to bis carbene structure [C_26_H_32_AuN_4_O_4_]^+^

# ^1^H-NMR of 8b

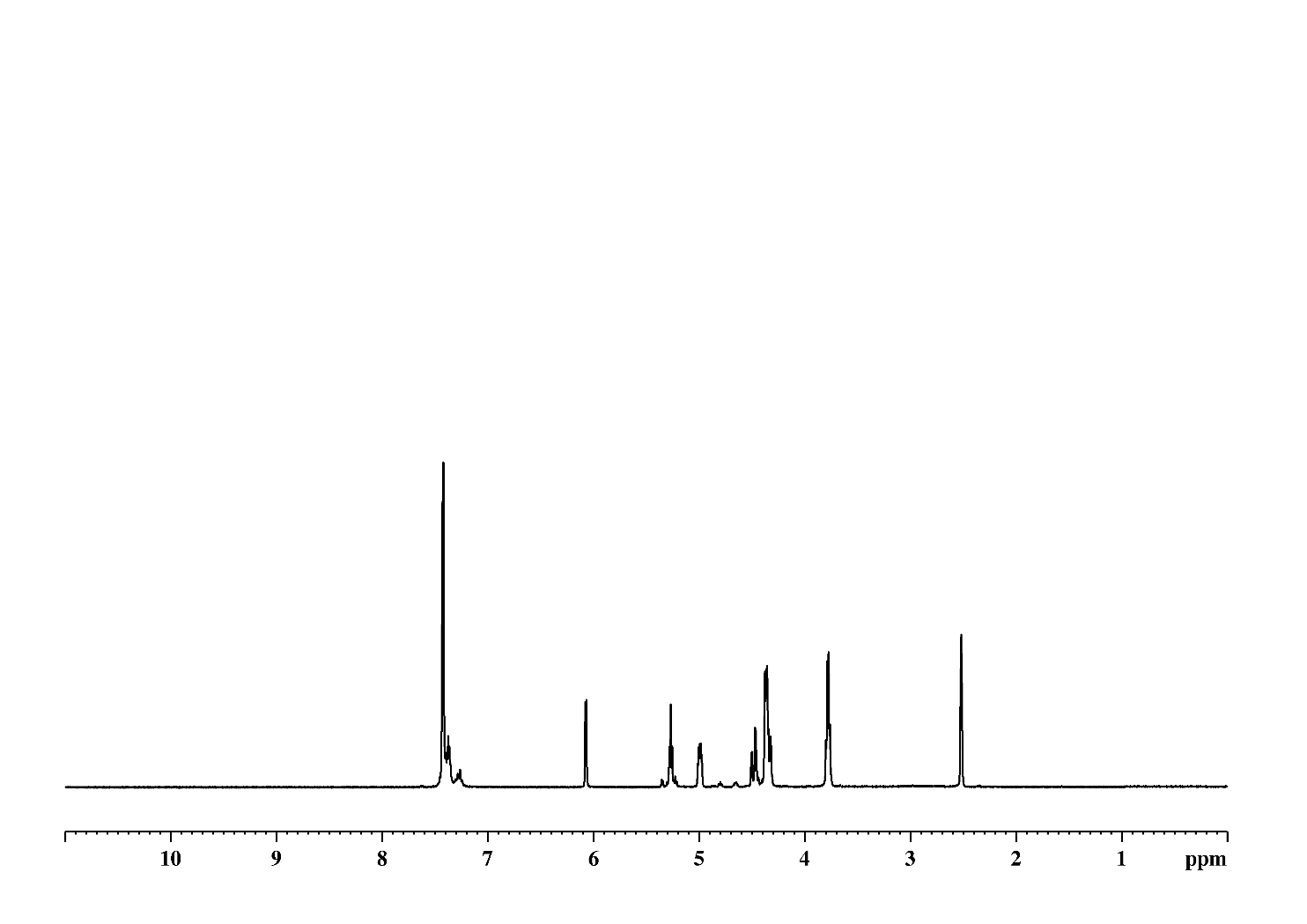


**DMSO**

4

1, 3

2

Ph ring

OH

OH

**^1^H-NMR** (400 MHz, DMSO-d_6_): *δ* 7.38-7.27 (m, 5H, **Ph ring**); 6.08 (s, 1H, O***H***); 5.07 (s, 1H, O***H***); 4.99 (m, 1H, OC***H***); 4.33-4.27 (m, 4H, NCH_2_C***H_2_***OH, NC***H*_2_**CHOH); 3.74 (s, 2H, NC***H_2_***CH_2_OH).

# ^13^C-NMR of 8b

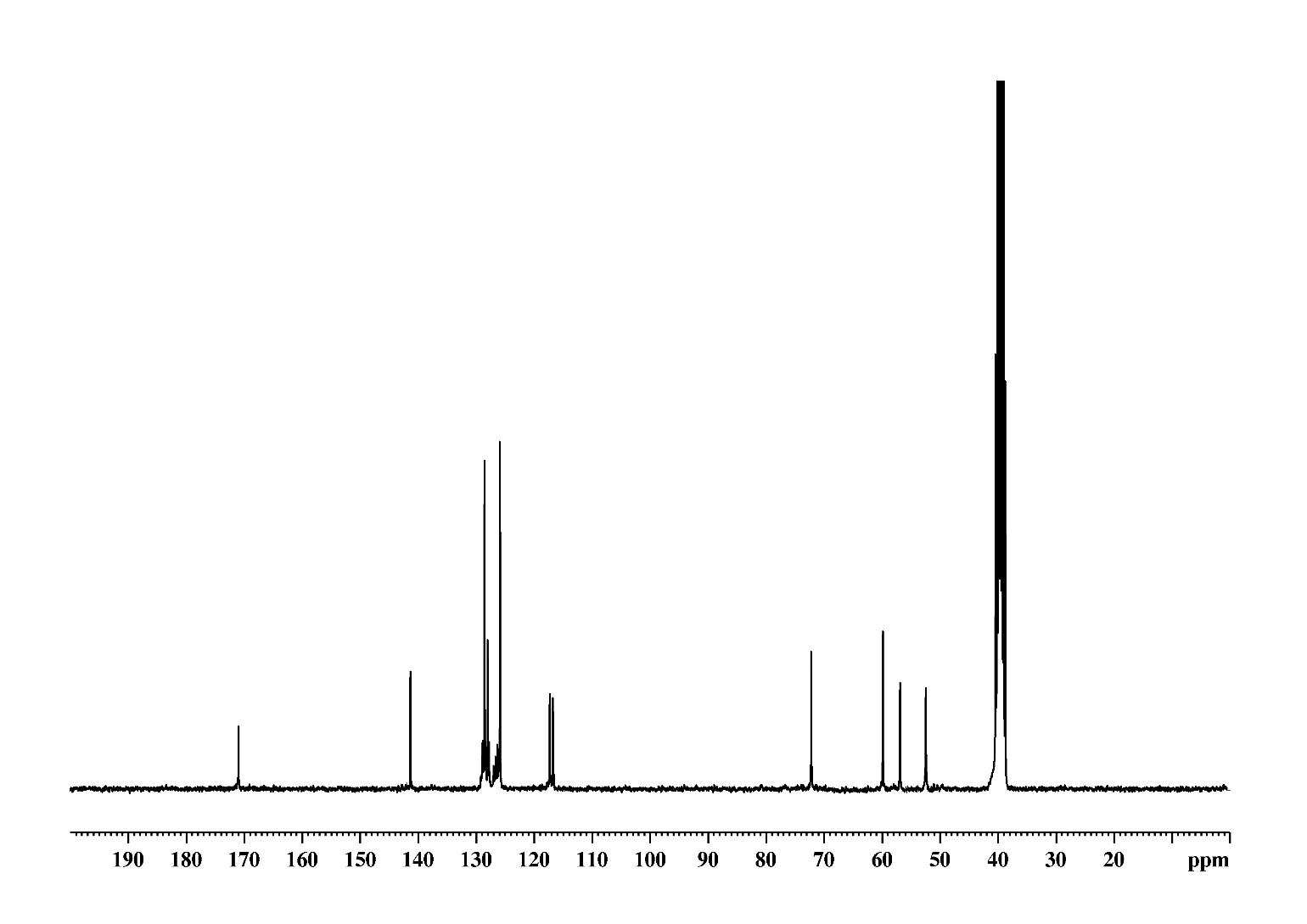


3 2

1

4

5, 6

Ph group

7

**^13^C-NMR** (100 MHz, DMSO-d_6_): *δ* 170.9 (N***C***N); 141.3 (*ipso aromatic carbon,* ***Ph ring***); 128.4, 127.9, 125.1 (*aromatic carbons,* ***Ph ring***); 117.2, 116.7 (*backbone carbons,* NH***C***=***C***HN); 72.1 (***C***HOH); 59.9 (***C***H_2_OH); 56.9 (N***C***H_2_CHOH); 52.4 (N***C***H_2_CH_2_OH).

**MALDI-TOF (m/z):** 707.99716 attributable to bis carbene structure [C_26_H_32_AgN_4_O_4_]^+^

# Computational details

The DFT calculations were performed with the Gaussian09 set of programs ^[1]^, using the PBE0 model^[2]^. The electronic configuration of the molecular systems was described with 6-311G(d,p) basis set for H, C, N, O, and Cl^[3,4]^. For Au we used the Stuttgart/Dresden ECP (effective core potential) (standard SDD keyword in gaussian09)^[5–8]^. The geometry optimizations were performed without symmetry constraints and the characterization of the located stationary points and transition states was performed by analytical frequency calculations. Improved electronic energies were obtained by single-point calculations, on the gas phase optimized structures, using solvent effects including contributions of non electrostatic terms, based on the polarizable continuous solvation model PCM using CH_2_Cl_2_ as a solvent^[9,10]^. Free energies reported herein were obtained by adding these energies to the free energy thermal corrections of the PBE0/6-311G(d,p) optimization level.

# Cartesian coordinates and energies of calculated structures.

36

**a** E(gas)=-1836.94421719 E(CH3CN)=-1837.006367 G(CH3CN)=-1836.79088

Au 1.680303 -0.797973 -0.110907

C 0.870007 1.025232 -0.135322

N -0.427719 1.324292 -0.357095

N 1.492731 2.199113 0.095526

C -0.621422 2.685929 -0.253177

C 0.587567 3.236736 0.028208

C 2.908916 2.371468 0.371601

H 3.358952 3.016620 -0.384124

H 3.044689 2.813872 1.359464

H 3.377081 1.389260 0.341523

C -1.488314 0.349618 -0.586547

C -2.216125 -0.039508 0.698274

H -2.190453 0.766405 -1.309176

H -1.025637 -0.536274 -1.025548

C -3.359232 -0.972194 0.370294

O -1.237400 -0.615610 1.536190

H -2.625029 0.872726 1.156758

Cl 1.008481 4.856061 0.252480

Cl -2.111415 3.448393 -0.485870

C -3.152363 -2.348273 0.295802

C -4.201807 -3.195330 -0.038934

C -5.461700 -2.673910 -0.307388

C -5.673405 -1.301904 -0.232996

C -4.626761 -0.455356 0.107775

H -2.169879 -2.752312 0.517654

H -4.036909 -4.266392 -0.087733

H -6.280486 -3.336201 -0.566330

H -6.657927 -0.891923 -0.430055

H -4.800967 0.614977 0.181915

H -1.662231 -0.879014 2.356978

N 2.523259 -2.655849 -0.090311

C 3.007468 -3.695657 -0.081386

C 3.617067 -5.002104 -0.070389

H 2.873681 -5.756370 -0.338487

H 4.436613 -5.030556 -0.792224

H 4.006402 -5.217139 0.927449

58

**d-H** E(gas)=-2299.75866031 E(CH3CN)= -2299.823056 G(CH3CN)=-2299.432481

Au -0.196453 -0.248291 -0.364377

C 1.519357 -1.280464 0.058509

N 2.795903 -0.860156 -0.098856

N 1.610953 -2.538922 0.544194

C 3.678059 -1.846746 0.289618

C 2.931317 -2.904651 0.694126

C 0.484537 -3.394335 0.869422

H 0.541342 -4.321409 0.296732

H 0.484473 -3.625155 1.936056

H -0.425608 -2.855471 0.609965

C 3.181044 0.474895 -0.534060

C 3.577443 1.382892 0.628804

H 4.005306 0.391682 -1.244209

H 2.316710 0.900709 -1.047067

C 4.036519 2.720995 0.094869

O 2.442961 1.469879 1.461357

H 4.414352 0.915370 1.167455

Cl 3.424722 -4.418311 1.265097

Cl 5.362801 -1.707649 0.192376

C 3.115449 3.738624 -0.146525

C 3.538738 4.956788 -0.663750

C 4.883043 5.165966 -0.948151

C 5.806139 4.154666 -0.708114

C 5.384125 2.938767 -0.186079

H 2.069200 3.573590 0.089505

H 2.817740 5.747506 -0.842715

H 5.212795 6.118309 -1.348959

H 6.857827 4.316961 -0.918023

H 6.111157 2.156379 0.015819

H 2.664719 2.033233 2.207142

C -1.891864 0.801508 -0.759820

C -3.147819 0.358837 -0.866434

C -3.680463 -1.014985 -0.875304

H -1.778956 1.887280 -0.858127

C -4.918119 -1.337460 -0.304951

C -5.398283 -2.640685 -0.346128

C -4.655845 -3.641146 -0.958771

C -3.429485 -3.329908 -1.537041

C -2.947675 -2.030067 -1.502080

H -5.503869 -0.583254 0.211372

H -6.353789 -2.873786 0.110843

H -5.034231 -4.656799 -0.993639

H -2.854178 -4.101211 -2.038098

H -2.005863 -1.779799 -1.978183

N -4.227511 1.395103 -1.071163

H -4.919333 1.015124 -1.723287

C -4.949835 1.905642 0.120508

H -3.780939 2.184325 -1.543016

C -6.258493 2.330130 -0.050899

C -6.941330 2.846630 1.042704

C -6.310385 2.932933 2.278327

C -4.997677 2.501060 2.424397

C -4.301046 1.980123 1.339831

H -6.747928 2.260129 -1.018476

H -7.966493 3.178585 0.926060

H -6.845981 3.334713 3.130911

H -4.508111 2.563548 3.389506

H -3.282228 1.624480 1.443062

67

**d-*i*pr** E(gas)=-2417.58578196 E(CH3CN)=-2417.646095 G(CH3CN)=-2417.175461

Au 0.342484 -0.343194 -0.208179

C 2.187593 -1.188364 0.058238

N 3.397476 -0.626447 -0.167894

N 2.447412 -2.443579 0.488554

C 4.405427 -1.521976 0.123240

C 3.806598 -2.666936 0.536462

C 1.444217 -3.426197 0.855185

H 1.563825 -4.326343 0.250247

H 1.538535 -3.682682 1.911756

H 0.466157 -2.984515 0.669360

C 3.604940 0.754273 -0.580778

C 3.987515 1.667298 0.582780

H 4.377898 0.781637 -1.350477

H 2.663989 1.097465 -1.014934

C 4.260585 3.061241 0.064001

O 2.915122 1.607050 1.495588

H 4.907252 1.279171 1.043942

Cl 4.498048 -4.133916 1.017039

Cl 6.054740 -1.196762 -0.080893

C 3.223134 3.982508 -0.066484

C 3.474549 5.252334 -0.571606

C 4.761784 5.609694 -0.954799

C 5.800549 4.694875 -0.825676

C 5.550638 3.427527 -0.315526

H 2.222504 3.701419 0.245353

H 2.663552 5.967105 -0.663701

H 4.957578 6.602050 -1.346255

H 6.808908 4.972653 -1.112966

H 6.368799 2.721191 -0.200842

H 3.128701 2.171545 2.242878

C -1.488717 0.508988 -0.441407

C -2.675615 -0.083777 -0.598833

C -3.011028 -1.507766 -0.782022

H -1.528589 1.602602 -0.384696

C -4.178987 -2.070822 -0.252155

C -4.471513 -3.414697 -0.451972

C -3.607708 -4.217569 -1.184725

C -2.449474 -3.666460 -1.723138

C -2.155530 -2.325026 -1.530251

H -4.852427 -1.475606 0.357228

H -5.374686 -3.835286 -0.023405

H -3.839027 -5.265207 -1.342583

H -1.780167 -4.281398 -2.315465

H -1.266604 -1.888352 -1.972340

N -3.891896 0.805067 -0.661074

H -4.546033 0.399581 -1.336209

C -4.638836 1.086864 0.595265

H -3.579849 1.693178 -1.059289

C -5.936933 1.594287 0.483755

C -6.596561 1.859629 1.683850

C -5.991172 1.637507 2.914206

C -4.696119 1.144481 2.979996

C -4.007788 0.867284 1.806722

C -6.603376 1.812285 -0.863753

H -7.604308 2.256676 1.656346

H -6.536156 1.856769 3.825650

H -4.217503 0.974134 3.937255

H -2.996120 0.478500 1.827556

C -7.205089 0.502081 -1.391760

C -7.663146 2.910889 -0.846293

H -5.835257 2.148354 -1.575983

H -8.000578 3.113948 -1.865323

H -8.543507 2.612209 -0.270645

H -7.275532 3.841691 -0.425284

H -7.638820 0.651618 -2.383761

H -6.479256 -0.316737 -1.466331

H -7.998455 0.158076 -0.722402

58

**d-Cl** E(gas)=-2759.21587726 E(CH3CN)=-2759.282623 G(gas)=-2758.903326

Au 0.230886 -0.391862 -0.413245

C 2.001262 -1.190333 0.231933

N 3.239393 -0.657827 0.113731

N 2.175056 -2.371726 0.865798

C 4.179074 -1.497425 0.674774

C 3.508379 -2.577698 1.147825

C 1.114202 -3.300680 1.209620

H 1.304178 -4.272189 0.750617

H 1.053157 -3.416022 2.293083

H 0.179730 -2.889708 0.830052

C 3.527942 0.652141 -0.452849

C 3.722763 1.728059 0.613881

H 4.418785 0.578090 -1.078501

H 2.678042 0.917890 -1.084145

C 4.093205 3.035379 -0.049653

O 2.513389 1.787301 1.336227

H 4.548969 1.422180 1.271967

Cl 4.098413 -3.958994 1.925059

Cl 5.844132 -1.190687 0.681191

C 3.100998 3.912611 -0.483182

C 3.445244 5.098132 -1.120971

C 4.781684 5.413579 -1.335394

C 5.775511 4.542541 -0.903967

C 5.431909 3.360173 -0.261292

H 2.059847 3.665773 -0.302677

H 2.668142 5.780158 -1.449651

H 5.049502 6.340404 -1.830937

H 6.820201 4.789498 -1.059091

H 6.212488 2.689656 0.088849

H 2.609327 2.453961 2.021179

C -1.526004 0.432955 -1.018397

C -2.694298 -0.159113 -1.282903

C -3.054830 -1.585025 -1.361629

H -1.539853 1.526083 -1.098232

C -4.325354 -2.052014 -1.001398

C -4.640463 -3.401277 -1.105238

C -3.696609 -4.305794 -1.572151

C -2.434377 -3.851698 -1.941091

C -2.116604 -2.505835 -1.843555

H -5.072466 -1.375198 -0.597796

H -5.626064 -3.745015 -0.811113

H -3.945005 -5.357957 -1.656450

H -1.698615 -4.548690 -2.327986

H -1.142891 -2.147372 -2.159196

N -3.859808 0.745027 -1.613242

H -4.423550 0.289540 -2.336912

C -4.762180 1.154427 -0.510805

H -3.458576 1.584482 -2.036841

C -6.066270 1.499135 -0.832195

C -6.922949 1.919102 0.173765

C -6.456253 1.988391 1.482429

C -5.146407 1.640320 1.792120

C -4.287245 1.219167 0.786759

H -6.427043 1.443129 -1.855346

H -7.946688 2.189788 -0.052836

Cl -7.518808 2.507913 2.736369

H -4.801487 1.695153 2.817229

H -3.267216 0.931460 1.014163

60

**d-NO_2_** E(gas)=-2504.09818337 E(CH3CN)=-2504.172184 G(CH3CN)=-2503.782857

Au 0.386143 -0.428569 -0.433244

C 2.161262 -1.156551 0.278623

N 3.387045 -0.594642 0.170765

N 2.351218 -2.312383 0.953493

C 4.335394 -1.390732 0.778683

C 3.682647 -2.473411 1.271229

C 1.307721 -3.257945 1.305117

H 1.534224 -4.238913 0.884445

H 1.224322 -3.338040 2.390256

H 0.371806 -2.885952 0.890667

C 3.654323 0.704645 -0.430306

C 3.800537 1.816959 0.606477

H 4.558909 0.634180 -1.036353

H 2.810807 0.929826 -1.085416

C 4.157451 3.111603 -0.088883

O 2.573816 1.869922 1.299332

H 4.617747 1.550033 1.292063

Cl 4.290485 -3.812787 2.105494

Cl 5.991177 -1.039847 0.811395

C 3.157334 3.964165 -0.551960

C 3.491006 5.137462 -1.217625

C 4.824668 5.464784 -1.430826

C 5.826476 4.618141 -0.970089

C 5.493464 3.448513 -0.299367

H 2.118107 3.708717 -0.372647

H 2.707582 5.800751 -1.568865

H 5.084133 6.382085 -1.948038

H 6.869003 4.874742 -1.124042

H 6.280238 2.797989 0.073926

H 2.641395 2.553517 1.970879

C -1.377991 0.326017 -1.105676

C -2.516212 -0.311122 -1.394800

C -2.828807 -1.748519 -1.462940

H -1.425550 1.416687 -1.205009

C -4.094300 -2.251139 -1.134360

C -4.362617 -3.611364 -1.224580

C -3.375771 -4.491459 -1.647262

C -2.118052 -4.002164 -1.985233

C -1.846705 -2.645362 -1.900467

H -4.876444 -1.594277 -0.765201

H -5.345181 -3.982702 -0.955044

H -3.587374 -5.552357 -1.721329

H -1.348513 -4.680735 -2.337431

H -0.875427 -2.260408 -2.191227

N -3.703687 0.548262 -1.774662

H -4.231324 0.062699 -2.506352

C -4.649220 0.948082 -0.705817

H -3.319238 1.394914 -2.200214

C -5.956863 1.232460 -1.074012

C -6.854605 1.640618 -0.099386

C -6.406705 1.751407 1.206611

C -5.102489 1.468539 1.574194

C -4.202975 1.057981 0.599773

H -6.284217 1.137668 -2.105152

H -7.886527 1.871129 -0.332057

N -7.362255 2.185477 2.249863

H -4.808884 1.568417 2.611523

H -3.178142 0.812734 0.852787

O -6.934587 2.278285 3.377371

O -8.494864 2.413337 1.891803

61

**[d-e-H]^≠^** E(gas)=-2376.11077478 E(CH3CN)=-2376.176894 G(CH3CN)=-2375.767322

Au -0.223814 -0.440402 -0.150703

C 1.626716 -1.280420 0.079760

N 2.833582 -0.716476 -0.158787

N 1.893473 -2.536637 0.503306

C 3.846155 -1.611916 0.117312

C 3.253615 -2.758620 0.534078

C 0.896223 -3.520947 0.881811

H 1.012153 -4.421959 0.277472

H 1.001583 -3.775040 1.937921

H -0.084945 -3.082410 0.705031

C 3.033325 0.666035 -0.566924

C 3.438204 1.573622 0.593711

H 3.790601 0.699120 -1.351974

H 2.082997 1.010699 -0.978908

C 3.688639 2.972766 0.076016

O 2.389527 1.499694 1.531675

H 4.371666 1.188926 1.029245

Cl 3.952336 -4.226304 1.001549

Cl 5.492507 -1.284303 -0.107184

C 2.652113 3.902275 0.021002

C 2.881661 5.177196 -0.482342

C 4.145259 5.531038 -0.939860

C 5.183171 4.607519 -0.886400

C 4.955805 3.335625 -0.377275

H 1.671212 3.623480 0.391897

H 2.072642 5.899574 -0.512641

H 4.324452 6.527457 -1.328935

H 6.174180 4.882428 -1.231063

H 5.774745 2.623074 -0.320584

H 2.627279 2.041030 2.288729

C -2.075557 0.401714 -0.331121

C -3.292511 -0.217987 -0.340369

C -3.505831 -1.678462 -0.455806

H -2.149802 1.485399 -0.179739

C -4.510340 -2.293078 0.299730

C -4.715797 -3.664281 0.219171

C -3.936762 -4.435661 -0.633935

C -2.944119 -3.831303 -1.398738

C -2.724291 -2.464625 -1.307269

H -5.111261 -1.706499 0.989505

H -5.485374 -4.129181 0.825398

H -4.106329 -5.504272 -0.707579

H -2.345431 -4.426794 -2.079709

H -1.951502 -1.996081 -1.907142

N -4.501126 0.528694 -0.430975

H -5.317300 -0.071186 -0.484700

C -4.752694 1.717569 0.343323

H -4.023689 1.075856 -2.039427

C -5.650629 2.648134 -0.172412

C -5.962772 3.789646 0.555970

C -5.366704 4.011444 1.790554

C -4.468092 3.080421 2.299738

C -4.161336 1.929988 1.585135

H -6.124687 2.468178 -1.133743

H -6.671312 4.505542 0.154403

H -5.604897 4.904344 2.357357

H -4.008447 3.243188 3.268445

H -3.467088 1.201360 1.988668

O -3.212469 1.124954 -2.651054

H -2.536086 0.706241 -1.885318

H -2.995111 2.047180 -2.840215

70

**[d-e-*i*pr]^≠^** E(gas)=-2493.93613349 E(CH3CN)=-2493.999943 G(CH3CN)=-2493.511054

Au 0.331763 -0.621885 -0.134736

C 2.319468 -1.099477 -0.063761

N 3.376161 -0.291344 -0.313631

N 2.852237 -2.304770 0.239824

C 4.558991 -0.985095 -0.163478

C 4.228739 -2.253706 0.184450

C 2.092504 -3.492013 0.585455

H 2.335120 -4.304427 -0.101470

H 2.321364 -3.799312 1.607328

H 1.035030 -3.243810 0.505097

C 3.276536 1.131216 -0.603607

C 3.569265 2.004731 0.615593

H 3.961985 1.377881 -1.416244

H 2.253430 1.311151 -0.938883

C 3.484579 3.462817 0.222306

O 2.631102 1.631930 1.598216

H 4.591889 1.793268 0.960121

Cl 5.228291 -3.583251 0.491814

Cl 6.092184 -0.321004 -0.440375

C 2.273477 4.147017 0.305433

C 2.194594 5.478820 -0.083767

C 3.321613 6.134538 -0.564669

C 4.532251 5.456386 -0.649618

C 4.613117 4.127359 -0.254524

H 1.400068 3.632602 0.693010

H 1.250941 6.008786 -0.006900

H 3.259867 7.175024 -0.864611

H 5.417405 5.966925 -1.013387

H 5.565629 3.606189 -0.305731

H 2.800560 2.154497 2.386170

C -1.647615 -0.123990 -0.164890

C -2.736944 -0.936889 -0.037587

C -2.706908 -2.416892 -0.068398

H -1.896052 0.938186 -0.048786

C -3.522552 -3.142990 0.806207

C -3.496177 -4.531558 0.809532

C -2.672678 -5.214006 -0.076694

C -1.867859 -4.500771 -0.959517

C -1.878289 -3.113418 -0.952632

H -4.153768 -2.623317 1.522091

H -4.119730 -5.079493 1.507314

H -2.661991 -6.298393 -0.084218

H -1.236999 -5.029225 -1.666403

H -1.250333 -2.560398 -1.643134

N -4.058124 -0.405309 -0.047739

H -4.758255 -1.137568 0.004203

C -4.423643 0.760532 0.724602

H -3.814664 0.108090 -1.711728

C -5.425249 1.615943 0.248693

C -5.765564 2.714273 1.044742

C -5.141442 2.962222 2.255804

C -4.152702 2.097643 2.712456

C -3.800259 0.997907 1.949516

C -6.154045 1.393942 -1.063163

H -6.542112 3.391692 0.703839

H -5.429525 3.824708 2.846542

H -3.664148 2.273134 3.664351

H -3.030300 0.316445 2.295072

O -3.079226 0.243014 -2.405209

H -2.276296 0.008626 -1.692195

H -3.054218 1.167864 -2.683669

C -7.647049 1.137418 -0.842061

C -5.937477 2.558732 -2.033095

H -5.754783 0.486501 -1.534377

H -6.386252 2.342162 -3.006488

H -6.394656 3.477472 -1.656148

H -4.873856 2.778039 -2.182060

H -8.146087 0.929121 -1.792430

H -7.812071 0.286856 -0.175840

H -8.133575 2.008250 -0.393973

61

**[d-e-Cl]^≠^** E(gas)=-2835.56915093 E(CH3CN)=-2835.638285 G(CH3CN)=-2835.

240208

Au -0.236614 -0.713243 0.287459

C -2.223555 -1.005519 -0.096835

N -3.243662 -0.136555 0.092098

N -2.790785 -2.121700 -0.607719

C -4.438128 -0.704199 -0.301016

C -4.152570 -1.955035 -0.741298

C -2.076139 -3.330222 -0.975369

H -2.473879 -4.182769 -0.422504

H -2.175165 -3.513243 -2.046669

H -1.026714 -3.183805 -0.723279

C -3.090255 1.230668 0.567775

C -3.117819 2.258968 -0.561676

H -3.879489 1.444401 1.290370

H -2.125010 1.279670 1.075132

C -3.054446 3.653128 0.021211

O -2.026880 1.949465 -1.398482

H -4.065582 2.151435 -1.108836

Cl -5.188368 -3.152403 -1.336152

Cl -5.942730 0.061715 -0.171094

C -1.825470 4.262368 0.267341

C -1.774254 5.531681 0.830803

C -2.948637 6.199439 1.156899

C -4.177336 5.596917 0.911805

C -4.228983 4.330815 0.343481

H -0.912495 3.741879 -0.003179

H -0.814382 6.004343 1.010949

H -2.907696 7.191793 1.592660

H -5.096892 6.119016 1.153196

H -5.192063 3.870899 0.137405

H -2.030220 2.567551 -2.133818

C 1.754860 -0.401139 0.610805

C 2.781699 -1.296167 0.525793

C 2.628069 -2.764349 0.416582

H 2.097385 0.640742 0.635861

C 3.468840 -3.487025 -0.436823

C 3.328013 -4.862383 -0.568464

C 2.362665 -5.536808 0.168356

C 1.531205 -4.828256 1.029920

C 1.656311 -3.451711 1.149378

H 4.212523 -2.970604 -1.037768

H 3.973801 -5.406258 -1.248916

H 2.262170 -6.612710 0.076712

H 0.788457 -5.353064 1.621283

H 1.007354 -2.902363 1.823087

N 4.134187 -0.890652 0.724144

H 4.774302 -1.676989 0.686833

C 4.691760 0.287049 0.112689

H 3.744788 -0.491486 2.411083

C 5.786655 0.887278 0.727704

C 6.391535 1.999205 0.158574

C 5.883020 2.520089 -1.023591

C 4.789542 1.927734 -1.644722

C 4.198272 0.807902 -1.080012

H 6.187944 0.473887 1.648831

H 7.249520 2.462101 0.630573

Cl 6.618090 3.915738 -1.731667

H 4.410889 2.336496 -2.573754

H 3.350436 0.342666 -1.570133

O 2.943606 -0.357211 3.020888

H 2.218170 -0.474758 2.201024

H 2.934345 0.543256 3.371330

63

**[d-e-NO_2_]^≠^** E(gas)=-2580.45461137 E(CH3CN)=-2580.532282 G(CH3CN)=-2580.124934

Au -0.412665 -0.745729 0.312743

C -2.387507 -0.943132 -0.180606

N -3.384339 -0.047546 0.007089

N -2.966321 -2.006571 -0.782560

C -4.576038 -0.546081 -0.477466

C -4.312542 -1.780712 -0.974061

C -2.277663 -3.219884 -1.183075

H -2.728735 -4.084958 -0.694337

H -2.334629 -3.343156 -2.265872

H -1.236305 -3.125222 -0.878713

C -3.209310 1.284052 0.569119

C -3.128721 2.373387 -0.498801

H -4.033892 1.488740 1.253758

H -2.276848 1.265617 1.136423

C -3.058849 3.730819 0.164649

O -1.995725 2.070984 -1.280638

H -4.041187 2.330287 -1.110979

Cl -5.357680 -2.902579 -1.687080

Cl -6.056551 0.269392 -0.380781

C -1.829193 4.286236 0.512661

C -1.773751 5.522736 1.144706

C -2.945115 6.210276 1.438723

C -4.174574 5.660914 1.093030

C -4.229997 4.428061 0.456103

H -0.917819 3.751393 0.265959

H -0.812725 5.955026 1.403020

H -2.900604 7.177398 1.927568

H -5.090959 6.199347 1.309190

H -5.192447 4.010875 0.171197

H -1.933405 2.725147 -1.981418

C 1.571848 -0.519731 0.732939

C 2.569019 -1.449544 0.696703

C 2.376437 -2.912080 0.589227

H 1.948827 0.509559 0.785063

C 3.255611 -3.672828 -0.189828

C 3.079571 -5.044473 -0.316865

C 2.038378 -5.677171 0.350514

C 1.166851 -4.930922 1.137607

C 1.328871 -3.558209 1.252539

H 4.058666 -3.188898 -0.739107

H 3.756775 -5.618108 -0.939853

H 1.909394 -6.750347 0.262811

H 0.363597 -5.423737 1.674824

H 0.649771 -2.979128 1.869215

N 3.928893 -1.077259 0.938638

H 4.532379 -1.890076 1.012215

C 4.586020 -0.001221 0.257239

H 3.487244 -0.597994 2.619792

C 5.809455 0.431935 0.770532

C 6.509572 1.448417 0.142207

C 5.963808 2.030798 -0.989977

C 4.753138 1.612118 -1.516835

C 4.060778 0.587169 -0.891700

H 6.221524 -0.035925 1.659673

H 7.462698 1.800839 0.515797

N 6.692905 3.124493 -1.653096

H 4.374132 2.086356 -2.413345

H 3.119219 0.243254 -1.302406

O 2.661348 -0.478760 3.192742

H 1.968630 -0.601704 2.357120

H 2.623673 0.414696 3.560043

O 6.175692 3.618945 -2.630889

O 7.755055 3.451337 -1.170824

# References

[1] Gaussian 09, Revision A.02, M. J. Frisch, G. W. Trucks, H. B. Schlegel, G. E. Scuseria, M. A. Robb, J. R. Cheeseman, G. Scalmani, V. Barone, G. A. Petersson, H. Nakatsuji, X. Li, M. Caricato, A. Marenich, J. Bloino, B. G. Janesko, R. Gomperts, B. Mennucci, H. P. Hratchian, J. V. Ortiz, A. F. Izmaylov, J. L. Sonnenberg, D. Williams-Young, F. Ding, F. Lipparini, F. Egidi, J. Goings, B. Peng, A. Petrone, T. Henderson, D. Ranasinghe, V. G. Zakrzewski, J. Gao, N. Rega, G. Zheng, W. Liang, M. Hada, M. Ehara, K. Toyota, R. Fukuda, J. Hasegawa, M. Ishida, T. Nakajima, Y. Honda, O. Kitao, H. Nakai, T. Vreven, K. Throssell, J. A. Montgomery, Jr., J. E. Peralta, F. Ogliaro, M. Bearpark, J. J. Heyd, E. Brothers, K. N. Kudin, V. N. Staroverov, T. Keith, R. Kobayashi, J. Normand, K. Raghavachari, A. Rendell, J. C. Burant, S. S. Iyengar, J. Tomasi, M. Cossi, J. M. Millam, M. Klene, C. Adamo, R. Cammi, J. W. Ochterski, R. L. Martin, K. Morokuma, O. Farkas, J. B. Foresman, and D. J. Fox, Gaussian, Inc., Wallingford CT, 2016.

[2] C. Adamo, V. Barone, *Chem. Phys. Lett.* **1997**, *274*, 242–250.

[3] A. D. McLean, G. S. Chandler, *J. Chem. Phys.* **1980**, *72*, 5639–5648.

[4] R. Krishnan, J. S. Binkley, R. Seeger, J. A. Pople, *J. Chem. Phys.* **1980**, *72*, 650–654.

[5] A. Veillard, Ed. , *Quantum Chemistry: The Challenge of Transition Metals and Coordination Chemistry*, Springer Netherlands, Dordrecht, **1986**.

[6] P. Schwerdtfeger, M. Dolg, W. H. E. Schwarz, G. A. Bowmaker, P. D. W. Boyd, *J. Chem. Phys.* **1989**, *91*, 1762–1774.

[7] D. Andrae, U. H�u�ermann, M. Dolg, H. Stoll, H. Preu�, *Theor. Chim. Acta* **1990**, *77*, 123–141.

[8] U. Häussermann, M. Dolg, H. Stoll, H. Preuss, P. Schwerdtfeger, R. M. Pitzer, *Mol. Phys.* **1993**, *78*, 1211–1224.

[9] V. Barone, M. Cossi, *J. Phys. Chem. A* **1998**, *102*, 1995–2001.

[10] J. Tomasi, M. Persico, *Chem. Rev.* **1994**, *94*, 2027–2094.
